# Supplementary material for: Consumption of coffee and tea and risk of developing stroke, dementia, and poststroke dementia: A cohort study in the UK Biobank
Source: PLoS Med. 2021 Nov 16;18(11):e1003830. doi: 10.1371/journal.pmed.1003830 (PMC8594796; doi:10.1371/journal.pmed.1003830)
Supplement: S1 Text — (DOCX) [file pmed.1003830.s002.docx]

**Analysis plan—S1 Text. Original analysis plan and modifications for the presented analyses**

**Objectives**

To investigate the associations of coffee and tea separately and in combination with the risk of developing stroke, dementia, and post-stroke dementia.

**Design**

A population based prospective cohort study

**Setting**

UK Biobank study, UK

**Exclusions**

- Participants younger than 50 years old (n =132,168)
- Without information on tea or coffee intake (n =2074)
- With prevalent stroke or dementia (n =2583)

365,682 participants are ultimately included in the present study

**Exposures**

Coffee intake is assessed at baseline using a touchscreen questionnaire. Participants were asked, “*how many cups of coffee do you drink each day (including decaffeinated coffee)*?” Participants selected one of the following: “*Less than one*”, “*Do not know*”, “*Prefer not to answer*”, or specific number of cups of coffee drinking per day. If participants reported drinking more than 10 cups each day, they were asked to confirm their response.

Likewise, tea intake is assessed at baseline using a touchscreen questionnaire. Participants were asked, “*how many cups of tea do you drink each day (including black and green tea)*?” Participants selected one of the following: “*Less than one*”, “*Do not know*”, “*Prefer not to answer*”, or specific number of cups of coffee tea drinking per day. If participants reported drinking more than 10 cups each day, they were asked to confirm their response.

We define coffee and tea intake into the following categories: 0, 0.5–1, 2–3, and ≥ 4 cups/day. For the combination of coffee and tea intake, the comparator group is non-coffee and non-tea drinkers.

**Outcomes**

- **Stroke**
- Total stroke
- ischemic stroke
- hemorrhage stroke
- **Dementia**
- Total dementia
- Alzheimer’s disease
- vascular dementia

**Data collection**

Information on sex, age, ethnicity background, education level, income, smoking status, and alcohol status, are collected through a touchscreen questionnaire and interview. Diet components are derived from the Food Frequency Questionnaire, and body mass index (BMI) is obtained from physical measurements. Ethnicity is categorized as White, Black or Black British, Asian or Asian British, and others. Education is defined as college or university degree, A levels/AS levels or equivalent, O levels/GCSEs or equivalent, CSEs or equivalent, NVQ or HND or HNC or equivalent, other professional qualifications, or none of the above. Income is categorized as less than £18,000, 18,000 to 30,999, 31,000 to 51,999, 52,000 to 100,000, and greater than 100,000. Smoking status is categorized as never, former, and current. Similarly, alcohol status is categorized as never, former, and current. Physical activity was measured as minutes per week spent walking or engaged in moderate or vigorous activity according to the International Physical Activity Questionnaire (IPAQ), and is defined as low: <10 excess metabolic equivalent (MET)-hours per week, moderate: 10–49.9 excess MET-hours per week, and high: ≥50 excess MET-hours per week. Information on high density lipoprotein (HDL) and low density lipoprotein (LDL) levels were obtained from blood samples collected at study recruitment.

Information on disease history was derived from medical examinations, self-reported medical conditions, and hospital inpatient records, including data on admissions and diagnoses from the Hospital Episode Statistics in England (dating back to 1997), the Scottish Morbidity Record (dating back to 1981), and the Patient Episode Database in Wales (dating back to 1998). Information on death was obtained through linkage to national death registries from May 2006 to June 2020, and the main cause of death for each participant was identified based on International Classification of Diseases 10 (ICD-10) codes.

**Statistical analysis**

- Using analysis of variance for continuous variables and chi-squared tests for categorical variables. If continuous variables did not follow a normal distribution, the Mann–Whitney U–test was applied.
- Using restricted cubic spline models to evaluate the relationship between coffee, tea, and their combination with stroke, dementia, and post-stroke dementia.
- Using Cox proportional-hazard models to estimate the associations of coffee and tea separately or in combination with the incidence of stroke and dementia.
- Examine the multiplicative interaction between coffee and tea by incorporating the two variables (categorical) and their cross-product term in the same model.
- All statistical tests were two-tailed, and p-values <0.05 were considered statistically significant.

**Adjustment for confounding factors**

Sex, age, ethnicity, education, income, body mass index, physical activity, alcohol status, smoking status, diet pattern, sugar-sweetened beverages, high density lipoprotein, low density lipoprotein, history of cancer, history of diabetes, history of cardiovascular arterial disease, hypertension, coffee (in tea analysis), and tea (in coffee analysis).

**Data representation**

- Flowchart of participant selection
- Association of coffee and tea with stroke and dementia
- Association of coffee and tea with post-stroke dementia
- Baseline characteristics by coffee and tea intake in the UK Biobank cohort
- Restricted Cubic Spline models for the relationship between coffee, tea, and their combination with stroke, dementia, and post-stroke dementia.
- Coffee intake and incident stroke, dementia, and post-stroke dementia;
- Tea intake and incident stroke, dementia, and post-stroke dementia;
- Combination of coffee and tea intake on incident stroke, dementia, and post-stroke dementia.
- Analyze the associations of coffee and tea separately or in combination with the incidence of stroke and dementia.
- Analyze the associations of coffee and tea separately or in combination with the incidence of stroke and dementia.
- Analyze the associations of coffee and tea separately or in combination with the incidence of stroke and dementia.

**Additional analyses**

- Stratify by sex
- Exclude events occurring during the first two years of follow-up
- Exclude incident major prior diseases (e.g., diabetes, cardiovascular arterial disease, and cancer) at baseline

**Modifications to the original analysis plan for the presented analyses**

The presented analyses are consistent with those that were planned in the original analysis plan described above, with the exception of the modifications presented below.

According to the comments of reviewers, we made several modifications to the original analysis plan:

- We examined the associations among different coffee types and the risk of incident stroke and dementia;
- We added stratified analysis by age, smoking status, physical activity, BMI, diet quality, and alcohol intake;
- We performed the analysis for including participants younger than 50 years old;
- We added competing risk models.
