# Supplementary material for: Consumption of coffee and tea and risk of developing stroke, dementia, and poststroke dementia: A cohort study in the UK Biobank
Source: PLoS Med. 2021 Nov 16;18(11):e1003830. doi: 10.1371/journal.pmed.1003830 (PMC8594796; doi:10.1371/journal.pmed.1003830)
Supplement: S1 Table — (DOC) [file pmed.1003830.s003.doc]

**S1 Table.** Diet component definitions used in the UK Biobank study

| Source and definition of healthy diet | Self-reported UK Biobank field code |
| --- | --- |
| UK Biobank Food Frequency  Questionnaire at baseline;  1. Fruits: ≥ 3 servings/day  2. Vegetables: ≥ 3 servings/day  3. Fish: ≥2 servings/week  4. Processed meats: ≤1 serving/week  5. Unprocessed red meats: ≤ 1.5 servings/week  6. Whole grains: ≥ 3servings/day  7. Refined grains: ≤1.5 servings/day | 1309, 1319, 1289, 1299, 1329, 1339, 1349, 1369, 1379, 1389, 1438, 1448, 1458, 1468 |

A healthy diet was based on adherence to at least four of seven commonly eaten food groups following recommendations on dietary priorities for cardiometabolic health [1].

**Reference**

1. Mozaffarian D. Dietary and Policy Priorities for Cardiovascular Disease, Diabetes, and Obesity: A Comprehensive Review. Circulation. 2016;133(2):187-225. Epub 2016/01/10. doi: 10.1161/CIRCULATIONAHA.115.018585. PubMed PMID: 26746178; PubMed Central PMCID: PMCPMC4814348.
