# Supplementary material for: Consumption of coffee and tea and risk of developing stroke, dementia, and poststroke dementia: A cohort study in the UK Biobank
Source: PLoS Med. 2021 Nov 16;18(11):e1003830. doi: 10.1371/journal.pmed.1003830 (PMC8594796; doi:10.1371/journal.pmed.1003830)
Supplement: S2 Table — (DOC) [file pmed.1003830.s004.doc]

**S2 Table.** Detailed information on missing covariates

| Covariates | N* | Missing rate (%) |
| --- | --- | --- |
| HDL | 52210 | 14.28 |
| LDL | 24153 | 6.60 |
| Diet | 14567 | 3.98 |
| Ethnicity | 1263 | 0.35 |
| BMI | 1880 | 0.51 |
| Smoking status | 1492 | 0.41 |
| Alcohol status | 359 | 0.10 |
| Physical activity | 65295 | 17.86 |
| Qualification | 6662 | 1.82 |
| Income | 60869 | 16.65 |

Abbreviations: BMI, body mass index (calculated as weight in kilograms divided by height in meters squared); HDL, high density lipoprotein; LDL, low density lipoprotein.

*N represents the number of missing responses.
