# Supplementary material for: Consumption of coffee and tea and risk of developing stroke, dementia, and poststroke dementia: A cohort study in the UK Biobank
Source: PLoS Med. 2021 Nov 16;18(11):e1003830. doi: 10.1371/journal.pmed.1003830 (PMC8594796; doi:10.1371/journal.pmed.1003830)
Supplement: S3 Table — (DOC) [file pmed.1003830.s005.doc]

**S3 Table. Baseline characteristics by the combination of coffee and tea intake in the UK Biobank cohort**

|  | Coffee and tea intake, No. (%) | | | *P* value |
| --- | --- | --- | --- | --- |
| Characteristic | Never | Only one | Both |
| Age, mean (SD), y | 59.14 (5.24) | 60.11 (5.20) | 60.65 (5.14) | <0.001 |
| Sex, male | 2302 (34.3) | 49400 (43.9) | 115358 (46.8) | <0.001 |
| Coffee intake, mean (SD) | 0 | 1.58 (2.52) | 2.27 (1.71) | <0.001 |
| Tea intake, mean (SD) | 0 | 3.18 (3.47) | 3.76 (2.49) | <0.001 |
| HDL, mean (SD), mmol/L | 1.44 (0.38) | 1.44 (0.39) | 1.47 (0.39) | <0.001 |
| LDL, mean (SD), mmol/L | 3.59 (0.90) | 3.57 (0.90) | 3.61 (0.88) | <0.001 |
| Health diet | 3152 (47.0) | 48207 (42.8) | 108658 (44.1) | <0.001 |
| Hypertension | 1909 (28.5) | 37862 (33.6) | 75539 (30.7) | <0.001 |
| Ethnicity |  |  |  | <0.001 |
| White | 6178 (92.2) | 106315 (94.4) | 238908 (97.0) |  |
| Asian or Asian British | 52 (0.8) | 505 (0.4) | 879 (0.4) |  |
| Black or Black British | 102 (1.5) | 2820 (2.5) | 2649 (1.1) |  |
| Other ethnic group | 254 (3.8) | 1699 (1.5) | 1987 (0.8) |  |
| BMI (kg/m^2^) |  |  |  | <0.001 |
| <25 | 2234 (33.3) | 32901 (29.2) | 79515 (32.3) |  |
| 25 to <30 | 2498 (37.3) | 47509 (42.2) | 109089 (44.3) |  |
| 30 to <35 | 1253 (18.7) | 22485 (20.0) | 42722 (17.3) |  |
| ≥35 | 718 (10.7) | 9694 (8.6) | 15064 (6.1) |  |
| Smoking status |  |  |  | <0.001 |
| Never | 4386 (65.4) | 57910 (51.4) | 129802 (52.7) |  |
| Former | 1917 (28.6) | 42437 (37.7) | 94897 (38.5) |  |
| Current | 400 (6.0) | 12242 (10.9) | 21691 (8.8) |  |
| Alcohol status |  |  |  | <0.001 |
| Never | 1018 (15.2) | 7316 (6.5) | 7716 (3.1) |  |
| Former | 730 (10.9) | 5953 (5.3) | 6956 (2.8) |  |
| Current | 4955 (73.9) | 99320 (88.2) | 231718 (94.0) |  |
| Physical activity |  |  |  | <0.001 |
| Low | 1286 (19.2) | 22815 (20.3) | 43942 (17.8) |  |
| Moderate | 3256 (48.6) | 54189 (48.1) | 125968 (51.1) |  |
| High | 2161 (32.2) | 35585 (31.6) | 76480 (31.0) |  |
| Qualification |  |  |  | <0.001 |
| College or University | 2008 (30.0) | 28259 (25.1) | 80709 (32.8) |  |
| A levels/AS levels | 753 (11.2) | 10884 (9.7) | 26320 (10.7) |  |
| O levels/GCSEs | 1399 (20.9) | 23359 (20.7) | 50882 (20.7) |  |
| CSEs or equivalent | 291 (4.3) | 4981 (4.4) | 8761 (3.6) |  |
| NVQ or HND or HNC | 441 (6.6) | 8741 (7.8) | 17041 (6.9) |  |
| None of the above | 1811 (27.1) | 36365 (32.3) | 62677 (25.5) |  |
| Income |  |  |  | <0.001 |
| Less than £18,000 | 2070 (30.9) | 36020 (32.0) | 64387 (26.1) |  |
| 18,000 to 30,999 | 1896 (28.3) | 31777 (28.2) | 70291 (28.5) |  |
| 31,000 to 51,999 | 1520 (22.7) | 25314 (22.5) | 60138 (24.4) |  |
| 52,000 to 100,000 | 975 (14.5) | 16029 (14.2) | 40903 (16.6) |  |
| Greater than 100,000 | 242 (3.6) | 3449 (3.1) | 10671 (4.3) |  |

Abbreviations: A, Advanced; AS, Advanced Subsidiary; BMI, body mass index (calculated as weight in kilograms divided by height in meters squared); CSE, Certificate of Secondary Education; GCSE, General Certificate of Secondary Education; HDL, high density lipoprotein; HNC, Higher National Certificate; HND, Higher National Diploma; LDL, low density lipoprotein; NVQ, National Vocational Qualification; O, Ordinary; SD, standard deviation; UK Biobank, United Kingdom Biobank.
