# Supplementary material for: Consumption of coffee and tea and risk of developing stroke, dementia, and poststroke dementia: A cohort study in the UK Biobank
Source: PLoS Med. 2021 Nov 16;18(11):e1003830. doi: 10.1371/journal.pmed.1003830 (PMC8594796; doi:10.1371/journal.pmed.1003830)
Supplement: S4 Table — (DOC) [file pmed.1003830.s006.doc]

**S4** **Table.** Correlation between coffee and tea intake and other covariates

| Variates | Coffee intake | |  | Tea intake | |
| --- | --- | --- | --- | --- | --- |
|  | r | *P* value |  | r | *P* value |
| Coffee intake | 1.000 | - |  | -0.337 | <0.001 |
| Tea intake | -0.337 | <0.001 |  | 1.000 | - |
| Sex | 0.055 | <0.001 |  | 0.018 | <0.001 |
| Age | -0.010 | <0.001 |  | 0.015 | <0.001 |
| Ethnicity | -0.088 | <0.001 |  | -0.047 | <0.001 |
| Qualification | -0.028 | <0.001 |  | 0.078 | <0.001 |
| Income | 0.039 | <0.001 |  | -0.059 | <0.001 |
| BMI | 0.045 | <0.001 |  | -0.022 | <0.001 |
| Physical activity | -0.011 | <0.001 |  | 0.029 | <0.001 |
| Alcohol status | 0.059 | <0.001 |  | -0.007 | <0.001 |
| Smoking status | 0.121 | <0.001 |  | 0.023 | <0.001 |
| Diet pattern | -0.030 | <0.001 |  | -0.001 | 0.691 |
| Consumption of sugar-sweetened beverages | 0.013 | <0.001 |  | 0.007 | <0.001 |
| HDL | 0.002 | 0.184 |  | -0.001 | 0.569 |
| LDL | 0.034 | <0.001 |  | -0.025 | <0.001 |
| Cancer | -0.011 | <0.001 |  | 0.005 | 0.002 |
| Diabetes | -0.005 | 0.002 |  | -0.024 | <0.001 |
| CAD | -0.012 | 0.021 |  | 0.004 | <0.001 |
| Hypertension | -0.023 | <0.001 |  | -0.002 | 0.199 |

Abbreviations: BMI, body mass index; CAD, coronary artery disease; HDL, high density lipoprotein; LDL, low density lipoprotein; UK Biobank, United Kingdom Biobank.
