# Supplementary material for: Consumption of coffee and tea and risk of developing stroke, dementia, and poststroke dementia: A cohort study in the UK Biobank
Source: PLoS Med. 2021 Nov 16;18(11):e1003830. doi: 10.1371/journal.pmed.1003830 (PMC8594796; doi:10.1371/journal.pmed.1003830)
Supplement: S5 Table — (DOC) [file pmed.1003830.s007.doc]

**S5 Table**. Association of coffee and tea with stroke in the UK Biobank cohort (unadjusted models)

| Groups | | Total | Stroke | | |  | Ischemic stroke | | |  | Hemorrhage stroke | | | |
| --- | --- | --- | --- | --- | --- | --- | --- | --- | --- | --- | --- | --- | --- | --- |
|  |  |  | Cases | HR (95% CI) | *P* value |  | Cases | HR (95% CI) | *P* value |  | Cases | HR (95% CI) | | *P* value |
| Coffee (cups/d) | | | | | | | | | | | | |  | |
| 0 |  | 75986 | 2286 | 1.00 (Ref) |  |  | 1288 | 1.00 (Ref) |  |  | 397 | 1.00 (Ref) | |  |
| 0.5-1 |  | 102404 | 2674 | 0.87 (0.82-0.92) | <0.001 |  | 1476 | 0.85 (0.79-0.92) | <0.001 |  | 499 | 0.93 (0.82-1.07) | | 0.308 |
| 2-3 |  | 116844 | 3032 | 0.86 (0.82-0.91) | <0.001 |  | 1679 | 0.85 (0.79-0.91) | <0.001 |  | 557 | 0.91 (0.80-1.04) | | 0.164 |
| ≥4 |  | 70448 | 2061 | 0.97 (0.92-1.03) | 0.351 |  | 1187 | 0.99 (0.92-1.08) | 0.875 |  | 362 | 0.98 (0.85-1.13) | | 0.818 |
| Tea (cups/d) | | | | | | | | | | | | | | |
| 0 |  | 50009 | 1565 | 1.00 (Ref) |  |  | 883 | 1.00 (Ref) |  |  | 252 | 1.00 (Ref) | |  |
| 0.5-1 |  | 39311 | 1123 | 0.91 (0.85-0.99) | 0.019 |  | 592 | 0.85 (0.77-0.95) | 0.003 |  | 223 | 1.13 (0.94-1.35) | | 0.199 |
| 2-3 |  | 107931 | 2788 | 0.83 (0.78-0.88) | <0.001 |  | 1568 | 0.82 (0.76-0.89) | <0.001 |  | 521 | 0.96 (0.82-1.11) | | 0.571 |
| ≥4 |  | 168431 | 4577 | 0.87 (0.82-0.92) | <0.001 |  | 2587 | 0.87 (0.81-0.94) | <0.001 |  | 819 | 0.97 (0.84-1.11) | | 0.620 |
| Coffee  (cups/d) | Tea  (cups/d) |  |  |  |  |  |  |  |  |  |  |  | |  |
| 0 | 0 | 6703 | 218 | 1.00 (Ref) |  |  | 129 | 1.00 (Ref) |  |  | 37 | 1.00 (Ref) | |  |
| 0 | 0.5-1 | 3285 | 104 | 0.95 (0.75-1.20) | 0.677 |  | 56 | 0.87 (0.63-1.18) | 0.367 |  | 21 | 1.17 (0.68-1.99) | | 0.576 |
| 0 | 2-3 | 15983 | 466 | 0.82 (0.70-0.96) | 0.016 |  | 261 | 0.78 (0.63-0.96) | 0.019 |  | 89 | 1.01 (0.69-1.48) | | 0.968 |
| 0 | ≥4 | 50015 | 1498 | 0.83 (0.72-0.95) | 0.009 |  | 842 | 0.79 (0.65-0.95) | 0.011 |  | 250 | 0.90 (0.64-1.28) | | 0.563 |
| 0.5-1 | 0 | 4509 | 158 | 0.98 (0.80-1.21) | 0.871 |  | 87 | 0.91 (0.70-1.20) | 0.519 |  | 22 | 0.89 (0.52-1.50) | | 0.652 |
| 0.5-1 | 0.5-1 | 7938 | 256 | 0.90 (0.75-1.07) | 0.235 |  | 121 | 0.72 (0.56-0.92) | 0.008 |  | 56 | 1.28 (0.85-1.94) | | 0.239 |
| 0.5-1 | 2-3 | 30399 | 816 | 0.70 (0.60-0.81) | <0.001 |  | 442 | 0.64 (0.52-0.77) | <0.001 |  | 160 | 0.95 (0.67-1.36) | | 0.796 |
| 0.5-1 | ≥4 | 59558 | 1444 | 0.63 (0.55-0.73) | <0.001 |  | 826 | 0.61 (0.51-0.74) | <0.001 |  | 261 | 0.79 (0.56-1.12) | | 0.186 |
| 2-3 | 0 | 14458 | 422 | 0.80 (0.68-0.94) | <0.001 |  | 220 | 0.70 (0.56-0.87) | 0.001 |  | 69 | 0.86 (0.58-1.29) | | 0.468 |
| 2-3 | 0.5-1 | 15105 | 421 | 0.75 (0.63-0.88) | <0.001 |  | 227 | 0.68 (0.55-0.84) | <0.001 |  | 82 | 0.98 (0.66-1.45) | | 0.920 |
| 2-3 | 2-3 | 44868 | 1085 | 0.63 (0.54-0.73) | <0.001 |  | 614 | 0.60 (0.50-0.73) | <0.001 |  | 198 | 0.80 (0.56-1.13) | | 0.207 |
| 2-3 | ≥4 | 42413 | 1104 | 0.68 (0.59-0.78) | <0.001 |  | 618 | 0.64 (0.53-0.78) | <0.001 |  | 208 | 0.89 (0.63-1.26) | | 0.509 |
| ≥4 | 0 | 24339 | 767 | 0.88 (0.76-1.02) | 0.099 |  | 447 | 0.87 (0.71-1.06) | 0.154 |  | 124 | 0.92 (0.64-1.33) | | 0.663 |
| ≥4 | 0.5-1 | 12983 | 342 | 0.74 (0.62-0.87) | <0.001 |  | 188 | 0.68 (0.55-0.86) | <0.001 |  | 64 | 0.89 (0.59-1.33) | | 0.567 |
| ≥4 | 2-3 | 16681 | 421 | 0.70 (0.59-0.82) | <0.001 |  | 251 | 0.70 (0.57-0.87) | 0.001 |  | 74 | 0.80 (0.54-1.19) | | 0.269 |
| ≥4 | ≥4 | 16445 | 531 | 0.89 (0.76-1.04) | 0.129 |  | 301 | 0.85 (0.69-1.04) | 0.116 |  | 100 | 1.10 (0.76-1.61) | | 0.608 |

Abbreviations: CI, confidence interval; HR, hazard ratio; UK Biobank, United Kingdom Biobank.
