# Supplementary material for: Consumption of coffee and tea and risk of developing stroke, dementia, and poststroke dementia: A cohort study in the UK Biobank
Source: PLoS Med. 2021 Nov 16;18(11):e1003830. doi: 10.1371/journal.pmed.1003830 (PMC8594796; doi:10.1371/journal.pmed.1003830)
Supplement: S6 Table — (DOC) [file pmed.1003830.s008.doc]

**S6 Table. Association of coffee and tea with dementia in the UK Biobank cohort (unadjusted model)**

| Groups | | Total | Dementia | | |  | Alzheimer disease | | |  | Vascular dementia | | |
| --- | --- | --- | --- | --- | --- | --- | --- | --- | --- | --- | --- | --- | --- |
|  |  |  | Cases | HR (95% CI) | *P* value |  | Cases | HR (95% CI) | *P* value |  | Cases | HR (95% CI) | *P* value |
| Coffee (cups/d) | | | | | | | | | | | | |  |
| 0 |  | 75986 | 1109 | 1.00 (Ref) |  |  | 442 | 1.00 (Ref) |  |  | 295 | 1.00 (Ref) |  |
| 0.5-1 |  | 102404 | 1419 | 0.96 (0.88-1.03) | 0.250 |  | 612 | 1.03 (0.91-1.17) | 0.607 |  | 341 | 0.86 (0.74-1.01) | 0.062 |
| 2-3 |  | 116844 | 1554 | 0.92 (0.85-0.99) | 0.024 |  | 645 | 0.95 (0.84-1.08) | 0.431 |  | 371 | 0.82 (0.70-0.96) | 0.011 |
| ≥4 |  | 70448 | 987 | 0.96 (0.88-1.05) | 0.355 |  | 429 | 1.05 (0.92-1.20) | 0.498 |  | 216 | 0.79 (0.66-0.94) | 0.008 |
| Tea  (cups/d) |  |  |  |  |  |  |  |  |  |  |  |  |  |
| 0 |  | 50009 | 739 | 1.00 (Ref) |  |  | 311 | 1.00 (Ref) |  |  | 201 | 1.00 (Ref) |  |
| 0.5-1 |  | 39311 | 519 | 0.87 (0.77-0.97) | 0.012 |  | 208 | 0.85 (0.71-1.02) | 0.073 |  | 118 | 0.75 (0.60-0.94) | 0.012 |
| 2-3 |  | 107931 | 1493 | 0.85 (0.78-0.93) | <0.001 |  | 627 | 0.94 (0.82-1.07) | 0.354 |  | 331 | 0.77 (0.64-0.91) | 0.003 |
| ≥4 |  | 168431 | 2318 | 0.86 (0.79-0.93) | <0.001 |  | 982 | 0.94 (0.83-1.07) | 0.348 |  | 573 | 0.85 (0.72-1.00) | 0.046 |
| Coffee  (cups/d) | Tea  (cups/d) |  |  |  |  |  |  |  |  |  |  |  |  |
| 0 | 0 | 6703 | 111 | 1.00 (Ref) |  |  | 46 | 1.00 (Ref) |  |  | 35 | 1.00 (Ref) |  |
| 0 | 0.5-1 | 3285 | 52 | 0.91 (0.65-1.26) | 0.563 |  | 23 | 1.03 (0.63-1.70) | 0.904 |  | 18 | 0.99 (0.56-1.75) | 0.983 |
| 0 | 2-3 | 15983 | 262 | 0.86 (0.69-1.07) | 0.177 |  | 101 | 0.92 (0.65-1.31) | 0.658 |  | 60 | 0.62 (0.41-0.94) | 0.025 |
| 0 | ≥4 | 50015 | 684 | 0.69 (0.57-0.85) | <0.001 |  | 272 | 0.79 (0.58-1.08) | 0.139 |  | 182 | 0.58 (0.41-0.84) | 0.004 |
| 0.5-1 | 0 | 4509 | 72 | 0.83 (0.62-1.12) | 0.221 |  | 29 | 0.94 (0.59-1.50) | 0.792 |  | 23 | 0.84 (0.50-1.42) | 0.515 |
| 0.5-1 | 0.5-1 | 7938 | 111 | 0.72 (0.55-0.93) | 0.013 |  | 37 | 0.68 (0.44-1.05) | 0.085 |  | 23 | 0.47 (0.28-0.79) | 0.005 |
| 0.5-1 | 2-3 | 30399 | 439 | 0.67 (0.54-0.82) | <0.001 |  | 202 | 0.97 (0.71-1.34) | 0.868 |  | 103 | 0.49 (0.34-0.72) | <0.001 |
| 0.5-1 | ≥4 | 59558 | 797 | 0.63 (0.51-0.77) | <0.001 |  | 344 | 0.84 (0.62-1.15) | 0.282 |  | 192 | 0.48 (0.33-0.68) | <0.001 |
| 2-3 | 0 | 14458 | 215 | 0.75 (0.59-0.94) | 0.012 |  | 85 | 0.86 (0.60-1.23) | 0.395 |  | 64 | 0.70 (0.47-1.06) | 0.093 |
| 2-3 | 0.5-1 | 15105 | 187 | 0.60 (0.48-0.76) | <0.001 |  | 75 | 0.72 (0.50-1.04) | 0.082 |  | 42 | 0.43 (0.27-0.67) | <0.001 |
| 2-3 | 2-3 | 44868 | 581 | 0.60 (0.49-0.74) | <0.001 |  | 241 | 0.78 (0.57-1.08) | 0.132 |  | 127 | 0.42 (0.29-0.61) | <0.001 |
| 2-3 | ≥4 | 42413 | 571 | 0.63 (0.51-0.77) | <0.001 |  | 244 | 0.84 (0.62-1.16) | 0.290 |  | 138 | 0.48 (0.33-0.70) | <0.001 |
| ≥4 | 0 | 24339 | 341 | 0.73 (0.59-0.91) | 0.004 |  | 151 | 0.90 (0.65-1.26) | 0.540 |  | 79 | 0.54 (0.36-0.80) | 0.002 |
| ≥4 | 0.5-1 | 12983 | 169 | 0.68 (0.54-0.87) | 0.002 |  | 73 | 0.82 (0.56-1.18) | 0.281 |  | 35 | 0.45 (0.28-0.71) | <0.001 |
| ≥4 | 2-3 | 16681 | 211 | 0.65 (0.52-0.82) | <0.001 |  | 83 | 0.72 (0.50-1.04) | 0.078 |  | 41 | 0.40 (0.25-0.63) | <0.001 |
| ≥4 | ≥4 | 16445 | 266 | 0.82 (0.65-1.02) | 0.074 |  | 122 | 1.09 (0.77-1.53) | 0.631 |  | 61 | 0.59 (0.39-0.90) | 0.013 |

Abbreviations: CI, confidence interval; HR, hazard ratio, UK Biobank, United Kingdom Biobank.
