# Supplementary material for: Consumption of coffee and tea and risk of developing stroke, dementia, and poststroke dementia: A cohort study in the UK Biobank
Source: PLoS Med. 2021 Nov 16;18(11):e1003830. doi: 10.1371/journal.pmed.1003830 (PMC8594796; doi:10.1371/journal.pmed.1003830)
Supplement: S8 Table — (DOC) [file pmed.1003830.s010.doc]

**S8 Table**. Association of coffee and tea with post-stroke dementia in the UK Biobank cohort (unadjusted models)

| Groups | | Total | Dementia | | |  | Alzheimer disease | | |  | Vascular dementia | | |
| --- | --- | --- | --- | --- | --- | --- | --- | --- | --- | --- | --- | --- | --- |
|  |  |  | Cases | HR (95% CI) | *P* value |  | Cases | HR (95% CI) | *P* value |  | Cases | HR (95% CI) | *P* value |
| Coffee (cups/d) | | | | | | | | | | | | |  |
| 0 |  | 3105 | 174 | 1.00 (Ref) |  |  | 32 | 1.00 (Ref) |  |  | 78 | 1.00 (Ref) |  |
| 0.5-1 |  | 3439 | 166 | 0.79 (0.64-0.98) | 0.034 |  | 32 | 0.83 (0.51-1.36) | 0.462 |  | 88 | 0.93 (0.69-1.26) | 0.641 |
| 2-3 |  | 3933 | 171 | 0.73 (0.59-0.91) | 0.004 |  | 29 | 0.68 (0.41-1.12) | 0.129 |  | 86 | 0.82 (0.60-1.11) | 0.198 |
| ≥4 |  | 2794 | 127 | 0.83 (0.66-1.04) | 0.106 |  | 25 | 0.89 (0.53-1.50) | 0.664 |  | 59 | 0.86 (0.61-1.21) | 0.384 |
| Tea  (cups/d) |  |  |  |  |  |  |  |  |  |  |  |  |  |
| 0 |  | 2162 | 97 | 1.00 (Ref) |  |  | 17 | 1.00 (Ref) |  |  | 51 | 1.00 (Ref) |  |
| 0.5-1 |  | 1516 | 77 | 1.09 (0.81-1.47) | 0.579 |  | 14 | 1.13 (0.55-2.28) | 0.744 |  | 37 | 0.99 (0.65-1.51) | 0.954 |
| 2-3 |  | 3643 | 178 | 1.00 (0.78-1.28) | 0.989 |  | 31 | 0.99 (0.55-1.79) | 0.973 |  | 90 | 0.95 (0.67-1.34) | 0.765 |
| ≥4 |  | 5954 | 288 | 0.99 (0.78-1.24) | 0.898 |  | 56 | 1.09 (0.63-1.88) | 0.754 |  | 135 | 0.87 (0.63-1.20) | 0.393 |
| Coffee  (cups/d) | Tea  (cups/d) |  |  |  |  |  |  |  |  |  |  |  |  |
| 0 | 0 | 316 | 24 | 1.00 (Ref) |  |  | 5 | 1.00 (Ref) |  |  | 9 | 1.00 (Ref) |  |
| 0 | 0.5-1 | 149 | 10 | 0.81 (0.39-1.68) | 0.565 |  | 3 | 1.16 (0.28-4.85) | 0.840 |  | 8 | 1.69 (0.65-4.38) | 0.281 |
| 0 | 2-3 | 671 | 40 | 0.68 (0.41-1.13) | 0.137 |  | 9 | 0.74 (0.25-2.21) | 0.591 |  | 19 | 0.84 (0.38-1.86) | 0.671 |
| 0 | ≥4 | 1959 | 100 | 0.54 (0.35-0.84) | 0.007 |  | 15 | 0.39 (0.14-1.07) | 0.068 |  | 42 | 0.59 (0.29-1.22) | 0.154 |
| 0.5-1 | 0 | 206 | 12 | 0.62 (0.31-1.25) | 0.183 |  | 2 | 0.50 (0.10-2.59) | 0.410 |  | 7 | 0.95 (0.35-2.54) | 0.912 |
| 0.5-1 | 0.5-1 | 338 | 17 | 0.57 (0.30-1.06) | 0.074 |  | 1 | 0.16 (0.02-1.36) | 0.094 |  | 7 | 0.61 (0.23-1.63) | 0.323 |
| 0.5-1 | 2-3 | 1032 | 49 | 0.47 (0.29-0.77) | 0.003 |  | 10 | 0.46 (0.16-1.36) | 0.160 |  | 27 | 0.67 (0.31-1.42) | 0.296 |
| 0.5-1 | ≥4 | 1859 | 88 | 0.48 (0.30-0.75) | 0.001 |  | 19 | 0.50 (0.19-1.33) | 0.166 |  | 47 | 0.66 (0.32-1.35) | 0.257 |
| 2-3 | 0 | 578 | 21 | 0.39 (0.22-0.70) | 0.002 |  | 4 | 0.36 (0.10-1.33) | 0.124 |  | 14 | 0.67 (0.29-1.56) | 0.355 |
| 2-3 | 0.5-1 | 550 | 22 | 0.42 (0.23-0.75) | 0.003 |  | 6 | 0.55 (0.17-1.81) | 0.325 |  | 10 | 0.49 (0.20-1.21) | 0.123 |
| 2-3 | 2-3 | 1367 | 61 | 0.46 (0.29-0.74) | 0.001 |  | 8 | 0.29 (0.09-0.89) | 0.030 |  | 30 | 0.59 (0.28-1.24) | 0.161 |
| 2-3 | ≥4 | 1433 | 66 | 0.48 (0.30-0.77) | 0.002 |  | 11 | 0.39 (0.14-1.12) | 0.081 |  | 32 | 0.61 (0.29-1.28) | 0.190 |
| ≥4 | 0 | 1059 | 40 | 0.44 (0.26-0.73) | 0.001 |  | 6 | 0.32 (0.10-1.05) | 0.059 |  | 21 | 0.61 (0.28-1.33) | 0.210 |
| ≥4 | 0.5-1 | 476 | 27 | 0.64 (0.37-1.10) | 0.108 |  | 4 | 0.45 (0.12-1.69) | 0.239 |  | 11 | 0.68 (0.28-1.64) | 0.389 |
| ≥4 | 2-3 | 562 | 27 | 0.53 (0.31-0.92) | 0.025 |  | 4 | 0.38 (0.10-1.42) | 0.150 |  | 14 | 0.73 (0.31-1.68) | 0.457 |
| ≥4 | ≥4 | 690 | 33 | 0.52 (0.31-0.89) | 0.016 |  | 11 | 0.85 (0.29-2.45) | 0.761 |  | 13 | 0.54 (0.23-1.26) | 0.152 |

Abbreviations: CI, confidence interval; HR, hazard ratio, UK Biobank, United Kingdom Biobank.
