# Supplementary material for: Consumption of coffee and tea and risk of developing stroke, dementia, and poststroke dementia: A cohort study in the UK Biobank
Source: PLoS Med. 2021 Nov 16;18(11):e1003830. doi: 10.1371/journal.pmed.1003830 (PMC8594796; doi:10.1371/journal.pmed.1003830)
Supplement: S10 Table — (DOC) [file pmed.1003830.s012.doc]

**S10 Table. Risk of incident dementia according to coffee types in the UK Biobank**

| Coffee types | Dementia | | | |  | Alzheimer disease | | | |  | Vascular dementia | | | |
| --- | --- | --- | --- | --- | --- | --- | --- | --- | --- | --- | --- | --- | --- | --- |
|  | Unadjusted  HR (95% CI) | *P*  value | Multi-adjusted  HR (95% CI)^a^ | *P*  value |  | Unadjusted  HR (95% CI) | *P*  value | Multi-adjusted  HR (95% CI)^a^ | *P*  value |  | Unadjusted  HR (95% CI) | *P*  value | Multi-adjusted  HR (95% CI)^a^ | *P*  value |
| Ground coffee vs  instant coffee | 0.79 (0.74-0.85) | <0.001 | 0.83 (0.77-0.89) | <0.001 |  | 0.72 (0.65-0.81) | <0.001 | 0.77 (0.69-0.87) | <0.001 |  | 0.80 (0.69-0.94) | 0.006 | 0.82 (0.70-0.96) | 0.012 |
| Instant coffee  vs decaffeinated coffee | 0.87 (0.80-0.94) | <0.001 | 0.85 (0.79-0.92) | <0.001 |  | 0.80 (0.71-0.90) | <0.001 | 0.81 (0.72-0.91) | <0.001 |  | 0.89 (0.76-1.05) | 0.158 | 0.84 (0.72-0.99) | 0.036 |
| Ground coffee  vs decaffeinated coffee | 0.59 (0.54-0.66) | <0.001 | 0.74 (0.66-0.82) | <0.001 |  | 0.53 (0.45-0.62) | <0.001 | 0.67 (0.57-0.78) | <0.001 |  | 0.58 (0.47-0.72) | <0.001 | 0.74 (0.59-0.92) | 0.008 |

Abbreviations: CI, confidence interval; HR, hazard ratio; UK Biobank, United Kingdom Biobank.

^a^Multivariable model is adjusted for sex, age, ethnicity (White, Asian or Asian British, Black or Black British, and Other ethnic group), qualification (college or university degree, A levels/AS levels or equivalent, O levels/GCSEs or equivalent, CSEs or equivalent, NVQ or HND or HNC or equivalent, other professional qualifications, or none of the above), income (less than £18,000, 18,000 to 30,999, 31,000 to 51,999, 52,000 to 100,000, and greater than 100,000), BMI (<25, 25 to <30, 30 to <35, and ≥35 kg/m2), smoking status (never, former, current), alcohol status (never, former, and current), physical activity (low, moderate, and high), diet pattern (health and unhealth, created by fruits, vegetables, fish, processed meats, unprocessed red meats, whole grains, refined grains), consumption of sugar-sweetened beverages, tea intake, HDL, LDL, cancer, diabetes, CAD, and hypertension.
