# Supplementary material for: Consumption of coffee and tea and risk of developing stroke, dementia, and poststroke dementia: A cohort study in the UK Biobank
Source: PLoS Med. 2021 Nov 16;18(11):e1003830. doi: 10.1371/journal.pmed.1003830 (PMC8594796; doi:10.1371/journal.pmed.1003830)
Supplement: S31 Table — (DOC) [file pmed.1003830.s033.doc]

**S31 Table.** Association of coffee and tea with stroke in the UK Biobank cohort (detail adjusting for smoking and alcohol statuses)

| Factors | | HR (95% CI)^a^ | P value |  | HR (95% CI)^b^ | *P* value |
| --- | --- | --- | --- | --- | --- | --- |
| Male vs female | | 1.42 (1.36-1.48) | <0.001 |  | 1.42 (1.36-1.48) | <0.001 |
| Age | | 1.10 (1.09-1.10) | <0.001 |  | 1.10 (1.09-1.10) | <0.001 |
| Income | |  |  |  |  |  |
| Less than £18,000 | | 1.00 (Ref) |  |  | 1.00 (Ref) |  |
| 18,000 to 30,999 | | 0.91 (0.86-0.95) | <0.001 |  | 0.91 (0.87-0.96) | <0.001 |
| 31,000 to 51,999 | | 0.83 (0.78-0.88) | <0.001 |  | 0.84 (0.79-0.89) | <0.001 |
| 52,000 to 100,000 | | 0.76 (0.70-0.82) | <0.001 |  | 0.77 (0.71-0.83) | <0.001 |
| Greater than 100,000 | | 0.71 (0.62-0.82) | <0.001 |  | 0.72 (0.62-0.83) | <0.001 |
| Qualification | |  |  |  |  |  |
| College or University | | 1.00 (Ref) |  |  | 1.00 (Ref) |  |
| A levels/AS levels | | 1.07 (0.99-1.16) | 0.072 |  | 1.07 (0.99-1.15) | 0.090 |
| O levels/GCSEs | | 1.06 (1.00-1.13) | 0.061 |  | 1.06 (0.99-1.12) | 0.084 |
| CSEs or equivalent | | 1.17 (1.04-1.31) | 0.009 |  | 1.16 (1.03-1.30) | 0.013 |
| NVQ or HND or HNC | | 1.05 (0.97-1.14) | 0.248 |  | 1.04 (0.96-1.13) | 0.322 |
| None of the above | | 1.12 (1.06-1.19) | <0.001 |  | 1.11 (1.05-1.18) | <0.001 |
| Ethnicity | |  |  |  |  |  |
| White | | 1.00 (Ref) |  |  | 1.00 (Ref) |  |
| Asian or Asian British | | 0.98 (0.70-1.37) | 0.915 |  | 0.99 (0.71-1.38) | 0.943 |
| Black or Black British | | 1.69 (1.45-1.96) | <0.001 |  | 1.71 (1.47-1.99) | <0.001 |
| Other ethnic group | | 1.04 (0.84-1.29) | 0.723 |  | 1.04 (0.84-1.30) | 0.701 |
| Physical activity | |  |  |  |  |  |
| Low | | 1.00 (Ref) |  |  | 1.00 (Ref) |  |
| Moderate | | 0.90 (0.85-0.95) | <0.001 |  | 0.90 (0.86-0.95) | <0.001 |
| High | | 0.95 (0.90-1.01) | 0.091 |  | 0.96 (0.91-1.01) | 0.130 |
| BMI (kg/m^2^) | |  |  |  |  |  |
| <25 | | 1.00 (Ref) |  |  | 1.00 (Ref) |  |
| 25 to <30 | | 0.98 (0.94-1.03) | 0.518 |  | 0.99 (0.94-1.04) | 0.595 |
| 30 to <35 | | 1.01 (0.95-1.08) | 0.681 |  | 1.01 (0.96-1.08) | 0.628 |
| ≥35 | | 1.12 (1.03-1.21) | 0.005 |  | 1.12 (1.03-1.21) | 0.006 |
| HDL | | 0.97 (0.91-1.04) | 0.417 |  | 0.98 (0.91-1.05) | 0.501 |
| LDL | | 1.00 (0.98-1.03) | 0.937 |  | 1.00 (0.98-1.03) | 0.958 |
| Health diet | | 0.96 (0.92-1.00) | 0.074 |  | 0.97 (0.93-1.01) | 0.128 |
| Consumption of  sugar-sweetened beverages | | 0.97 (0.92-1.02) | 0.178 |  | 0.96 (0.92-1.01) | 0.146 |
| Diabetes | |  |  |  |  |  |
| No | | 1.00 (Ref) |  |  | 1.00 (Ref) |  |
| Yes | | 1.55 (1.46-1.65) | <0.001 |  | 1.55 (1.45-1.64) | <0.001 |
| Cancer | |  |  |  |  |  |
| No | | 1.00 (Ref) |  |  | 1.00 (Ref) |  |
| Yes | | 1.18 (1.11-1.26) | <0.001 |  | 1.18 (1.11-1.26) | <0.001 |
| CAD | |  |  |  |  |  |
| No | | 1.00 (Ref) |  |  | 1.00 (Ref) |  |
| Yes | | 1.61 (1.51-1.72) | <0.001 |  | 1.61 (1.51-1.72) | <0.001 |
| Hypertension | |  |  |  |  |  |
| No | | 1.00 (Ref) |  |  | 1.00 (Ref) |  |
| Yes | | 1.41 (1.35-1.47) | <0.001 |  | 1.40 (1.35-1.46) | <0.001 |
| Coffee (cups/d) | Tea (cups/d) |  |  |  |  |  |
| 0 0 | | 1.00 (Ref) |  |  | 1.00 (Ref) |  |
| 0 0.5-1 | | 0.87 (0.69-1.11) | 0.263 |  | 0.87 (0.69-1.11) | 0.261 |
| 0 2-3 | | 0.77 (0.65-0.90) | 0.002 |  | 0.77 (0.66-0.91) | 0.002 |
| 0 ≥4 | | 0.78 (0.67-0.89) | <0.001 |  | 0.77 (0.67-0.89) | <0.001 |
| 0.5-1 0 | | 1.00 (0.82-1.23) | 0.980 |  | 1.00 (0.82-1.23) | 0.974 |
| 0.5-1 0.5-1 | | 0.93 (0.77-1.11) | 0.407 |  | 0.93 (0.78-1.12) | 0.441 |
| 0.5-1 2-3 | | 0.73 (0.63-0.85) | <0.001 |  | 0.74 (0.64-0.86) | <0.001 |
| 0.5-1 ≥4 | | 0.66 (0.57-0.76) | <0.001 |  | 0.66 (0.57-0.76) | <0.001 |
| 2-3 0 | | 0.79 (0.67-0.93) | 0.005 |  | 0.79 (0.67-0.93) | 0.005 |
| 2-3 0.5-1 | | 0.80 (0.67-0.94) | 0.007 |  | 0.80 (0.68-0.94) | 0.008 |
| 2-3 2-3 | | 0.68 (0.59-0.79) | <0.001 |  | 0.69 (0.59-0.79) | <0.001 |
| 2-3 ≥4 | | 0.70 (0.61-0.82) | <0.001 |  | 0.71 (0.61-0.82) | <0.001 |
| ≥4 0 | | 0.82 (0.70-0.95) | 0.008 |  | 0.81 (0.69-0.94) | 0.005 |
| ≥4 0.5-1 | | 0.74 (0.63-0.88) | 0.001 |  | 0.74 (0.63-0.88) | 0.001 |
| ≥4 2-3 | | 0.69 (0.59-0.82) | <0.001 |  | 0.69 (0.59-0.82) | <0.001 |
| ≥4 ≥4 | | 0.83 (0.71-0.98) | 0.025 |  | 0.83 (0.71-0.97) | 0.023 |
| Smoking status | |  |  |  |  |  |
| Never | | 1.00 (Ref) |  |  | 1.00 (Ref) |  |
| former smokers who have  quit > 5 years | | 1.05 (1.00-1.10) | 0.032 |  | 1.05 (1.00-1.10) | 0.059 |
| Former smokers who have  ≤5 years | |  |  |  | 1.04 (0.98-1.11) | 0.157 |
| Current <10 cigarettes/day | | 1.75 (1.64-1.86) | <0.001 |  | 1.66 (1.42-1.94) | <0.001 |
| Current 10–20 cigarettes/day | |  |  |  | 1.80 (1.63-1.99) | <0.001 |
| Current 20+ cigarettes/day | |  |  |  | 2.24 (2.04-2.47) | <0.001 |
| Alcohol status | |  |  |  |  |  |
| Never | | 1.00 (Ref) |  |  | 1.00 (Ref) |  |
| Former | | 1.04 (0.92-1.17) | 0.537 |  | 1.03 (0.92-1.16) | 0.614 |
| Current <7 g/day | | 0.81 (0.75-0.89) | <0.001 |  | 0.83 (0.76-0.91) | <0.001 |
| Current 7-16 g/day | |  |  |  | 0.77 (0.70-0.84) | <0.001 |
| Current >16 g/day | |  |  |  | 0.83 (0.64-1.08) | 0.174 |

^a^Multivariable model is adjusted for age, ethnicity (White, Asian or Asian British, Black or Black British, and Other ethnic group), qualification (college or university degree, A levels/AS levels or equivalent, O levels/GCSEs or equivalent, CSEs or equivalent, NVQ or HND or HNC or equivalent, other professional qualifications, or none of the above), income (less than £18,000, 18,000 to 30,999, 31,000 to 51,999, 52,000 to 100,000, and greater than 100,000), BMI (<25, 25 to <30, 30 to <35, and ≥35 kg/m^2^), physical activity (low, moderate, and high), diet pattern (health and unhealth, created by fruits, vegetables, fish, processed meats, unprocessed red meats, whole grains, refined grains), consumption of sugar-sweetened beverages, HDL, LDL, cancer, diabetes, CAD, hypertension, smoking status (never, former, current), and alcohol status (never, former, and current).

^b^Multivariable model is adjusted for age, ethnicity (White, Asian or Asian British, Black or Black British, and Other ethnic group), qualification (college or university degree, A levels/AS levels or equivalent, O levels/GCSEs or equivalent, CSEs or equivalent, NVQ or HND or HNC or equivalent, other professional qualifications, or none of the above), income (less than £18,000, 18,000 to 30,999, 31,000 to 51,999, 52,000 to 100,000, and greater than 100,000), BMI (<25, 25 to <30, 30 to <35, and ≥35 kg/m^2^), physical activity (low, moderate, and high), diet pattern (health and unhealth, created by fruits, vegetables, fish, processed meats, unprocessed red meats, whole grains, refined grains), consumption of sugar-sweetened beverages, HDL, LDL, cancer, diabetes, CAD, hypertension, smoking status (never smokers, former smokers who have quit >5 years ago, former smokers who have quit ≤ 5 years, current smokers <10 cigarettes per day, current smokers 10–20 cigarettes per day, and current smokers 20+ cigarettes per day), and alcohol status (never drinkers, former drinkers, current drinkers <7g per day, current drinkers 7-16 g per day, and current drinkers >16 g per day).
