# Supplementary material for: Consumption of coffee and tea and risk of developing stroke, dementia, and poststroke dementia: A cohort study in the UK Biobank
Source: PLoS Med. 2021 Nov 16;18(11):e1003830. doi: 10.1371/journal.pmed.1003830 (PMC8594796; doi:10.1371/journal.pmed.1003830)
Supplement: S32 Table — (DOC) [file pmed.1003830.s034.doc]

**S32 Table. Association of coffee and tea with dementia in the UK Biobank cohort (detail adjusting for smoking and alcohol statuses)**

| Factors | | HR (95% CI)^a^ | P value |  | HR (95% CI)^b^ | P value |
| --- | --- | --- | --- | --- | --- | --- |
| Male vs female | | 1.26 (1.19-1.34) | <0.001 |  | 1.31 (1.23-1.39) | <0.001 |
| Age | | 1.19 (1.18-1.19) | <0.001 |  | 1.19 (1.18-1.19) | <0.001 |
| Income | |  |  |  |  |  |
| Less than £18,000 | | 1.00 (Ref) |  |  | 1.00 (Ref) |  |
| 18,000 to 30,999 | | 0.84 (0.78-0.89) | <0.001 |  | 0.84 (0.79-0.90) | <0.001 |
| 31,000 to 51,999 | | 0.68 (0.62-0.74) | <0.001 |  | 0.69 (0.63-0.75) | <0.001 |
| 52,000 to 100,000 | | 0.59 (0.52-0.67) | <0.001 |  | 0.61 (0.53-0.69) | <0.001 |
| Greater than 100,000 | | 0.60 (0.48-0.76) | <0.001 |  | 0.62 (0.49-0.78) | <0.001 |
| Qualification | |  |  |  |  |  |
| College or University | | 1.00 (Ref) |  |  | 1.00 (Ref) |  |
| A levels/AS levels | | 1.22 (1.09-1.36) | <0.001 |  | 1.22 (1.09-1.36) | <0.001 |
| O levels/GCSEs | | 1.09 (1.00-1.20) | 0.060 |  | 1.09 (0.99-1.19) | 0.066 |
| CSEs or equivalent | | 1.05 (0.86-1.28) | 0.617 |  | 1.05 (0.86-1.27) | 0.638 |
| NVQ or HND or HNC | | 1.22 (1.08-1.37) | 0.001 |  | 1.22 (1.08-1.37) | 0.001 |
| None of the above | | 1.30 (1.20-1.42) | <0.001 |  | 1.30 (1.19-1.41) | <0.001 |
| Ethnicity | |  |  |  |  |  |
| White | | 1.00 (Ref) |  |  | 1.00 (Ref) |  |
| Asian or Asian British | | 1.22 (0.78-1.89) | 0.379 |  | 1.21 (0.78-1.88) | 0.399 |
| Black or Black British | | 1.65 (1.32-2.05) | <0.001 |  | 1.62 (1.31-2.02) | <0.001 |
| Other ethnic group | | 0.92 (0.65-1.29) | 0.622 |  | 0.90 (0.64-1.26) | 0.527 |
| Physical activity | |  |  |  |  |  |
| Low | | 1.00 (Ref) |  |  | 1.00 (Ref) |  |
| Moderate | | 0.92 (0.86-0.99) | 0.027 |  | 0.92 (0.86-1.00) | 0.037 |
| High | | 0.84 (0.77-0.91) | <0.001 |  | 0.84 (0.78-0.91) | <0.001 |
| BMI (kg/m^2^) | |  |  |  |  |  |
| <25 | | 1.00 (Ref) |  |  | 1.00 (Ref) |  |
| 25 to <30 | | 0.81 (0.76-0.87) | <0.001 |  | 0.81 (0.76-0.87) | <0.001 |
| 30 to <35 | | 0.80 (0.73-0.87) | <0.001 |  | 0.80 (0.73-0.87) | <0.001 |
| ≥35 | | 0.87 (0.77-0.97) | 0.015 |  | 0.86 (0.77-0.97) | 0.010 |
| HDL | | 1.17 (1.06-1.29) | 0.001 |  | 1.12 (1.02-1.23) | 0.019 |
| LDL | | 0.99 (0.96-1.03) | 0.751 |  | 0.99 (0.96-1.03) | 0.757 |
| Health diet | | 0.99 (0.93-1.05) | 0.749 |  | 0.99 (0.94-1.06) | 0.852 |
| Consumption of  sugar-sweetened beverages | | 0.92 (0.86-0.99) | 0.025 |  | 0.93 (0.87-1.00) | 0.042 |
| Diabetes | |  |  |  |  |  |
| No | | 1.00 (Ref) |  |  | 1.00 (Ref) |  |
| Yes | | 1.73 (1.59-1.89) | <0.001 |  | 1.72 (1.58-1.87) | <0.001 |
| Cancer | |  |  |  |  |  |
| No | | 1.00 (Ref) |  |  | 1.00 (Ref) |  |
| Yes | | 0.91 (0.83-1.00) | 0.039 |  | 0.91 (0.83-0.99) | 0.037 |
| CAD | |  |  |  |  |  |
| No | | 1.00 (Ref) |  |  | 1.00 (Ref) |  |
| Yes | | 1.48 (1.35-1.62) | <0.001 |  | 1.47 (1.34-1.61) | <0.001 |
| Hypertension | |  |  |  |  |  |
| No | | 1.00 (Ref) |  |  | 1.00 (Ref) |  |
| Yes | | 1.13 (1.06-1.20) | <0.001 |  | 1.13 (1.06-1.20) | <0.001 |
| Coffee (cups/d) | Tea (cups/d) |  |  |  |  |  |
| 0 0 | | 1.00 (Ref) |  |  | 1.00 (Ref) |  |
| 0 0.5-1 | | 0.88 (0.63-1.23) | 0.454 |  | 0.89 (0.64-1.24) | 0.480 |
| 0 2-3 | | 0.84 (0.67-1.05) | 0.133 |  | 0.85 (0.68-1.06) | 0.153 |
| 0 ≥4 | | 0.69 (0.57-0.85) | <0.001 |  | 0.69 (0.57-0.85) | <0.001 |
| 0.5-1 0 | | 0.92 (0.68-1.24) | 0.588 |  | 0.93 (0.69-1.25) | 0.636 |
| 0.5-1 0.5-1 | | 0.82 (0.63-1.07) | 0.150 |  | 0.83 (0.64-1.09) | 0.177 |
| 0.5-1 2-3 | | 0.76 (0.62-0.94) | 0.011 |  | 0.77 (0.63-0.95) | 0.016 |
| 0.5-1 ≥4 | | 0.70 (0.58-0.86) | <0.001 |  | 0.71 (0.58-0.87) | <0.001 |
| 2-3 0 | | 0.82 (0.65-1.03) | 0.094 |  | 0.84 (0.66-1.05) | 0.126 |
| 2-3 0.5-1 | | 0.73 (0.57-0.92) | 0.008 |  | 0.74 (0.58-0.94) | 0.013 |
| 2-3 2-3 | | 0.72 (0.59-0.89) | 0.002 |  | 0.74 (0.60-0.90) | 0.003 |
| 2-3 ≥4 | | 0.72 (0.59-0.89) | 0.002 |  | 0.73 (0.59-0.90) | 0.003 |
| ≥4 0 | | 0.76 (0.62-0.95) | 0.015 |  | 0.77 (0.62-0.95) | 0.016 |
| ≥4 0.5-1 | | 0.79 (0.62-1.01) | 0.060 |  | 0.80 (0.63-1.02) | 0.071 |
| ≥4 2-3 | | 0.74 (0.59-0.94) | 0.012 |  | 0.75 (0.60-0.95) | 0.016 |
| ≥4 ≥4 | | 0.88 (0.70-1.09) | 0.241 |  | 0.88 (0.70-1.10) | 0.267 |
| Smoking status | |  |  |  |  |  |
| Never | |  |  |  |  |  |
| Former smokers who have  quit >5 years | | 1.07 (1.01-1.14) | 0.020 |  | 1.08 (1.01-1.16) | 0.033 |
| Former smokers who have  quit ≤5 years | |  |  |  | 1.12 (1.03-1.22) | 0.006 |
| Current <10 cigarettes/day | | 1.20 (1.09-1.32) | <0.001 |  | 1.34 (1.06-1.70) | 0.016 |
| Current 10–20 cigarettes/day | |  |  |  | 1.12 (0.94-1.32) | 0.211 |
| Current 20+ cigarettes/day | |  |  |  | 1.43 (1.21-1.69) | <0.001 |
| Alcohol status | |  |  |  |  |  |
| Never | |  |  |  |  |  |
| Former | | 1.17 (1.00-1.35) | 0.043 |  | 1.15 (0.99-1.33) | 0.072 |
| Current <7 g/day | | 0.71 (0.63-0.79) | <0.001 |  | 0.75 (0.67-0.84) | <0.001 |
| Current 7-16 g/day | |  |  |  | 0.66 (0.59-0.75) | <0.001 |
| Current >16 g/day | |  |  |  | 0.80 (0.67-1.03) | 0.068 |

^a^Multivariable model is adjusted for age, ethnicity (White, Asian or Asian British, Black or Black British, and Other ethnic group), qualification (college or university degree, A levels/AS levels or equivalent, O levels/GCSEs or equivalent, CSEs or equivalent, NVQ or HND or HNC or equivalent, other professional qualifications, or none of the above), income (less than £18,000, 18,000 to 30,999, 31,000 to 51,999, 52,000 to 100,000, and greater than 100,000), BMI (<25, 25 to <30, 30 to <35, and ≥35 kg/m^2^), physical activity (low, moderate, and high), diet pattern (health and unhealth, created by fruits, vegetables, fish, processed meats, unprocessed red meats, whole grains, refined grains), consumption of sugar-sweetened beverages, HDL, LDL, cancer, diabetes, CAD, and hypertension, smoking status (never, former, current), alcohol status (never, former, and current).

^b^Multivariable model is adjusted for age, ethnicity (White, Asian or Asian British, Black or Black British, and Other ethnic group), qualification (college or university degree, A levels/AS levels or equivalent, O levels/GCSEs or equivalent, CSEs or equivalent, NVQ or HND or HNC or equivalent, other professional qualifications, or none of the above), income (less than £18,000, 18,000 to 30,999, 31,000 to 51,999, 52,000 to 100,000, and greater than 100,000), BMI (<25, 25 to <30, 30 to <35, and ≥35 kg/m^2^), physical activity (low, moderate, and high), diet pattern (health and unhealth, created by fruits, vegetables, fish, processed meats, unprocessed red meats, whole grains, refined grains), consumption of sugar-sweetened beverages, HDL, LDL, cancer, diabetes, CAD, and hypertension, smoking status (never smokers, former smokers who have quit >5 years ago, former smokers who have quit ≤ 5 years, current smokers <10 cigarettes per day, current smokers 10–20 cigarettes per day, current smokers 20+ cigarettes per day), and alcohol status (never drinkers, former drinkers, current drinkers <7g per day, current drinkers 7-16 g per day, and current drinkers >16 g per day).
