# Supplementary material for: Consumption of coffee and tea and risk of developing stroke, dementia, and poststroke dementia: A cohort study in the UK Biobank
Source: PLoS Med. 2021 Nov 16;18(11):e1003830. doi: 10.1371/journal.pmed.1003830 (PMC8594796; doi:10.1371/journal.pmed.1003830)
Supplement: S34 Table — (DOC) [file pmed.1003830.s036.doc]

**S34 Table.** Association of coffee and tea with dementia in the UK Biobank cohort: results from competing risk regression models

| Group | | Dementia | | | |  | Alzheimer disease | | | |  | Vascular dementia | | | |
| --- | --- | --- | --- | --- | --- | --- | --- | --- | --- | --- | --- | --- | --- | --- | --- |
|  |  | Unadjusted  HR (95% CI) | *P*  value | Multi-adjusted  HR (95% CI)^a^ | *P*  value |  | Unadjusted  HR (95% CI) | *P*  value | Multi-adjusted  HR (95% CI)^a^ | *P*  value |  | Unadjusted  HR (95% CI) | *P*  value | Multi-adjusted  HR (95% CI)^a^ | *P*  value |
| Coffee (cups/d) | | | | | | | | | | | | | | | |
| 0 |  | 1.00 (Ref) |  | 1.00 (Ref) |  |  | 1.00 (Ref) |  | 1.00 (Ref) |  |  | 1.00 (Ref) |  | 1.00 (Ref) |  |
| 0.5-1 |  | 0.86 (0.80-0.93) | <0.001 | 0.97 (0.90-1.05) | 0.468 |  | 0.93 (0.82-1.05) | 0.224 | 1.04 (0.92-1.18) | 0.538 |  | 0.78 (0.67-0.91) | 0.002 | 0.91 (0.78-1.07) | 0.255 |
| 2-3 |  | 0.83 (0.77-0.90) | <0.001 | 0.96 (0.89-0.99) | 0.025 |  | 0.86 (0.76-0.97) | 0.015 | 1.01 (0.89-1.14) | 0.932 |  | 0.74 (0.64-0.87) | <0.001 | 0.89 (0.76-1.04) | 0.138 |
| ≥4 |  | 0.95 (0.88-1.04) | 0.277 | 1.04 (0.95-1.13) | 0.437 |  | 1.04 (0.91-1.19) | 0.555 | 1.17 (1.03-1.34) | 0.020 |  | 0.78 (0.66-0.93) | 0.007 | 0.84 (0.70-1.00) | 0.053 |
| Tea (cups/d) | | | | | | | | | | | | | | | |
| 0 |  | 1.00 (Ref) |  | 1.00 (Ref) |  |  | 1.00 (Ref) |  | 1.00 (Ref) |  |  | 1.00 (Ref) |  | 1.00 (Ref) |  |
| 0.5-1 |  | 0.87 (0.77-0.97) | 0.013 | 0.95 (0.85-1.06) | 0.337 |  | 0.82 (0.69-0.98) | 0.030 | 0.90 (0.75-1.07) | 0.223 |  | 0.72 (0.58-0.91) | 0.006 | 0.82 (0.65-1.03) | 0.084 |
| 2-3 |  | 0.86 (0.78-0.93) | 0.001 | 0.91 (0.83-1.00) | 0.043 |  | 0.85 (0.74-0.97) | 0.017 | 0.89 (0.77-1.02) | 0.082 |  | 0.70 (0.58-0.83) | <0.001 | 0.77 (0.65-0.92) | 0.004 |
| ≥4 |  | 0.86 (0.79-0.93) | <0.001 | 0.87 (0.80-0.95) | 0.001 |  | 0.86 (0.75-0.97) | 0.018 | 0.85 (0.75-0.97) | 0.016 |  | 0.78 (0.66-0.91) | 0.002 | 0.81 (0.69-0.96) | 0.013 |
| Coffee  (cups/d) | Tea  (cups/d) |  |  |  |  |  |  |  |  |  |  |  |  |  |  |
| 0 | 0 | 1.00 (Ref) |  | 1.00 (Ref) |  |  | 1.00 (Ref) |  | 1.00 (Ref) |  |  | 1.00 (Ref) |  | 1.00 (Ref) |  |
| 0 | 0.5-1 | 0.91 (0.65-1.27) | 0.581 | 0.87 (0.63-1.22) | 0.427 |  | 0.97 (0.59-1.60) | 0.905 | 0.97 (0.59-1.60) | 0.900 |  | 1.00 (0.57-1.77) | 0.993 | 0.92 (0.52-1.62) | 0.769 |
| 0 | 2-3 | 0.86 (0.69-1.07) | 0.169 | 0.81 (0.65-1.01) | 0.067 |  | 0.79 (0.56-1.12) | 0.181 | 0.78 (0.55-1.10) | 0.159 |  | 0.62 (0.41-0.94) | 0.024 | 0.55 (0.36-0.84) | 0.006 |
| 0 | ≥4 | 0.69 (0.57-0.85) | <0.001 | 0.77 (0.65-0.82) | <0.001 |  | 0.66 (0.48-0.90) | 0.009 | 0.67 (0.49-0.91) | 0.011 |  | 0.58 (0.41-0.84) | 0.003 | 0.53 (0.37-0.76) | 0.001 |
| 0.5-1 | 0 | 0.83 (0.62-1.12) | 0.216 | 0.90 (0.67-1.21) | 0.474 |  | 0.80 (0.50-1.27) | 0.345 | 0.91 (0.57-1.45) | 0.692 |  | 0.84 (0.50-1.42) | 0.511 | 0.88 (0.52-1.49) | 0.626 |
| 0.5-1 | 0.5-1 | 0.72 (0.55-0.93) | 0.013 | 0.80 (0.61-1.04) | 0.099 |  | 0.57 (0.37-0.88) | 0.011 | 0.67 (0.43-1.03) | 0.067 |  | 0.47 (0.28-0.79) | 0.005 | 0.52 (0.30-0.88) | 0.014 |
| 0.5-1 | 2-3 | 0.67 (0.54-0.82) | <0.001 | 0.74 (0.60-0.91) | 0.004 |  | 0.73 (0.53-1.00) | 0.051 | 0.83 (0.60-1.14) | 0.253 |  | 0.49 (0.34-0.72) | <0.001 | 0.54 (0.36-0.79) | 0.002 |
| 0.5-1 | ≥4 | 0.63 (0.51-0.76) | <0.001 | 0.68 (0.55-0.83) | <0.001 |  | 0.64 (0.47-0.88) | 0.005 | 0.72 (0.53-0.97) | 0.034 |  | 0.48 (0.33-0.68) | <0.001 | 0.50 (0.35-0.73) | <0.001 |
| 2-3 | 0 | 0.74 (0.59-0.94) | 0.012 | 0.79 (0.63-1.00) | 0.046 |  | 0.70 (0.49-1.01) | 0.055 | 0.80 (0.56-1.14) | 0.221 |  | 0.70 (0.46-1.06) | 0.092 | 0.70 (0.46-1.05) | 0.087 |
| 2-3 | 0.5-1 | 0.60 (0.47-0.76) | <0.001 | 0.70 (0.55-0.88) | 0.003 |  | 0.57 (0.40-0.83) | 0.003 | 0.71 (0.49-1.02) | 0.065 |  | 0.43 (0.27-0.67) | <0.001 | 0.48 (0.31-0.76) | 0.002 |
| 2-3 | 2-3 | 0.60 (0.49-0.74) | <0.001 | 0.69 (0.56-0.85) | <0.001 |  | 0.60 (0.43-0.82) | 0.001 | 0.71 (0.52-0.98) | 0.034 |  | 0.42 (0.29-0.61) | <0.001 | 0.47 (0.32-0.69) | <0.001 |
| 2-3 | ≥4 | 0.63 (0.51-0.77) | <0.001 | 0.69 (0.56-0.85) | <0.001 |  | 0.64 (0.47-0.88) | 0.006 | 0.73 (0.53-1.01) | 0.054 |  | 0.48 (0.33-0.70) | <0.001 | 0.51 (0.35-0.75) | <0.001 |
| ≥4 | 0 | 0.73 (0.59-0.90) | 0.004 | 0.73 (0.59-0.91) | 0.005 |  | 0.78 (0.56-1.08) | 0.132 | 0.84 (0.61-1.17) | 0.313 |  | 0.54 (0.36-0.80) | 0.002 | 0.49 (0.33-0.73) | <0.001 |
| ≥4 | 0.5-1 | 0.68 (0.54-0.87) | 0.002 | 0.76 (0.60-0.96) | 0.024 |  | 0.71 (0.49-1.02) | 0.064 | 0.85 (0.59-1.23) | 0.382 |  | 0.45 (0.28-0.71) | 0.001 | 0.47 (0.29-0.76) | 0.002 |
| ≥4 | 2-3 | 0.65 (0.52-0.82) | <0.001 | 0.71 (0.56-0.89) | 0.004 |  | 0.61 (0.43-0.88) | 0.008 | 0.72 (0.50-1.03) | 0.073 |  | 0.40 (0.25-0.63) | <0.001 | 0.41 (0.26-0.65) | <0.001 |
| ≥4 | ≥4 | 0.86 (0.80-0.93) | <0.001 | 0.84 (0.67-1.05) | 0.128 |  | 0.89 (0.64-1.26) | 0.519 | 0.99 (0.70-1.39) | 0.938 |  | 0.59 (0.39-0.90) | 0.013 | 0.57 (0.37-0.86) | 0.008 |

Abbreviations: CI, confidence interval; HR, hazard ratio; UK Biobank, United Kingdom Biobank.

^a^Multivariable model is adjusted for sex, age, ethnicity (White, Asian or Asian British, Black or Black British, and Other ethnic group), qualification (college or university degree, A levels/AS levels or equivalent, O levels/GCSEs or equivalent, CSEs or equivalent, NVQ or HND or HNC or equivalent, other professional qualifications, or none of the above), income (less than £18,000, 18,000 to 30,999, 31,000 to 51,999, 52,000 to 100,000, and greater than 100,000), BMI (<25, 25 to <30, 30 to <35, and ≥35 kg/m2), smoking status (never, former, current), alcohol status (never, former, and current), physical activity (low, moderate, and high), diet pattern (health and unhealth, created by fruits, vegetables, fish, processed meats, unprocessed red meats, whole grains, refined grains), consumption of sugar-sweetened beverages, HDL, LDL, cancer, diabetes, CAD, and hypertension, and we adjusted for coffee in tea analysis or for tea in coffee analysis.
