# Supplementary material for: Consumption of coffee and tea and risk of developing stroke, dementia, and poststroke dementia: A cohort study in the UK Biobank
Source: PLoS Med. 2021 Nov 16;18(11):e1003830. doi: 10.1371/journal.pmed.1003830 (PMC8594796; doi:10.1371/journal.pmed.1003830)
Supplement: S1 Fig — (A) Association of coffee and tea with stroke and dementia. (B) Association of coffee and tea with poststroke dementia. (DOC) [file pmed.1003830.s037.doc]

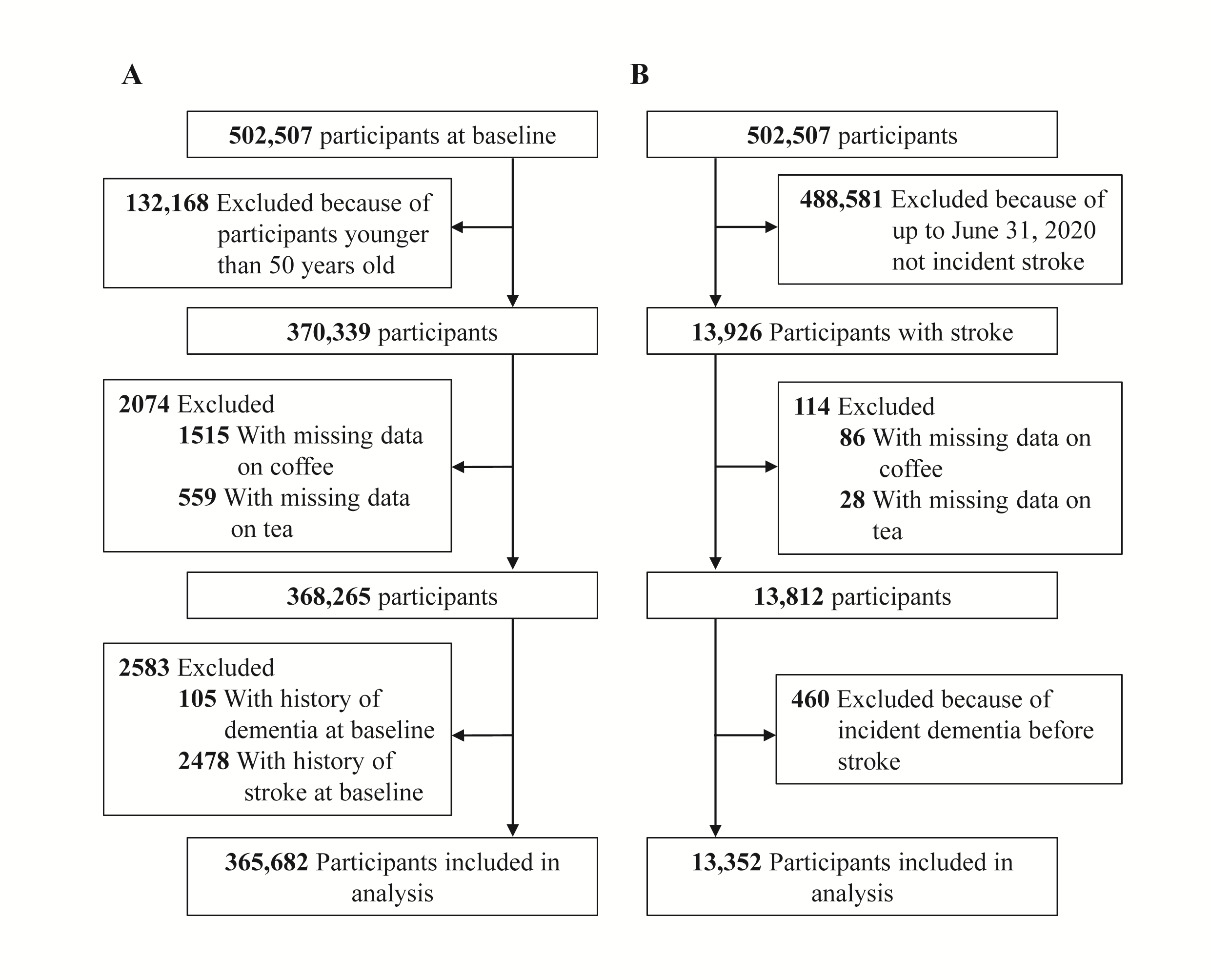


**S1 Fig.** Flowchart of participant selection. (A) Association of coffee and tea with stroke and dementia; (B) Association of coffee and tea with post-stroke dementia.
