# Supplementary figures and images for: Consumption of coffee and tea and risk of developing stroke, dementia, and poststroke dementia: A cohort study in the UK Biobank
Source: PLoS Med. 2021 Nov 16;18(11):e1003830. doi: 10.1371/journal.pmed.1003830 (PMC8594796; doi:10.1371/journal.pmed.1003830)

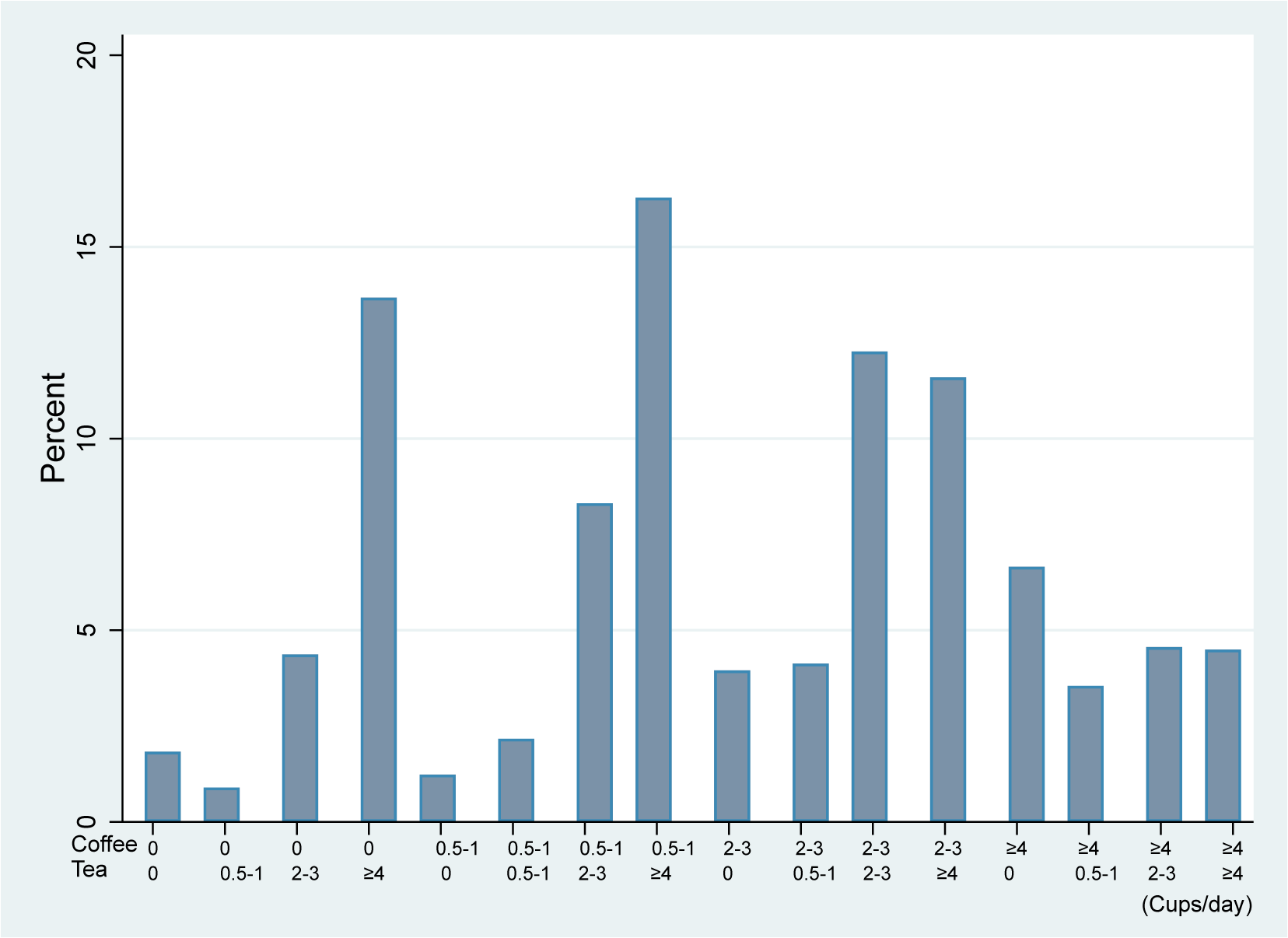


**S2 Fig.** The distribution of combination of coffee and tea intake

Supplement: S2 Fig — (DOC) [file pmed.1003830.s038.doc]
