# Supplementary material for: Consumption of coffee and tea and risk of developing stroke, dementia, and poststroke dementia: A cohort study in the UK Biobank
Source: PLoS Med. 2021 Nov 16;18(11):e1003830. doi: 10.1371/journal.pmed.1003830 (PMC8594796; doi:10.1371/journal.pmed.1003830)
Supplement: S3 Fig — (A1) Coffee and stroke. (A2) Tea and stroke. (A3) Combination of coffee and tea on stroke. (B1) Coffee and dementia. (B2) Tea and dementia. (B3) Combination of coffee and tea on dementia. (C1) Coffee and poststroke dementia. (C2) Tea and poststroke dementia. (C3) Combination of coffee and tea on poststroke dementia. The 95% CIs of the adjusted HRs are represented by the shaded area. HR, hazard ratio. (DOC) [file pmed.1003830.s039.doc]

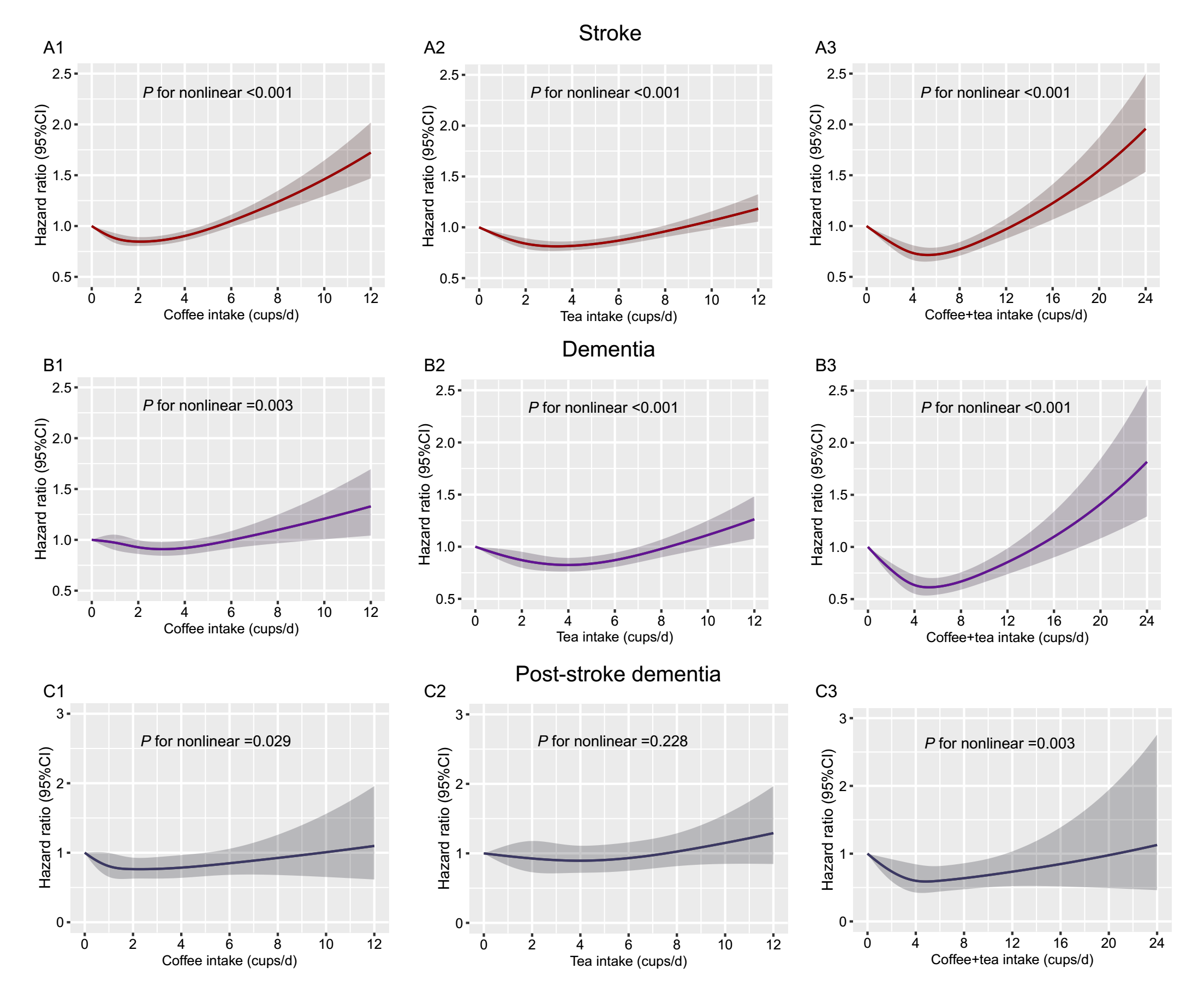


**S3 Fig.** Unadjusted Restricted Cubic Spline models for the relationship between coffee, tea, and their combination with stroke, dementia, and post-stroke dementia. (A1) Coffee and stroke; (A2) Tea and stroke; (A3) Combination of coffee and tea on stroke; (B1) Coffee and dementia; (B2) Tea and dementia; (B3) Combination of coffee and tea on dementia; (C1) Coffee and post-stroke dementia; (C2) Tea and post-stroke dementia; (C3) Combination of coffee and tea on post-stroke dementia. The 95% CIs of the adjusted hazard ratios are represented by the shaded area.
