# Supplementary material for: Consumption of coffee and tea and risk of developing stroke, dementia, and poststroke dementia: A cohort study in the UK Biobank
Source: PLoS Med. 2021 Nov 16;18(11):e1003830. doi: 10.1371/journal.pmed.1003830 (PMC8594796; doi:10.1371/journal.pmed.1003830)
Supplement: Attachment — Submitted filename: response to editors and reviews.docx [file pmed.1003830.s041.docx]

Re: Manuscript ID: PMEDICINE-D-21-00534R1 “*Association of coffee and tea with the risk of developing stroke, dementia, and post-stroke dementia*”

Dear Editor,

Thank you very much for your email regarding the revision of our manuscript “**Association of coffee and tea with the risk of developing stroke, dementia, and post-stroke dementia**” (Manuscript ID: PMEDICINE-D-21-00534R1).

We greatly thank you and the reviewers for your valuable comments. These comments are of great significance to our research. We have revised our manuscript according to your and the reviewers’ comments, and the amendments are highlighted in red in the revised manuscript. A point-to-point response to the editors’ and reviewers’ comments was attached as a separate file.

We believe that the revised manuscript is very much improved by the revisions. We appreciate you and the reviewers’ great help and efforts on this manuscript. We kindly hope the revised manuscript is acceptable for publication in *PLOS Medicine*.

Yours sincerely

Yaogang Wang, PhD (Corresponding author)

Professor

School of Public Health

Tianjin Medical University

Qixiangtai Road 22, Heping District, 300070, Tianjin, China

Phone: +86 22 8333 6636; Email: YaogangWANG@tmu.edu.cn

**Response to editors’ and reviewers’ comments**

The authors would like to thank the editors and reviewers for their constructive and positive comments on our manuscript entitled “Association of coffee and tea with the risk of developing stroke, dementia, and post-stroke dementia” (Manuscript ID: PMEDICINE-D-21-00534R1). We have made essential revisions based on the editors’ and reviewers’ comments. Point by point responses to the editors’ and reviewers’ comments are listed as below.

**Requests from the editors:**

1. Title: Please revise your title according to PLOS Medicine's style. Your title must be nondeclarative and not a question. It should begin with main concept if possible. "Effect of" should be used only if causality can be inferred, i.e., for an RCT. Please place the study design ("A randomized controlled trial," "A retrospective study," "A modelling study," etc.) in the subtitle (ie, after a colon).

Response: We appreciate the editors’ comment. Following the editors’ suggestion, we have revised our title, as below.

Title: Association of coffee and tea with the risk of developing stroke, dementia, and post-stroke dementia: A prospective cohort study

2. Interpretations and causal language: In the Abstract (and throughout manuscript, including the Author Summary): Please avoid the use of language that implies causality. For example at line 23-24 please revise: “...the separate and combined effects of coffee and tea on the risk of developing stroke, dementia, and post-stroke dementia…” and at line 41 and in the Conclusions, please avoid: “...reduced the risk…” and similar language. Throughout, we suggest referring to associations, and we recommend interpreting the results without overreaching what can be concluded from the data.
Response: We thank the editors for pointing out this issue. We have revised terms of using causal language throughout the manuscript as follows.

Page 2, lines 21-23: “little is known about the association between the combination of coffee and tea and the risk of stroke dementia, and post-stroke dementia.

Page 2, lines 25-27: “Therefore, we aimed to investigate the associations of coffee and tea separately and in combination with the risk of developing stroke and dementia.”

Page 3, lines 50-51: “Moreover, the combination of coffee and tea consumption was associated with the risk of ischemic stroke and vascular dementia.”

Page 3, lines 51-52: “Additionally, the combination of tea and coffee was associated with post-stroke dementia…”

Page 3, lines 58-60: “We found that drinking coffee and tea separately or in combination were associated with the risk of stroke and dementia. Additionally, the combination of tea and coffee was associated with the risk of post-stroke dementia.”

Page 5, lines 70-71: “Little is known about the associations between the combination of tea and coffee and the risk of stroke, dementia, and post-stroke dementia.”

Page 5, lines 80-81: “The combination of tea and coffee was associated with a lower risk of post-dementia.”

Page 5, lines 83-85: “These findings highlight the potential benefits of both coffee and tea consumption in preventing the risk of stroke, dementia, and post-stroke dementia.”

Pages 9-10, lines 154-157: “Therefore, the purpose of this study was to investigate the associations of coffee and tea separately and in combination with the risk of developing stroke, dementia, and post-stroke dementia based on data from a large population-based cohort.”

Page 19, lines 355-356: “Besides, the combination of tea and coffee was associated with the risk of post-stroke dementia…”

Page 20, lines 378-379: “…the combination of coffee and tea was associated with the risk of stroke and ischemic stroke.”

Pages 24-25, lines 483-485: “we found that: 1) the separate and combination of tea and coffee were associated with the risk of stroke, ischemic stroke, dementia, and vascular dementia;”

Page 25, lines 488-489: “the combination of coffee and tea was associated with post-stroke dementia.”

Page 26, lines 515-517: “…found that higher green tea or coffee consumption was associated with a lower risk of CVD and stroke subtypes.”

Page 27, lines 532-533: “Our study showed that there was an interaction between coffee and tea in preventing stroke and dementia.”

Page 30, lines 612-613: “Moreover, the combination of coffee and tea was associated with post-stroke dementia.”

3. Abstract: Please quantify all main results described with both 95% CIs and p values, mentioning any dependent variables that are adjusted for in the analyses.
Response: According to the editors’ suggestion, we have added both 95% CIs and *P* values in the main results, and described more details on the adjustment of dependent variables in analyses, as follows.

Page 2, lines 31-36: “We used Cox proportional hazards models to estimate hazard ratios (HRs) and 95% confidence intervals (CIs), adjusting for sex, age, ethnicity, qualification, income, body mass index (BMI), physical activity, alcohol status, smoking status, diet pattern, sugar-sweetened beverages, high density lipoprotein (HDL), low density lipoprotein (LDL), history of cancer, history of diabetes, history of cardiovascular arterial disease, and hypertension.”

Page 3, lines 45-49: “…associated with a 32% (HR, 0.68, 95% CI, 0.59-0.79; *P* <0.001) lower risk of stroke and a 28% (HR, 0.72, 95% CI, 0.59-0.89; *P* =0.002) lower risk of dementia; drinking 0.5–1 cups of coffee with ≥4 cups of tea per day was associated with a 34% (HR, 0.66, 95% CI, 0.57-0.76; *P* <0.001) lower risk of stroke and a 30% (HR, 0.70, 95% CI, 0.58-0.86; *P* <0.001) lower risk of dementia.”

Page 3, lines 53-55: “…with the lowest risk of incident post-stroke dementia at a daily consumption level of 3-6 cups of coffee and tea (HR, 0.52, 95% CI, 0.32-0.83; *P* =0.007).”

4. Abstract: In the last sentence of the Abstract Methods and Findings section, please describe the main limitation(s) of the study's methodology.
Response: Following the editors’ comment, we have added the main limitations in the abstract.

Page 3, lines 55-56: “The main limitation of this study is that coffee and tea intakes were self-report at baseline, which may not reflect long-term consumption patterns.”

5. In-text references: For in-text references within brackets, please add a space between the preceding word and the bracket.
Response: We have added a space between the preceding word and the bracket for in-text references, and have also carefully checked all other writing formats in the manuscript.

6. Methods: Line 144: Please check if this should be “tea” rather than “coffee”
Response: We apologize for the mistake. We have replaced “coffee” with “tea” in the revised manuscript (page 11, line 195).

7. Methods: Line 163: Please provide some rationale/description of the selection of covariates.
Response: We are very grateful for the editors’ comments. In this study, the selection of covariates based on: 1) demographic variables, such as sex, age, ethnicity background, education level, and income; 2) factors related to dementia or stroke reported in previous studies (*Campbell et al., Lancet, 2020; Livingston et al., Lancet, 2020*). Following the editors’ suggestion, we have added further description on the selection of covariates in the Methods section as follows:

Page 12, lines 215-218: “In the present study, the selection of covariates based on: 1) demographic variables, including sex, age, ethnicity background, education level, and income; 2) a priori knowledge of potential confounding factors associated with incident stroke and dementia [30, 31].”

8. Methods: Line 173-174: Please provide some information about the classification into “healthy” vs. “unhealthy” diet pattern (you could refer to Table S1).
Response: We thank the editors for the comments. A healthy diet was generated based on the seven commonly eaten food groups (fruits, vegetables, whole grains, refined grains, fish, unprocessed meat, and processed meat) following recommendations on dietary priorities for cardiometabolic health, which are linked to better late-life cognition and lower dementia risk (*Ilianna et al. JAMA, 2019*).

Healthy diet was based on consumption of at least 4 of 7 dietary components: 1) Fruits: ≥ 3 servings/day; 2) Vegetables: ≥ 3 servings/day; 3) Fish: ≥2 servings/week; 4) Processed meats: ≤ 1 serving/week; 5) Unprocessed red meats: ≤1.5 servings/week; 6) Whole grains: ≥ 3servings/day; 7) Refined grains: ≤1.5 servings/day. According to the editors’ suggestion, we have added more details on health dietary components in methods as follows:

Page 13, lines 229-233: “healthy diet was based on consumption of at least 4 of 7 dietary components: 1. Fruits: ≥ 3 servings/day; 2. Vegetables: ≥ 3 servings/day; 3. Fish: ≥2 servings/week; 4. Processed meats: ≤ 1 serving/week; 5. Unprocessed red meats: ≤1.5 servings/week; 6. Whole grains: ≥ 3servings/day; 7. Refined grains: ≤1.5 servings/day [32-35].”

9. Methods: Line 180: Please clarify the sentence: “Restricted Cubic Spline modeling of the relationship between coffee, tea, and their combination with stroke, dementia, and post-stroke dementia.”
Response: We thank the editors for the professional comments. In the present study, the restricted cubic spline models were used to evaluate the relationship between coffee, tea, and their combination and incident stroke and dementia. According to the editorial suggestion, we have amended the sentence as follows.

Page 14, lines 251-253: “Restricted cubic spline models were used to evaluate the relationship between coffee, tea, and their combination and incident stroke and dementia”

10. Methods: Did your study have a prospective protocol or analysis plan? Please state this (either way) early in the Methods section.

a) If a prospective analysis plan (from your funding proposal, IRB or other ethics committee submission, study protocol, or other planning document written before analyzing the data) was used in designing the study, please include the relevant prospectively written document with your revised manuscript as a Supporting Information file to be published alongside your study, and cite it in the Methods section. A legend for this file should be included at the end of your manuscript.

Response: According to the editors’ suggestion, we have included the original analysis plan in our revised manuscript as Supporting Information, and cited it in the Methods section. Meanwhile, a legend for this file was included at the end of our manuscript, as below.

Page 10, lines 165-166: “The analysis plan was drafted in April 2020 (**S1 Text**).”

Page 31, line 639: “**S1 Text**. Analysis plan”

b) If no such document exists, please make sure that the Methods section transparently describes when analyses were planned, and when/why any data-driven changes to analyses took place

Response: We thank the editors for the comments. We have included an original analysis plan and modifications for the presented analyses (**S1 Text**).

c) In either case, changes in the analysis-- including those made in response to peer review comments-- should be identified as such in the Methods section of the paper, with rationale.
Response: The presented analyses are consistent with those that were planned in the original analysis plan, with the exception of the modifications presented below.

According to the comments of reviewers, we have made several modifications to the original analysis plan: 1) we examined the associations among different coffee types and the risk of incident stroke and dementia; 2) we added stratified analysis by age, smoking status, physical activity, BMI, diet quality, and alcohol intake; 3) we performed the analysis for including participants younger than 50 years old; 4) we added competing risk models. Following the editors’ suggestion, we have added a description of changes in analyses in the Methods, as follows:

Page 11, lines 186-190: “In addition, coffee drinkers were also asked “what type of coffee do you usually drink?” and were then instructed to select one of six mutually exclusive responses, as follows: “Decaffeinated coffee (any type)”, “Instant coffee”, or “Ground coffee (include espresso and filtered coffee).” We then analyzed the associations among different coffee types and the risk of incident stroke and dementia.”

Page 15, lines 281-283: “First, we used stratification analysis to examine whether the association between tea and coffee and the risk of stroke and dementia varied by age (<65 vs. ≥65 years), sex, smoking status, alcohol status, physical activity, BMI, and diet pattern.”

Page 16, lines 287-288: “Additionally, we performed the analysis by including participants younger than 50 years old,”

Page 16, lines 294-296: “Finally, we assessed the competing risk of non-stroke or non-dementia death on the association between the combination of tea and coffee and the risks of stroke and dementia using the subdistribution method proposed by Fine and Grey [36].”

11. Methods: Please ensure that the study is reported according to the STROBE guideline, and include the completed STROBE checklist as Supporting Information. Please add the following statement, or similar, to the Methods: "This study is reported as per the Strengthening the Reporting of Observational Studies in Epidemiology (STROBE) guideline (S1 Checklist)."

The STROBE guideline can be found here: <http://www.equator-network.org/reporting-guidelines/strobe/>

When completing the checklist, please use section and paragraph numbers, rather than page numbers.
Response: Based on the editorial request, we have added the sentence “This study is reported as per the Strengthening the Reporting of Observational Studies in Epidemiology (STROBE) guideline (S1 Checklist)” in methods, and submitted the STROBE checklist as Supporting Information.

Page 10, lines 160-161: “This study is reported as per the Strengthening the Reporting of Observational Studies in Epidemiology (STROBE) guideline (**S1 Checklist**)*.*”

12. Results: Please provide 95% CIs and p values for all main analyses presented in the text.
Response: Following the editor’s suggestion, we have added 95% CIs and *P* values for all main analyses in the text throughout the results, as below.

Page 19, lines 356-358: “…with the lowest risk of incident post-stroke dementia at a daily consumption level of 3-6 cups of coffee and tea (hazard ratio about 0.50 HR, 0.52, 95% CI, 0.32-0.83; *P* =0.007).”

Page 19, lines 366-372: “Compared to that of non-coffee drinkers, HRs (95% CI) for coffee intake of 0.5–1, 2–3, and ≥ 4 cups/d were 0.90 (95% CI, 0.85-0.95; *P* <0.001), 0.88 (95% CI, 0.84-0.94; *P* <0.001), and 0.92 (95% CI, 0.86-0.98; *P* =0.009), respectively. Likewise, after multivariable adjustment for confounding factors, tea intake was associated with stroke. HRs (95% CI) of stroke for tea intake of 0.5–1, 2–3, and ≥ 4 cups/d were 0.97 (95% CI, 0.89-1.04; *P* =0.386), 0.84 (95% CI, 0.79-0.90; *P* <0.001), and 0.84 (95% CI, 0.79-0.90; *P* <0.001), respectively.”

Page 20, lines 379-383: “In multi-adjusted models, compared with those who did not drink tea and coffee, HRs of drinking 2–3 cups of coffee and 2–3 cups of tea per day were 0.68 (95% CI, 0.59–0.79; *P* <0.001) and 0.62 (95% CI, 0.51–0.75; *P* <0.001) for stroke and ischemic stroke, respectively.”

Pages 20-21, lines 397-400: “Compared with those who did not drink coffee and tea, HR (95% CI) for drinking 0.5-1 cups of coffee and ≥4 cups of tea per day was 0.70 (95% CI, 0.58-0.86; *P* <0.001), and HR (95% CI) for drinking 2-3 cups of coffee and 2-3 cups of tea per day was 0.72 (95% CI, 0.59-0.89; *P* =0.002).”

Page 22, lines 419-421: “After multivariable adjustment, participants who had a daily consumption level of 2–3 cups of coffee were associated with a lower (HR, 0.80; 95% CI, 0.64–0.99; *P* =0.044) risk of dementia than non-coffee drinkers…”

Page 22, lines 426-428: “Compared with those who did not drink coffee and tea, HRs of drinking 2–3 cups of coffee and 0 cup of tea per day were 0.38 (95% CI, 0.21–0.68; *P* =0.001) for dementia.”

13. Results: please provide both unadjusted as well as adjusted results (at least in tables if not presented in the text).
Response: We have provided both unadjusted and adjusted results of tables and figures mentioned throughout the manuscript in supplementary tables (Tables S5-S34) and figure (Figure S3), and reported the results as follows.

Pages 18, lines 349-351: “In both unadjusted (S3 Figure) and multi-adjusted models (Figure 1), the combination of coffee and tea was associated with stroke, dementia, and post-stroke dementia.”

Page 19, lines 364-365: “In unadjusted Cox models, coffee and tea intake were associated with stroke (S5 Table).”

Page 20, lines 377-379: “We found that both in unadjusted (S5 Table) and multi-adjusted models (Figure 2), the combination of coffee and tea was associated with the risk of stroke and ischemic stroke.”

Page 20, lines 389-391: “In unadjusted Cox models, intake of coffee, tea, and their combination were associated with dementia and vascular dementia, but not with Alzheimer’s disease (S6 Table).”

Pages 21-22, lines 417-419: “In unadjusted Cox models, coffee and the combination of coffee and tea were associated with dementia (S8 Table).”

Page 22, lines 434-435: “We also evaluated the associations of coffee types with stroke (S9 Table) and dementia (S10 Table).”

Additionally, Tables S11-S34 in the sensitivity analyses section provided both unadjusted and adjusted results.

14. Results: Line 244: It may be helpful to indicate within Figure 1, or in a separate table, the p values for relationships in the Restricted Cubic Spline modeling analyses.
Response: Thanks to the editors for the valuable comment. We have added *P* values for relationships in the Restricted Cubic Spline modeling analyses in Figure 1.

15. Results: Line 244-253: In the paragraph describing associations between coffee/tea consumption and stroke, please rephrase “...the combination of tea and coffee could reduce the risk of post-stroke dementia…” and similar statements, to avoid implying causality.
Response: We appreciate the editor’s comment. Following the editors’ suggestion, we have revised terms that imply causality in results and throughout the manuscript.

Page 19, lines 355-356: “Besides, the combination of tea and coffee was associated with the risk of post-stroke dementia…”

Page 20, lines 378-379: “…the combination of coffee and tea was associated with the risk of stroke and ischemic stroke.”

16. Discussion: Please present and organize the Discussion as follows: a short, clear summary of the article's findings; what the study adds to existing research and where and why the results may differ from previous research; strengths and limitations of the study; implications and next steps for research, clinical practice, and/or public policy; one-paragraph conclusion.
Response: Thanks to the editors for the professional comments. We have reorganized the Discussion according to the editors’ guidance.

Pages 24-30, lines 476-616: “In this large prospective cohort study, we found that: 1) the separate and combination of tea and coffee were associated with the risk of stroke, ischemic stroke, dementia, and vascular dementia; 2) participants who reported drinking 2–3 cups of coffee with 2–3 cups of tea per day were associated with about 30% lower risk of stroke and dementia; 3) the combination of coffee and tea seemed to correlate with lower risk of stroke and dementia compared to coffee or tea separately; 4) the combination of coffee and tea was associated with post-stroke dementia.

Many studies have investigated the relationship between separate coffee and tea consumption and stroke, but with inconsistent findings. Some reported inverse associations [37, 38], while others revealed positive or null connections [23, 28, 39-41]. Our findings supported that tea and coffee consumption related to lower risk of stroke, in accord with a review that summarized available evidence from experimental studies, prospective studies, and meta-analyses reported that tea and coffee consumption might relate to lower risk of stroke [41]. The current study also found a stronger association between the combination of tea and coffee and ischemic stroke compared to hemorrhagic stroke. Studies have reported that coffee and tea may have a different impact upon different subtypes of stroke due to the different pathogenesis and pathophysiology of the subtypes of stroke [43, 44]. A possible mechanism for this relationship is that coffee and tea are inversely associated with endothelial dysfunction, which is a major cause of ischemic stroke [45-48]. Another potential mechanism may be that coffee contains caffeine and is a rich source of antioxidants, and evidence demonstrated that coffee was inversely associated with cardiometabolic risk, including CVD, type 2 diabetes, lipids, and hypertension [19, 49, 50]. Although these explanations are biologically plausible, further studies are warranted to provide the exact underlying mechanisms of coffee and tea intake in developing ischemic stroke.

The association of a combination of coffee and tea on stroke was supported by a previous study. Kokubo et al. conducted a prospective study included 82369 Japanese, aged 45–74 years, found that higher green tea or coffee consumption was associated with a lower risk of CVD and stroke subtypes (especially in intracerebral hemorrhage) [23]. The difference is that our findings suggested that coffee and tea have a stronger impact on ischemic stroke rather than hemorrhagic stroke. The cause of this difference might be the study design, ethnic background, and classification of tea consumption. Further experimental studies are needed to verify our findings. In addition, Gelber et al. conducted the Honolulu-Asia Aging Study included 3494 men found that coffee and caffeine intake in midlife was not associated with overall dementia, Alzheimer’s disease, vascular dementia, or cognitive impairment [18], which is inconsistent with our findings. The cause of this difference might be the sample size.

Our study showed that there was an interaction between coffee and tea in preventing stroke and dementia. There are several mechanisms whereby the combination of coffee and tea may be related to stroke and dementia. First, coffee is the primary source of caffeine and contains phenolics and other bioactive compounds with potential beneficial health effects. Likewise, tea contains caffeine, catechin polyphenols, and flavonoids, which have been reported to have neuroprotective roles such as anti-oxidative stress, anti-inflammation, inhibition of amyloid-beta aggregation, and anti-apoptosis [14, 51, 52]. Coffee and tea are distinct beverages with both overlapping and different contents [21]. One potential mechanism may be related to the combined protective role of the different antioxidant and other biological contents in these 2 beverages [23]. Second, coffee and tea have a specific polyphenolic content, characterized by hydroxycinnamic acids in the former, and catechins in the latter, that have demonstrated potential benefits in ameliorating endothelial function, insulin resistance, and anti-inflammation, and have different target molecules [53], thereby, the specific polyphenolic contents of coffee and tea may play a combined protective role in the pathogenesis of stroke and dementia. Third, both coffee and tea were related to lower cardiometabolic risks, including type 2 diabetes, hypertension, and CAD [19, 49, 50]. Thus, consuming the two beverages in combination may have a joint health benefit for preventing the risk of stroke and dementia. Finally, consumption of coffee and tea may jointly modulate certain cytokine activation [54-56]. Further validation in animal experiments is warranted to examine coffee and tea’s potential joint associations on dementia.

Strengths of this study include its large sample size of UK Biobank participants, the prospective design and long-time follow up. Our present study also had several limitations. First, coffee and tea intake were self-report at baseline, which may not reflect long-term consumption patterns. Second, people who volunteer for the UK Biobank cohort tend to be, on average, more health-conscious than nonparticipants, which may lead to underestimation prevalence and incidence of stroke and dementia [58]. Third, similar to most observational studies, the bias that may be caused by unmeasured confounding factors remains, even though multiple sensitivity analyses have been carried out in the current study. Finally, our findings may only be generalizable to demographically similar cohorts, and this limitation precludes the generalization of these findings to the general population.

Among neurological disorders, stroke (42%) and dementia (10%) dominate [10]. Strokes can lead to cognitive impairment and even lead to post-stroke dementia. In addition, covert stroke and silent brain ischemia contribute to cognitive impairment and dementia [10]. Hence, preventing the risk of stroke and dementia is particularly important. Despite advances in understanding the pathophysiology of stroke and dementia, clinical treatment of stroke and dementia continues to be suboptimal. Therefore, identifying the preventable risk factors for stroke and dementia is of high priority. Our findings highlight the potential benefits of moderate coffee and tea consumption in preventing the risk of stroke and dementia. Lifestyle interventions, including promotion of healthy dietary intake (e.g., moderate coffee and tea consumption), might benefit older adults by improving stroke as well as subsequent dementia. From a public health perspective, because regular tea and coffee drinkers comprise such a large proportion of the population, and because these beverages tend to be consumed habitually throughout adult life, even small potential health benefits or risks associated with tea and coffee intake may have important public health implications. Further clinical trials on lifestyle interventions will be necessary to assess whether the observed associations are causal.

Conclusions

In conclusion, we found that drinking coffee and tea separately or in combination were associated with the risk of stroke and dementia. Moreover, the combination of coffee and tea was associated with post-stroke dementia. Our findings highlight the potential benefits of moderate coffee and tea consumption in preventing stroke and dementia. However, whether the provision of such information can improve stroke and dementia outcomes remains to be determined.”

17. Discussion: In the first paragraph and throughout, please avoid statements that imply causality, such as “...the separate and combination of tea and coffee could significantly reduce the risk of stroke…” and please address the study implications without overreaching what can be concluded from the data.
Response: We have revised the use of language that implies causality in the discussion, and have carefully checked the statements that imply causality throughout the manuscript.

Pages 24-25, lines 483-485: “we found that: 1) the separate and combination of tea and coffee were associated with the risk of stroke, ischemic stroke, dementia, and vascular dementia;”

Page 25, lines 488-489: “the combination of coffee and tea was associated with post-stroke dementia.”

Page 26, lines 515-517: “…found that higher green tea or coffee consumption was associated with a lower risk of CVD and stroke subtypes.”

Page 27, lines 532-533: “Our study showed that there was an interaction between coffee and tea in preventing stroke and dementia.”

Page 30, lines 612-613: “Moreover, the combination of coffee and tea was associated with post-stroke dementia.”

18. Discussion: Line 334: Please format the in-text citation for reference 20 within brackets.
Response: We thank the editors for the correction, and we have formatted the in-text citation for reference 20 within brackets in the revised manuscript, and carefully examined the manuscript for all such questions.

19. Discussion: Line 389-391: Please clarify what is meant by “...due to the restrictions on people’s health consciousness and regional medical level, stroke and dementia prevalence was far greater than collected” in the discussion of the study limitations.
Response: Thanks to the editors for pointing out this issue. Studies have found that people who volunteer for research studies tend to be, on average, more health-conscious than nonparticipants (*Anna et al. Am J Epidemiol, 2017; Teri A. et al. Am J Epidemiol, 2012*). Anna et al. also reported that UK Biobank participants generally live in less socioeconomically deprived areas; are less likely to be obese, to smoke, and to drink alcohol on a daily basis; and have fewer self-reported health conditions. Additionally, all-cause mortality is approximately half that of the UK population as whole, and total cancer incidence rates are approximately 10%–20% lower (*Anna et al. Am J Epidemiol,* 2017). Thus, in the current study, the incidence of stroke and dementia may be lower than the whole UK population. We have clarified the description of the limitation in the discussion as follows.

Page 29, lines 576-579: “Second, people who volunteer for the UK Biobank cohort tend to be, on average, more health-conscious than nonparticipants, which may lead to underestimation prevalence and incidence of stroke and dementia [58].”

20. Table 1: Please include definitions for all abbreviations used in the legend. Please clarify if any statistical tests were used to compare baseline characteristics.
Response: We have added abbreviations used in the legend in Table 1, as below.

Page 52, lines 1008-1011: “Abbreviations: A, Advanced; AS, Advanced Subsidiary; BMI, body mass index (calculated as weight in kilograms divided by height in meters squared); CSE, Certificate of Secondary Education; GCSE, General Certificate of Secondary Education; HDL, high density lipoprotein; HNC, Higher National Certificate; HND, Higher National Diploma; LDL, low density lipoprotein; NVQ, National Vocational Qualification; O, Ordinary; SD, standard deviation; UK Biobank, United Kingdom Biobank.”

Following the editors’ comment, we have described the details about statistical tests used to compare baseline characteristics in the methods.

Page 14, lines 247-250: “Baseline characteristics of the study population were compared across coffee or tea intake categories using analysis of variance (ANOVA) or Mann–Whitney U–test for continuous variables and chi-squared tests for categorical variables.”

21. Figure 1: If possible, please also provide the unadjusted model results.
Response: Following the editors’ suggestion, we have added unadjusted models in the supplementary figure (Figure S3), and described them in the results.

Page 18, lines 349-351: “In both unadjusted (S3 Figure) and multi-adjusted models (Figure 1), the combination of coffee and tea was associated with the risk of stroke, dementia, and post-stroke dementia.”

22. Figure 2, 3, 4: Please also provide the results from the unadjusted models.
Response: Based on the editors’ comment, we have added unadjusted model results in supplementary tables (Tables S5, S6, S8) and described them in the results.

Page 19, lines 364-365: “In unadjusted Cox models, coffee and tea intake were associated with stroke (S5 Table).”

Page 20, lines 377-379: “We found that both in unadjusted (S5 Table) and multi-adjusted models (Figure 2), the combination of coffee and tea was associated with the risk of stroke and ischemic stroke.”

Page 20, lines 389-391: “In unadjusted Cox models, intake of coffee, tea, and their combination were associated with dementia and vascular dementia, but not with Alzheimer’s disease (S6 Table).”

Pages 21-22, lines 417-419: “In unadjusted Cox models, coffee and the combination of coffee and tea were associated with post-stroke dementia (S8 Table).”

23. Table S2, S3: Please provide p values in addition to the 95% CIs, and please provide the unadjusted results.
Response: We thank the editors for the comment. We have provided p values and unadjusted results in supplementary tables (Tables S13, S14).

24. Table S4, S5, S6, S7: Please also include the unadjusted results.

Response: Following the editor’s suggestion, we have added unadjusted model results in Table S25, S26, S27, S28, respectively.

**Comments from the reviewers:**

**Reviewer #1:** This is an interesting topic that is well suited for study in the UK Biobank cohort. However, the authors did not provide strong scientific rationale for studying a potential synergistic interaction between coffee and tea with risk of stroke, dementia, and post-stroke dementia, and the conclusions, including potential implications for clinical practice, were strong given the observational nature of the study and the lack of rationale/outside evidence supporting a potential causal interaction. Greater caution is warranted in making causal inferences from an observational study.
We greatly appreciate the review’s positive comments.

Major comments for the authors consideration:
1. In the introduction it is important to state what the rationale is for undertaking an interaction analysis for coffee and tea and the outcomes. To simply state that both exposures may impact the outcome(s) is relatively uninteresting. What is the hypothesized scale (i.e., additive, multiplicative, both) and why?

Response: We thank the reviewer for the comments. Coffee and tea are among the most widely consumed beverages, both in the United Kingdom and worldwide. Coffee is closely related to tea consumption. A prospective cohort study showed that approximately 70% of participants consumed both coffee and tea (*Yuji et al. BMJ Open Diabetes Res Care, 2020*). Coffee and tea are distinct beverages with both overlapping (i.e., caffeine) and different biologically active constituents (i.e., epigallocatechin gallate, chlorogenic acid) (*Sumio et al. Molecules, 2020*). These constituents appeared to share common mechanisms, such as reactive oxygen species, on the other hand, different constituents also have different target molecules and therefore different biological effects (*Hayakawa et al. Molecules, 2020*). Genetic polymorphisms in enzymes that involved in uptake, metabolism, and excretion of tea and coffee compounds are also associated with differential biological activities of the two beverages. Additionally, two studies have found the additive or multiplicative interaction between coffee and tea on health outcomes. The Japan public health center-based study cohort reported that there was a multiplicative interaction between green tea and coffee with intracerebral hemorrhage (*P* for interaction =0.04) (*Yoshihiro et al. 2013, Stroke*). A prospective study demonstrated that there appears to be an additive interaction between green tea and coffee on mortality in Japanese patients with type 2 diabetes (*Yuji et al. BMJ Open Diabetes Res Care, 2020*). Epidemiological and clinical studies have shown the benefits of coffee and tea separately in preventing dementia. However, little is known about the association between the combination of coffee and tea and the risk of dementia.

Based on the above discussion, we hypothesized that there may be multiplicative/additive interaction between coffee and tea with incident stroke and dementia. Therefore, in the current study, we aimed to explore the association between the combination of coffee and tea, which could be the interaction of multiplication or addition, and the risk of stroke and dementia.

According to the reviewer’s suggestion, we have added further description on the reason that this study undertaking an interaction analysis for coffee and tea in the introduction section as follows. Thanks to the reviewer for the valuable comment.

Pages 8-9, lines 122-142: “Coffee is closely related to tea consumption. A prospective cohort study reported that approximately 70% of participants consumed both coffee and tea [20]. Coffee and tea are distinct beverages with both overlapping, such as caffeine, and different biologically active constituents, including epigallocatechin gallate and chlorogenic acid [21]. These constituents appeared to share common mechanisms ― reactive oxygen species, on the other hand, different constituents also have different target molecules and therefore different biological effects [21]. Furthermore, genetic polymorphisms in enzymes that involved in uptake, metabolism, and excretion of tea and coffee components were also associated with the differential biological activities of the two beverages [22]. Additionally, studies have found the interaction between green tea and coffee on health outcomes in the Japanese population [20, 23]. The Japan public health center-based study cohort reported that there was a multiplicative interaction between green tea and coffee that was associated with a lower risk of intracerebral hemorrhage [23]. A prospective study demonstrated that there appear to be an additive interaction between green tea and coffee on mortality in Japanese patients with type 2 diabetes [20]. Epidemiological and clinical studies have shown the benefits of coffee and tea separately in preventing dementia [13-18]. However, little is known about the association between the combination of coffee and tea and the risk of dementia. Therefore, we aimed to explore the association between the combination of coffee and tea, which could be multiplicative or additive interaction, and the risk of stroke and dementia.”

2. The authors' motivation for studying coffee and tea with post-stroke dementia is also not made clear in the introduction.

Response: We appreciate the review’s comment. The term post-stroke dementia refers to any dementia occurring after stroke (*Milija et al. BMC Medicine, 2017*). There is broad consensus that stroke is associated with an increased risk of subsequent dementia. A systematic review and meta-analysis reported that about 30% of people with stroke could develop dementia (*Mijajlovic et al. BMC Med, 2017*). Therefore, identifying the preventable factors for post-stroke dementia is of high priority. Previous population-based cohort studies have explored the associations between coffee or tea consumption and stroke or dementia risk. However, the associations between coffee and tea intake and incident post-stroke dementia remain unclear. Thus, in the present study, we aimed to examine the association of coffee and tea intake with incident post-stroke dementia. According to the review’s comment, we have added a description of our motivation for studying coffee and tea with post-stroke dementia in the instruction.

Page 9, lines 144-150: “Post-stroke dementia refers to any dementia occurring after stroke [24]. Post-stroke dementia poses a significant public health problem, with 30% of stroke survivors suffering from dementia [24, 25]. Thus, identifying and preventing the influencing factors of post-stroke dementia is quite important. Epidemiological studies have found inverse associations between coffee and tea and incident stroke and dementia [19, 26-28], but the associations between coffee and tea intake and incident post-stroke dementia remain unclear.”

3. While the study was limited to coffee and tea assessed at baseline, it would be important to address how these exposures change over time in the cohort and discuss how such might bias the results. The UK Biobank has serial dietary assessments on subsets of participants. Did the authors consider using this data to explore how coffee and tea drinking changed among this subset of participants? Although numbers are likely small, it would be particularly interesting if they were able to see if these behaviors changed at a similar of different rate to those who did or did not have a stroke.

Response: We completely agree with the reviewer that the study was limited to coffee and tea assessed at baseline and may not reflect long-term consumption patterns. We have added a limitation in the discussion as follows. Along the lines of the reviewer’s comment, we have started to further examine the impact of exposures changes on stroke and dementia in the next study.

Page 29, lines 573-574: “coffee and tea intakes were self-report at baseline, which may not reflect long-term consumption patterns.”

In UK Biobank, a proportion was invited several years later to repeat the assessment. For coffee and tea intake, the UK Biobank took three repeated visits: for coffee intake: 1) first repeat assessment visit (2012-13), 20335 participants; 2) second repeat assessment visit (2014+), 48640 participants; 3) third repeat assessment visit (2019+), 3781 participants. For tea: 1) first repeat assessment visit (2012-13), 20335 participants; 2) second repeat assessment visit (2014+), 48639; 3) third repeat assessment visit (2019+), 4101 participants. Based on the sample size and relatively long follow-up time, we chose the second repeat assessment visit (2014+) for analysis. 42665 participants (including 42242 non-stroke and 423 stroke participants) were included. The distributions of coffee and tea drinking change are shown in the supplementary figure (Figure S5). The percentage of participants who had never change their coffee, tea, and both intakes were 36.3%, 41.4%, and 24.3%, respectively. Additionally, the percentage of participants who had changed their coffee, tea, and both intakes within two cups per day were 86.1%, 91.4%, and 82.0%, respectively. The changes in coffee, tea, and both beverages are similar to those with and without stroke (*P* =0.955 for coffee intake change; *P* =0.576 for tea intake change; and *P* =0.759 for both the beverages intake change).

4. The nonlinear associations illustrated in Figure 1 suggest that HRs increase at higher levels of intake. Given the large size of the cohort, continuous measurement of coffee and tea, and indication of nonlinear associations, why did the authors define the highest category of intake as 4+ cups/day?

Response: We appreciate the reviewer’s comment. The associations of coffee and tea intake with stroke and dementia were nonlinear. In restricted cubic spline models, coffee intake of 2–3 cups per day was linked with the lowest hazard ratio of incident stroke and dementia, while drinking 4 or more cups of coffee per day was associated with a higher risk of dementia. Song-Yi et al. conducted the Multiethnic Cohort to assess the association of coffee consumption with total and cause-specific mortality defined the highest category of intake as ≥4 cups/day (*Song-Yi et al. Annals of internal medicine, 2017*). Additionally, two studies explored the combination of coffee and tea consumption with health outcomes, and both defined the highest category of tea intake as ≥4 cups/day (*Yoshihiro et al. 2013, Stroke; Yuji et al. BMJ Open Diabetes Res Care, 2020*). Therefore, based on both the results in terms of restricted cubic splines and literature reports, and the distribution of coffee and tea intake among the subjects, we defined the highest category of intake as ≥4 cups/day.

5. Prior UKBB analyses of coffee and/or tea have indicated that these exposures may be confounders of each other. Did the authors run analyses adjusting for coffee in tea analyses or for tea in coffee analyses to explore this possibility?

Response: We thank the reviewer for pointing out this issue. In the present study, when we run analysis, we have adjusted for coffee in tea analyses or for tea in coffee analyses. We added this description in methods and figure legends.

Page 15, lines 271-272: “…and adjusted for coffee in tea analysis or for tea in coffee analysis.”

Page 48, lines 974-975: “Figure 1. …and adjusted for coffee in tea analysis or for tea in coffee analysis.”

Page 49, lines 989-990: “Figure 2. …and adjusted for coffee in tea analysis or for tea in coffee analysis.”

Page 49, lines 1004-1005: “Figure 3. …and we adjusted for coffee in tea analysis or for tea in coffee analysis.”

6. Broadly adjusting for smoking and alcohol drinking status may not be sufficient given that these behaviors are highly correlated with coffee and tea drinking and likely associated with the outcomes of interest. Did the authors consider more detailed adjustment for smoking (e.g., intensity, time since quitting, use of other tobacco products, etc.), or alcohol (e.g., consumption level among current drinkers)? Did any of the covariates included in the models meaningfully alter HRs? If so, which ones and how was this assessed? If not, why? Finally, could CVD-related covariates (e.g., hypertension) be considered mediators? What impact if any did adjusting for these covariates have?

Response: We agree with the reviewer’s comment, and have more detail adjusted for smoking and alcohol drinking. Especially, smoking status was categorized as never smoked, former smokers who had quitted smoking >5 years, former smokers who had quitted smoking ≤ 5 years, current smokers <10 cigarettes per day, current smokers 10–20 cigarettes per day, and current smokers 20+ cigarettes per day. Alcohol drinking was categorized as never drinkers, former drinkers, current drinkers <7g per day, current drinkers 7-16 g per day, and current drinkers >16 g per day.

After detail adjusting for smoking and alcohol drinking, the HRs of coffee and tea and covariates included in the models were not significantly changed compared to broadly adjusting for smoking and alcohol drinking status. Based on the reviewer’s comment, we have reported these results in the supplementary tables (Table S31-S32), and described this in the sensitivity analysis, as follows:

Page 24, lines 466-468 & 471-473: “The results were not much altered compared with those from initial analyses when we repeated analyses: …4) with additional more detail adjustment for smoking and alcohol statuses (S31-S32 Tables);”

We included each covariate into the model individually, and found that after adjusting for sex and smoking status, the HRs of stroke in some coffee and tea combination groups were significantly changed compared to matched unadjusted models (Table S36); similarly, after adjusting for smoking status, alcohol status, and HDL separately, the HRs of dementia in some coffee and tea combination groups were significantly changed compared to matched unadjusted models (Table S37). In this study, the selection of covariates based on: 1) demographic variables, such as sex, age, ethnicity background, education level, and income; 2) factors related to dementia or stroke reported in previous studies (*Campbell et al., Lancet, 2020; Livingston et al., Lancet, 2020*). Covariates were documented including sex, age, ethnicity, education, income, BMI, smoking status, alcohol status, physical activity, sugar-sweetened beverages, history of diabetes, history of CAD, HDL, LDL, and diet pattern. In the current study, all of the above covariates were associated with stroke and dementia except for diet pattern, which was not associated with dementia (Table S38). Therefore, we selected these covariates as potential confounding factors for adjustment.

Following the review’s suggestion, we explored whether hypertension mediates the associations between coffee and tea intake and the risk of stroke and dementia. The causal steps approach proposed by Baron and Kenny were used to estimate and quantify the mediation role of hypertension on the associations (*Baron et al. J Pers Soc Psychol, 1986*). It is necessary to test 3 pathways: step 1, the association of coffee and tea with stroke and dementia; step 2, the association of coffee and tea with hypertension; step 3, the association of hypertension with stroke and dementia, controlling for coffee and tea. Bootstrapping methods were applied to estimate the 95% CI. The results showed that hypertension did not mediate the associations between coffee and tea intake and the risk of stroke ((β_indirect-coffee_= -0.00046, 95% CI: -0.0011 to 0.00021, *P* =0.203; β_indirect-tea_= 0.00059, 95% CI: -0.00010 to 0.0012, *P* =0.0914). Likewise, hypertension did not mediate the associations between coffee and tea intake and the risk of dementia (β_indirect-coffee_= -0.00020, 95% CI: -0.00039 to 0, *P* =0.0501; β_indirect-tea_= 0.00016, 95% CI: -0.0011 to 0.00037, *P* =0.109). Therefore, hypertension did not mediate the associations between coffee and tea and the risk of stroke and dementia.

After adjusting for these CVD-related covariates (CAD and hypertension), the associations between the combination of coffee and tea intake with incident stroke and dementia were not significantly changed compared to matched unadjusted models (Table S39).

7. How were disease history variables defined?

Response: Information on cardiovascular arterial disease (CAD) was derived from medical records (ICD-10 codes I20-I25). Diabetes was ascertained on the basis of medical records (ICD-10 codes E111), glycated hemoglobin ≥6.5%, and the use of anti-diabetic drugs. Hypertension was defined as systolic blood pressure (SBP) ≥140 mmHg or diastolic blood pressure (DBP) ≥90 mmHg, use of anti-hypertension agents, or medical records (ICD-10 codes I10-I13, I15). Cancer was identified through linkage to the National Health Service (NHS) Central Register (ICD-10 codes C00-C97). We have also added a description of the definition of disease history in methods as follow:

Page 13, lines 235-242: “Information on cardiovascular arterial disease (CAD) was derived from medical records (ICD-10 codes I20-I25). Diabetes was ascertained on the basis of medical records (ICD-10 codes E111), glycated hemoglobin ≥6.5%, and the use of anti-diabetic drugs. Hypertension was defined as systolic blood pressure (SBP) ≥140 mmHg or diastolic blood pressure (DBP) ≥90 mmHg, use of anti-hypertension agents, or medical records (ICD-10 codes I10-I13, I15). Cancer was identified through linkage to the National Health Service (NHS) Central Register (ICD-10 codes C00-C97).”

8. Authors should refrain from using causal language throughout the paper (e.g., lines 74-75, line 383).

Response: We have revised all terms of causal language throughout the manuscript.

Page 2, lines 21-23: “little is known about the association between the combination of coffee and tea and the risk of stroke dementia, and post-stroke dementia.

Page 2, lines 25-27: “Therefore, we aimed to investigate the associations of coffee and tea separately and in combination with the risk of developing stroke and dementia.”

Page 3, lines 50-51: “Moreover, the combination of coffee and tea consumption was associated with the risk of ischemic stroke and vascular dementia.”

Page 3, lines 51-52: “Additionally, the combination of tea and coffee was associated with post-stroke dementia…”

Page 3, lines 58-60: “We found that drinking coffee and tea separately or in combination were associated with the risk of stroke and dementia. Additionally, the combination of tea and coffee was associated with the risk of post-stroke dementia.”

Page 5, lines 70-71: “Little is known about the associations between the combination of tea and coffee and the risk of stroke, dementia, and post-stroke dementia.”

Page 5, lines 80-81: “The combination of tea and coffee was associated with a lower risk of post-dementia.”

Page 5, lines 83-85: “These findings highlight the potential benefits of both coffee and tea consumption in preventing the risk of stroke, dementia, and post-stroke dementia.”

Pages 9-10, lines 154-157: “Therefore, the purpose of this study was to investigate the associations of coffee and tea separately and in combination with the risk of developing stroke, dementia, and post-stroke dementia based on data from a large population-based cohort.”

Page 19, lines 355-356: “Besides, the combination of tea and coffee was associated with the risk of post-stroke dementia…”

Page 20, lines 378-379: “…the combination of coffee and tea was associated with the risk of stroke and ischemic stroke.”

Pages 24-25, lines 483-485: “we found that: 1) the separate and combination of tea and coffee were associated with the risk of stroke, ischemic stroke, dementia, and vascular dementia;”

Page 25, lines 488-489: “the combination of coffee and tea was associated with post-stroke dementia.”

Page 26, lines 515-517: “…found that higher green tea or coffee consumption was associated with a lower risk of CVD and stroke subtypes.”

Page 27, lines 532-533: “Our study showed that there was an interaction between coffee and tea in preventing stroke and dementia.”

Page 30, lines 612-613: “Moreover, the combination of coffee and tea was associated with post-stroke dementia.”

9. In the discussion, the argument for why there could be a causal interaction is weak, while little may be known about the combined effects of coffee and tea drinking, the biological plausibility for a joint effect should be clearly laid out. Currently, it reads as though coffee and tea contain the same beneficial compounds (e.g., caffeine, polyphenols, etc.) and so more of either or both is beneficial. Thus, the case for prescriptions, citing the best combination of coffee and tea, seems unfounded and potentially misleading.

Response: We completely understand the reviewer’s concern. In the current study, our results suggested that the combination of coffee and tea seemed to correlate with a lower risk of stroke and dementia compared to coffee or tea separately, and there were multiplicative interactions between coffee and tea on stroke and dementia.

There are several mechanisms whereby the combination of coffee and tea may be related to stroke and dementia. Coffee is a complex beverage containing hundreds of biologically active compounds, including caffeine, chlorogenic acid, and caffeic acid (*Masood et al. Crit Rev Food Sci Nutr, 2011*). Tea containing caffeine, catechin polyphenols, and flavonoids has been reported to play neuroprotective roles. Indeed, coffee and tea contain the same beneficial compounds, such as caffeine, while they also contain some different compounds, such as epigallocatechin gallate and chlorogenic acid (*Braidy et al. Curr Top Med Chem, 2016*). One potential mechanism may be related to the combined protective role of the different antioxidant and other biological contents in these 2 beverages (*Kokubo et al. Stroke, 2013*). Moreover, coffee and tea have a specific polyphenolic content, characterized by hydroxycinnamic acids in the former, and catechins in the latter, that have demonstrated potential beneficial effects in ameliorating endothelial function, insulin resistance, and anti-inflammation, and have different target molecules (*Marventano et al. Clin Nutr, 2016*). Thus, consuming the two beverages in combination may have a cumulative health benefit for preventing the pathogenesis of stroke and dementia. Furthermore, both coffee and tea were related to lower cardiometabolic risks, including type 2 diabetes, hypertension, and CAD, which may play a combined protective role on the risk of stroke and dementia. Additionally, consumption of coffee and tea may jointly modulate certain cytokine activation, including interleukin 6 and insulin-like growth factor 1 (*Dong et al. Am J Clin Nutr, 2019*).

Based on the review’s suggestion, we have added the potential biological plausibility for the combination of coffee and tea attribute to stroke and dementia as below.

Pages 27-28, lines 539-554: “Coffee and tea are distinct beverages with both overlapping and different contents [21]. One potential mechanism may be related to the combined protective role of the different antioxidant and other biological contents in these 2 beverages [23]. Second, coffee and tea have a specific polyphenolic content, characterized by hydroxycinnamic acids in the former, and catechins in the latter, that have demonstrated potential benefits in ameliorating endothelial function, insulin resistance, and anti-inflammation, and have different target molecules [53], thereby, the specific polyphenolic contents of coffee and tea may play a combined protective role in the pathogenesis of stroke and dementia. Third, both coffee and tea were related to lower cardiometabolic risks, including type 2 diabetes, hypertension, and CAD [19, 49, 50]. Thus, consuming the two beverages in combination may have a joint health benefit for preventing the risk of stroke and dementia. Finally, consumption of coffee and tea may jointly modulate certain cytokine activation [54-56].”

10. Line 392, although some types of coffee preparations were not assessed in the cohort the two main types of coffee (i.e., instant and ground) were. Did the authors consider associations with instant versus brewed coffee? What about caffeinated versus decaffeinated?
Response: In UK Biobank, types of coffee were assessed at baseline using a touchscreen questionnaire. Coffee drinkers were asked “*what type of coffee do you usually drink?*” and were then instructed to select one of six mutually exclusive responses, as follows: “*Decaffeinated coffee (any type)*”, “*Instant coffee*”, or “*Ground coffee (include espresso and filtered coffee)*.” According to the reviewer’s suggestion, we have further examined the associations among different coffee types and the risk of incident stroke and dementia.

The results demonstrated that in multi-adjusted Cox regression models, compared to instant coffee, ground coffee was not associated with stroke (HR, 0.98; 95% CI, 0.93-1.04; *P* =0.619) and its subtypes. Compared to decaffeinated coffee, instant coffee was not associated with stroke (HR, 0.95; 95% CI, 0.90-1.01; *P* =0.074) and its subtypes, while ground coffee was associated with a lower risk of stroke (HR, 0.90, 95% CI, 0.84-0.97; *P* =0.006) and ischemic stroke (HR, 0.90, 95% CI, 0.82-1.00; *P* =0.045). For dementia, in multi-adjusted Cox regression models, compared to instant coffee, ground coffee was associated with a lower risk of dementia (HR, 0.83; 95% CI, 0.77-0.89; *P* <0.001), Alzheimer disease (HR, 0.77; 95% CI, 0.69-0.87; *P* <0.001), and vascular dementia (hazard ratio, 0.82; 95% CI, 0.70-0.96; *P* =0.012). Compared to decaffeinated coffee, instant coffee was associated with dementia (HR, 0.85; 95% CI, 0.79-0.92; *P* <0.001), Alzheimer disease (HR, 0.81; 95% CI, 0.72-0.91; *P* <0.001), and vascular dementia (HR, 0.84; 95% CI, 0.72-0.99; *P* =0.036); ground coffee was associated with dementia (HR, 0.74; 95% CI, 0.66-0.82; *P* <0.001), Alzheimer disease (HR, 0.67; 95% CI, 0.57-0.78; P <0.001), and vascular dementia (HR, 0.74; 95% CI, 0.59-0.92; *P* =0.008).

Based on the reviewer’s suggestion, we have added information on coffee types in the methods, as follows:

Page 11, lines 186-190: “In addition, coffee drinkers were also asked “what type of coffee do you usually drink?” and were then instructed to select one of six mutually exclusive responses, as follows: “Decaffeinated coffee (any type)”, “Instant coffee”, or “Ground coffee (include espresso and filtered coffee).” We then analyzed the associations among different coffee types and the risk of incident stroke and dementia.”

Furthermore, we added the association among different coffee types and incident stroke and dementia in the supplementary table (Tables S9-S10) and described them in the results.

Pages 22-23, lines 434-452: “We also evaluated the associations of coffee types with stroke (S9 Table) and dementia (S10 Table). Among coffee drinkers, 160741 (44.0%), 63363 (17.3%), and 57397 (15.7%) participants reported drinking instant, ground, and decaffeinated coffee, respectively. In unadjusted and multi-adjusted Cox regression models, compared to instant coffee, ground coffee was not associated with stroke (HR, 0.98; 95% CI, 0.93-1.04; *P* =0.619) and its subtypes. Compared to decaffeinated coffee, instant coffee was not associated with stroke (HR, 0.95; 95% CI, 0.90-1.01; *P* =0.074) and its subtypes, while ground coffee was associated with a lower risk of stroke (HR, 0.90, 95% CI, 0.84-0.97; *P* =0.006) and ischemic stroke (HR, 0.90, 95% CI, 0.82-1.00; *P* =0.045). For dementia, in multi-adjusted Cox regression models, compared to instant coffee, ground coffee was associated with a lower risk of dementia (HR, 0.83; 95% CI, 0.77-0.89; *P* <0.001), Alzheimer disease (HR, 0.77; 95% CI, 0.69-0.87; *P* <0.001), and vascular dementia (hazard ratio, 0.82; 95% CI, 0.70-0.96; *P* =0.012). Compared to decaffeinated coffee, instant coffee was associated with dementia (HR, 0.85; 95% CI, 0.79-0.92; *P* <0.001), Alzheimer disease (HR, 0.81; 95% CI, 0.72-0.91; *P* <0.001), and vascular dementia (HR, 0.84; 95% CI, 0.72-0.99; *P* =0.036); ground coffee was associated with dementia (HR, 0.74; 95% CI, 0.66-0.82; *P* <0.001), Alzheimer disease (HR, 0.67; 95% CI, 0.57-0.78; P <0.001), and vascular dementia (HR, 0.74; 95% CI, 0.59-0.92; *P* =0.008).”

Minor comments:
1. The paper is generally very well written with the exception of the discussion, which is confusing and would benefit from proof-reading (some examples below).

Response: We appreciate the reviewer’s comment. We benefited from your comments and suggestions and have carefully checked the language in the discussion.

2. Citation for line 95-96 "In principle, 90% of strokes and 35% of dementia have been estimated to be preventable."

Response: Following the reviewer’s suggestion, we have added citations for the sentence.

Page 7, lines 108-109: “In principle, 90% of strokes and 35% of dementia have been estimated to be preventable [7-10].”

3. The following sentence should be revised so that it doesn't start with a number or use the phrase "were daily drinking":  "59,558 (16.29%) participants reported drinking 0.5-1 cup of coffee and ≥4 cups of tea per day.."

Response: We agree with the reviewer’s comment, and we have amended the sentences in results as below.

Page 17, lines 323-328: “Of the 365,682 participants, 59,558 (16.29%) participants reported drinking 0.5-1 cup of coffee and ≥4 cups of tea per day, accounting for the largest proportion; followed by 50,015 (13.68%) participants reported drinking 0 cup of coffee and ≥4 cups of tea per day; besides, 44,868 (12.27%) participants reported drinking 0 cup of coffee and ≥4 cups of tea per day.”

4. Line 334- Fix citation to match formatting throughout.

Response: We thank the reviewer for pointing out this issue, we have reformatted the citation in the revised manuscript.

5. "While Qian et al.[24] reported that coffee consumption is not causally associated with the risk of stroke, which is different from ours results." The results from the current study should also not be interpreted as causal, please rephrase.

Response: We thank the reviewer for the comment. Given the observational epidemiology nature of the present study and the lack of rationale evidence supporting a potential causal interaction, it is difficult to make causal inferences from an observational study. Therefore, it is not appropriate to compare with Qian’s research, so we have deleted this sentence in the revised manuscript. Additionally, we have revised the use of language that implies causality throughout the manuscript.

6. Line 337- and coffee intake "groupings" is probably critical

Response: We thank the reviewer for the corrections. We have replaced “grouping” with “groupings” in the revised manuscript (page 25, line 499).

7. Line 339- compared to hemorrhagic stroke

Response: We appreciate the reviewer’s careful review, and we have replaced “than” with “compared to” in the revised manuscript (page 25, line 501).

8. The association of combination of coffee and tea on stroke was supporting supported by a previous study

Response: We have replaced “supporting” with “supported” in the revised manuscript (page 26, line 514).

9. Line 349- that included "82,369" Japanese, age aged

Response: We have deleted the word “age” in the revised manuscript (page 26, line 515).

10. Line 351- The "difference" is that our

Response: We thank the reviewer again for the corrections, and we have replaced “different” with “difference” in the revised manuscript (page 26, line 517).

11. Line 357-358 reword, unclear- While this study mainly focuses on caffeine intake, but not different combinations of coffee and tea intake.

Response: We thank the reviewer for the comment. Rebecca et al. conducted the Honolulu-Asia Aging Study to examined associations of coffee and caffeine intake in midlife with risk of dementia, its neuropathologic correlates, and cognitive impairment among 3494 men (*Rebecca et al. J Alzheimers Dis, 2011*). Following the reviewer's suggestion, we have amended the sentence as follows:

Page 26, lines 524-528: “In addition, Gelber et al. conducted the Honolulu-Asia Aging Study included 3494 men found that coffee and caffeine intake in midlife was not associated with overall dementia, Alzheimer’s disease, vascular dementia, or cognitive impairment [18], which is inconsistent with our findings. The cause of this difference might be the sample size.”

12. Line 360- Not clear "an interaction effect for each other?"

Response: Thanks for the comment. We have revised it as below.

Page 27, lines 532-533: “Our study showed that there was an interaction between coffee and tea in preventing stroke and dementia.”

13. Line 369- Needs citation "Consumption of coffee and tea may jointly modulate certain cytokine activation."

Response: Following the reviewer’s suggestion, we have added citations for the sentence as below.

Page 28, lines 553-554: “Consumption of coffee and tea may jointly modulate certain cytokine activation [54-56].”

14. Line 373- Needs citation- Among neurological disorders, stroke (42%) and dementia (10%) dominate.

Response: Following the reviewer’s suggestion, we have added a citation for the sentence.

Page 29, line 589: “Among neurological disorders, stroke (42%) and dementia (10%) dominate [10].”

**Reviewer #2:** This is an interesting and useful study on the association of coffee and tea with the risk of developing stroke, dementia, and post-stroke dementia. However, there are a few major issues needing attention.

We greatly appreciate the reviewer’s encouraging comment.

1) Competing risk. In Cox models, when the outcome is an incident disease (stroke, demintia and etc) other than all-cause mortality, models taking into account of competing risk should be used. However, competing risk was not considered at all in the paper which is not adequate.
Response: As the reviewer pointed out, we have provided competing risk models in the supplementary table (Tables S33-S34), and found that the results were not significantly changed. We have described these as sensitivity analyses in the results.

Page 24, lines 466-468 & 473: “The results were not much altered compared with those from initial analyses when we repeated analyses: …4) using a competing risk regression model (S33-S34Tables).”

2) The dose-response relationships in Figure 1 is simply univariate relationship which's not multiple-adjusted by confounders so could be misleading therefore can't interpret too much.

Response: We appreciate the reviewer’s careful review, and we apologize that we have not clearly described the adjusted confounders in Figure 1. In the current study, restricted cubic spline models were used to evaluate the relationship between coffee, tea, and their combination with stroke, dementia, and post-stroke dementia. Spline models were adjusted for sex, age, ethnicity, education, income, body mass index, physical activity, alcohol status, smoking status, diet pattern, sugar-sweetened beverages, high density lipoprotein, low density lipoprotein, history of cancer, history of diabetes, history of cardiovascular arterial disease, and hypertension, further, we adjusted for coffee in tea analysis or tea in coffee analysis. We have added a description of the confounding factors for the adjustment of the restricted cubic spline models in the methods, and added univariate restricted cubic spline models in Figure S3 as a supplementary figure, and also described this in the results.

Page 14, lines 251-259: “Restricted cubic spline models were used to evaluate the relationship between coffee, tea, and their combination and incident stroke and dementia, with four knots at the 25th, 50th, 75th, and 95th centiles. In the spline models, we adjusted for sex, age, ethnicity, education, income, body mass index, physical activity, alcohol status, smoking status, diet pattern, sugar-sweetened beverages, high density lipoprotein, low density lipoprotein, history of cancer, history of diabetes, history of cardiovascular arterial disease, and hypertension, further, we adjusted for coffee in tea analysis or tea in coffee analysis.”

Page 18, lines 349-351: “In both unadjusted (S3 Figure) and multi-adjusted models (Figure 1), the combination of coffee and tea was associated with stroke, dementia, and post-stroke dementia.”

3) The data on coffee and tea intake at baseline doesn't have information as how long they have been drinking and whether they are frequent drinkers of coffee or tea. This will have a big impact of the validity of causal relationship between coffee/tea and stroke/dementia.
Response: We agree with the reviewer that coffee and tea intakes were assessed at baseline and may not reflect long-term consumption patterns. Potential changes in coffee and tea intake after the baseline examination may have influenced our risk estimates. We have added a limitation in the discussion as follows.

Page 29, lines 573-574: “coffee and tea intakes were self-report at baseline, which may not reflect long-term consumption patterns.”

Furthermore, the UK Biobank took some of the participants for three repeated visits: for coffee intake: Initial assessment visit (2006-2010), mean (SD, cups/day) = 2.13; First repeat assessment visit (2012-13), 20335 participants, mean =2.19; Second repeat assessment visit (2014+), 48640 participants, mean =2.15; Third repeat assessment visit (2019+), 3781 participants, mean =2.19. For tea: Initial assessment visit (2006-2010), mean (cups/day) =3.52; First repeat assessment visit (2012-13), 20335 participants, mean =3.61; Second repeat assessment visit (2014+), 48639 participants, mean =3.40; Third repeat assessment visit (2019+), 4101 participants, mean =3.28. Based on the sample size and relatively long follow-up time, we chose the second repeat assessment visit (2014+) to assess changes in coffee and tea intake. The distributions of coffee and tea drinking change are shown in Figure S5 as a supplementary figure. The percentage of participants who had never change their coffee, tea, and both intakes were 36.3%, 41.4%, and 24.3%, respectively. Additionally, the percentage of participants who had changed their coffee, tea, and both intakes within two cups per day were 86.1%, 91.4%, and 82.0%, respectively. Along the lines of the reviewer’s comment, we have started to further examine the impact of exposures changes on stroke and dementia in next study.

4) Association is not causation. So far what authors found is association between coffee/tea and stroke/dementia, but strong causal relationship was concluded throughout the paper, for example in abstract, "We found that drinking coffee and tea separately or in combination could reduce the risk of stroke and dementia. Additionally, the combination of tea and coffee could reduce the risk of post-stroke dementia". This's a very strong claim which is not adequate. Authors should really tone down the claims.
Response: We agree with the reviewer that a strong causal relationship was concluded throughout the manuscript. Following the reviewer’s suggestion, we have tone down the claims, and amended the sentence that implies causality throughout the paper, as below.

Page 3, lines 58-60: “We found that drinking coffee and tea separately or in combination were associated with the risk of stroke and dementia. Additionally, the combination of tea and coffee was associated with the risk of post-stroke dementia.”

Page 2, lines 21-23: “little is known about the association between the combination of coffee and tea and the risk of stroke dementia, and post-stroke dementia.

Page 2, lines 25-27: “Therefore, we aimed to investigate the associations of coffee and tea separately and in combination with the risk of developing stroke and dementia.”

Page 3, lines 50-51: “Moreover, the combination of coffee and tea consumption was associated with the risk of ischemic stroke and vascular dementia.”

Page 3, lines 51-52: “Additionally, the combination of tea and coffee was associated with post-stroke dementia…”

Page 5, lines 70-71: “Little is known about the associations between the combination of tea and coffee and the risk of stroke, dementia, and post-stroke dementia.”

Page 5, lines 80-81: “The combination of tea and coffee was associated with a lower risk of post-dementia.”

Page 5, lines 83-85: “These findings highlight the potential benefits of both coffee and tea consumption in preventing the risk of stroke, dementia, and post-stroke dementia.”

Pages 9-10, lines 154-157: “Therefore, the purpose of this study was to investigate the associations of coffee and tea separately and in combination with the risk of developing stroke, dementia, and post-stroke dementia based on data from a large population-based cohort.”

Page 19, lines 355-356: “Besides, the combination of tea and coffee was associated with the risk of post-stroke dementia…”

Page 20, lines 378-379: “…the combination of coffee and tea was associated with the risk of stroke and ischemic stroke.”

Pages 24-25, lines 483-485: “we found that: 1) the separate and combination of tea and coffee were associated with the risk of stroke, ischemic stroke, dementia, and vascular dementia;”

Page 25, lines 488-489: “the combination of coffee and tea was associated with post-stroke dementia.”

Page 26, lines 515-517: “…found that higher green tea or coffee consumption was associated with a lower risk of CVD and stroke subtypes.”

Page 27, lines 532-533: “Our study showed that there was an interaction between coffee and tea in preventing stroke and dementia.”

Page 30, lines 612-613: “Moreover, the combination of coffee and tea was associated with post-stroke dementia.”

5) The key message was still not clearly expressed. We know the impact of coffee or tea separately on stroke and dementia, but what exactly is the combined impact? what's the difference compared to individual effect? larger, smaller or the same? and what's possible explanations of findings for the combined effect?

Response: We completely understand the reviewer’s comments. In the current study, our results suggested that the combination of coffee and tea seemed to correlated with a lower risk of stroke and dementia compared to coffee or tea separately, and there were multiplicative interactions between coffee and tea on stroke and dementia (*P* for interaction <0.001 for stroke and *P* for interaction =0.0127 for dementia). Such as, for dementia, compared with those who did not drink coffee and tea, HR for drinking 2-3 cups of coffee and 0 cups of tea per day was 0.82 (95% CI, 0.65-1.02; *P* =0.094), and HR for drinking 0 cups of coffee and 2-3 cups of tea per day was 0.84 (95% CI, 0.67-1.05; *P* =0.133), and HR (95% CI) for drinking 2-3 cups of coffee and 2-3 cups of tea per day was 0.72 (95% CI, 0.59-0.89; *P* =0.002).

Furthermore, we evaluate the HRs of those who drank both coffee and tea compared to those who only drank either coffee or tea. For stroke, after adjustment for confounders, compared with participants who only drank either coffee or tea, HR for those who drank both coffee and tea was 0.89 (95% CI, 0.86-0.93; *P* <0.001); for dementia, compared with participants who only drank either coffee or tea, HR for those who drank both coffee and tea was 0.92 (95% CI, 0.87-0.98; *P* =0.0011). These results also suggested that the combination of coffee and tea seemed to correlate with a lower risk of stroke and dementia compared to coffee or tea separately. Following the review’s comments, we have added these results in a supplementary table (Table S7), and described them in the results and discussion, as follows:

Page 21, lines 405-411: “Additionally, we evaluate the HRs of participants who drank both coffee and tea compared to those who only drank either coffee or tea (S7 Table). After adjustment for confounders, compared with participants who only drank either coffee or tea, those who drank both coffee and tea was associated with lower risk of stroke (HR, 0.89; 95% CI, 0.86-0.93; *P* <0.001), ischemic stroke (HR, 0.89; 95% CI, 0.84-0.94; *P* <0.001), dementia (HR, 0.92; 95% CI, 0.87-0.98; *P* =0.001), and vascular dementia (HR, 0.82; 95% CI, 0.72-0.92; *P* <0.001).”

Page 25, lines 487-488: “the combination of coffee and tea seemed to correlate with lower risk of stroke and dementia compared to coffee or tea separately;”

There are several mechanisms whereby the combination of coffee and tea may be related to stroke and dementia: 1) coffee is a complex beverage containing hundreds of biologically active compounds, including caffeine, chlorogenic acid, and caffeic acid (*Masood et al. Crit Rev Food Sci Nutr, 2011*). Tea containing caffeine, catechin polyphenols, and flavonoids has been reported to play neuroprotective roles (*Braidy et al. Curr Top Med Chem. 2016*). One potential mechanism may be related to the combined protective role of the different antioxidant and other biological contents in these 2 beverages (*Kokubo et al. Stroke, 2013*). 2) coffee and tea have a specific polyphenolic content, characterized by hydroxycinnamic acids in the former, and catechins in the latter, that have demonstrated potential beneficial effects in ameliorating endothelial function, insulin resistance, and anti-inflammation, and have different target molecules (*Marventano et al. Clin Nutr, 2016*). Thus, consuming the two beverages in combination may have a cumulative health benefit for preventing the pathogenesis of stroke and dementia. 3) both coffee and tea were related to lower cardiometabolic risks, including type 2 diabetes, hypertension, and CAD, which may play a combined protective role on the risk of stroke and dementia. 4) consumption of coffee and tea may jointly modulate certain cytokine activation, including interleukin 6 and insulin-like growth factor 1 (*Dong et al. Am J Clin Nutr, 2019*).

Based on the review’s suggestion, we have added the potential mechanisms responsible for the combination of coffee and tea attribute to stroke and dementia as below.

Pages 27-28, lines 539-554: “Coffee and tea are distinct beverages with both overlapping and different contents [21]. One potential mechanism may be related to the combined protective role of the different antioxidant and other biological contents in these 2 beverages [23]. Second, coffee and tea have a specific polyphenolic content, characterized by hydroxycinnamic acids in the former, and catechins in the latter, that have demonstrated potential benefits in ameliorating endothelial function, insulin resistance, and anti-inflammation, and have different target molecules [53], thereby, the specific polyphenolic contents of coffee and tea may play a combined protective role in the pathogenesis of stroke and dementia. Third, both coffee and tea were related to lower cardiometabolic risks, including type 2 diabetes, hypertension, and CAD [19, 49, 50]. Thus, consuming the two beverages in combination may have a joint health benefit for preventing the risk of stroke and dementia. Finally, consumption of coffee and tea may jointly modulate certain cytokine activation [54-56].”

**Reviewer #3:** In this study, Zhang et al examined associations of coffee and tea with the risk of developing stroke, dementia, and post-stroke dementia among 365,682 participants in the UK Biobank. The authors found that drinking coffee and tea separately or in combination was associated with lower risks of stroke and dementia, particularly vascular dementia. This is a highly interesting study, especially the finding that intakes of coffee and tea were associated with lower risk of vascular dementia, but not Alzheimer disease, which provides further evidence that coffee intake is related to cardiometabolic health.
We are grateful to the reviewer for the encouraging comment.

Main questions
1)     Figure 4 is unnecessary. Based on figures 1-3, coffee or tea is likely to be related to risks of dementia through stroke. If the analysis is restricted to stroke participants, it is expected that coffee/tea is not related to dementia or vascular dementia due to block of the mediator (stroke). I do not think figure 4 is needed. Additionally, it is strange that coffee or tea separately is not associated with dementia, but jointly associated with a lower risk. Might need to double check the data analysis procedures.
Response: We agree with the reviewer that a combination of coffee and tea is likely to be related to the risk of dementia through stroke. We further investigated whether the association between the combination of coffee and tea and incident dementia was mediated entirely or partly by stroke. We explored the associations of coffee and tea with incident stroke without incident dementia, incident dementia without incident stroke, and incident both stroke and dementia. We found that combination of coffee and tea was associated with incident stroke without incident dementia, incident dementia without incident stroke, and incident both outcomes. Our results of the combination of coffee and tea was associated with incident dementia without incident stroke suggested that stroke may partly account for the association between the combination of coffee and tea and the risk of dementia. We have added these results in a supplementary table (Table S35).

Furthermore, based on the reviewer’s suggestion, we have checked the data analysis procedures, and found that there were mistakes in data analysis procedures about Alzheimer disease and vascular dementia outcomes. We apologize for the mistake and have corrected these, and have also carefully checked all other data analysis procedures throughout the manuscript. Following the reviewer’s comment, we have deleted figure 4 and added it in a supplementary figure (Figure S4) in our revised manuscript.

2)     Instead of figure 4, would it be possible to look at coffee and tea intake after development of stroke with development of dementia? Line 382. "Our findings highlight the potential benefits of coffee and tea consumption to reduce the risk of dementia in participants with stroke". This statement is not convincingly supported by the data, unless you can show an inverse association for coffee/tea intake after stroke incidence. However, I think this study showed potential benefits of coffee and tea in preventing vascular dementia.
Response: We are grateful to the reviewer for the comment. We agree with the reviewer that this study showed the potential benefits of coffee and tea in preventing vascular dementia. In UK Biobank, coffee and tea intake were assessed at baseline and annual follow-up data were lacking. Therefore, due to the limitation of the data, we can’t look at coffee and tea intake after the development of stroke with development of dementia. Following the reviewer’s suggestion, we have deleted the sentence “Our findings highlight the potential benefits of coffee and tea consumption to reduce the risk of dementia in participants with stroke”.

3)     To show the robustness of the findings, the author can conduct stratified analysis by smoking status, which is related to coffee intake, as well as age, sex, physical activity, BMI, diet quality, and alcohol intake.
Response: Following the reviewer’s suggestion, we have conducted stratified analysis by age, sex, smoking status, physical activity, BMI, diet quality, and alcohol intake. Our findings suggested that the associations between the combination of coffee and tea with risk of stroke were more pronounced in individuals aged 50 to 65 years old (*P* for interaction =0.044; S11 Table), but not dementia (*P* for interaction =0.091; S12 Table). There was no significant interaction between coffee, tea, and their combination and sex, smoking status, physical activity, BMI, diet quality, and alcohol intake (all *P* for interaction >0.05). We added these analysis results in supplementary tables (Tables S11-S24), and added descriptions about in sensitive analysis of the results.

Pages 23-24, lines 460-466: “When analyses were stratified by age, the association between the combination of coffee and tea and the risk of stroke was more pronounced in individuals aged 50 to 65 years old (*P* for interaction =0.044; S11 Table), but not dementia (*P* for interaction =0.091; S12 Table). Similar results were obtained when we performed stratified analysis by sex (S13-S14 Tables), smoking status (S15-S16 Tables), alcohol status (S17-S18 Tables), physical activity (S19-S20 Tables), BMI (S21-S22 Tables), and diet pattern (S23-S24 Tables);”

4)     "For covariate information was missing (<20%), we used multiple imputations". Can you provide a table on missing rate of covariates? How many covariates were conducted multiple imputations?
Response: In the present study, ten covariates were conducted with multiple imputations, including HDL, LDL, diet, ethnicity, BMI, smoking status, alcohol status, physical activity, qualification, and income. We have provided a supplementary table (Table S2) and added further description on the missing rate of covariates in the methods section as follows.

Page 15, lines 274-275: “Detailed information on missing data was shown in S2 Table.”

5)     "After excluding participants younger than 50 years old (n =132,168)", Why these participants were excluded from the study? They should be included.
Response: We appreciate the reviewer’s comment. In this study, our primary outcomes were stroke and dementia. Due to most incidents dementia and stroke cases occur in older adults (65 years or older) and the follow-up period was about 15 years, we excluded participants younger than 50 years old. In addition, of 132,168 participants younger than 50 years old, during a median follow-up of 11.4 years for new-onset disease, 143 (0.11%) participants developed dementia, and 904 (0.68%) participants developed stroke. The incidence of stroke and dementia for participants younger than 50 years old was low. Therefore, we excluded participants younger than 50 years old. Following the reviewer’s suggestion, we have included participants younger than 50 years, and described these results in the sensitivity analyses as follows.

Page 24, lines 466-468 & 471: “The results were not much altered compared with those from initial analyses when we repeated analyses: …3) including participants younger than 50 years old (S29–S30 Tables);”

6)     In line 343, endothelial dysfunction is discussed as a potential mechanism linking coffee/tea intake with risk of stroke. Coffee also have anti-oxidant effect and is related to lower cardiometabolic risk, including lipids, type 2 diabetes, and CVD. These should be discussed as well.
Response: We are grateful to the reviewer for the comment. Coffee contains caffeine and is a rich source of antioxidants and other bioactive compounds (*Freedman et al.*

*N Engl J Med, 2012*). Coffee is related to lower cardiometabolic risks, including lipids, type 2 diabetes, and CVD. A published review reported that coffee consumption may lower the risk of type 2 diabetes mellitus and hypertension (*James et al. JAm Coll Cardiol, 2013*). Additionally, growing evidence suggests that coffee consumption is beneficial regarding the risks of CVD (*Ming et al. Circulation, 2014*). Thus, coffee might help prevent stroke by preventing cardiometabolic risks. Following the reviewer’s suggestion, we have added this potential mechanism in the discussion.

Pages 25-26, lines 506-509: “Another potential mechanism maybe that coffee contains caffeine and is a rich source of antioxidants, and evidence demonstrated that coffee was inversely associated with cardiometabolic risk, including CVD, type 2 diabetes, lipids, and hypertension [19, 49, 50].”

7)     In addition, Gelber et al. reported that coffee and caffeine intake in midlife was not associated with cognitive impairment, dementia, and its subtypes. Would the inconsistency in the findings due to sample size?
Response: Thanks for the comment. Gelber et al. conducted the Honolulu-Asia Aging Study included 3494 men found that coffee and caffeine intake in midlife was not associated with overall dementia, Alzheimer’s disease, vascular dementia, or cognitive impairment, which is inconsistent with our findings. As you point out, the cause of this difference might be the sample size. Following the reviewer’s suggestion, we have added a description of this in the discussion.

Page 26, lines 524-528: “Gelber et al. conducted the Honolulu-Asia Aging Study included 3494 men found that coffee and caffeine intake in midlife was not associated with overall dementia, Alzheimer’s disease, vascular dementia, or cognitive impairment, which is inconsistent with our findings. The cause of this difference might be the sample size.”

Minor questions
1)     In figure 1, stroke-dementia is confusing.

Response: We thank the reviewer for pointing out this issue. “stroke-dementia” refers to dementia occurring after stroke. To minimize confusion, we have revised it into “post-stroke dementia” in figure 1.

2)     In figures 2-4, the 95% CI were not fully shown for some categories due to the narrow range of x-axis.

Response: Following the reviewer’s suggestion, we have changed the range of the x-axis in Figures 2-4.

3)     In figures 2-4, please report number of cases and total participants within each category.
Response: We have added the number of cases and total participants within each category in figures 2-4.

**Reviewer #4**: In this manuscript, the authors examine the association of coffee and tea intake with risk of developing stroke, dementia, and post stroke dementia in the large UK biobank study.
The manuscript has a number of strengths including very large size and a prospective design.  Limitations are appropriately described in the discussion and include assessment of coffee and tea by self-report and likely under-ascertainment of stroke and dementia.

We appreciate the reviewer’s comments and suggestions on our manuscript.

I have a number of comments.

1.     The a priori rationale for looking at joint categories of coffee and tea intake is not adequately described or justified. Coffee and tea are distinct beverages with both overlapping and different constituents. Looking through the tables, it looks to me that both coffee and tea intake are associated with these outcomes and I am not convinced that examining joint categories of coffee and tea intake provides much additional insight beyond examining each beverage individually, and furthermore that any differences should not be interpreted as a result of chance.
Response: We thank the reviewer for the comment. Coffee and tea are among the most widely consumed beverages, both in the United Kingdom and worldwide. First, coffee is closely related to tea consumption. A prospective cohort study showed that approximately 70% of participants consumed both coffee and tea (*Yuji et al. BMJ Open Diabetes Res Care, 2020*). Second, coffee and tea are distinct beverages with both overlapping (i.e., caffeine) and different biologically active constituents (i.e., epigallocatechin gallate, chlorogenic acid) (*Sumio et al. Molecules, 2020*). These different compounds have also different target molecules which might lead to different health outcomes (*Hayakawa et al. Molecules, 2020*). Third, genetic polymorphisms in enzymes that involved in uptake, metabolism, and excretion of tea and coffee compounds are also associated with differential biological activities of the two beverages. Furthermore, two studies have found the interaction between coffee and tea on health outcomes. The Japan public health center-based study cohort reported that there was a multiplicative between green tea and coffee with intracerebral hemorrhage (*P* for interaction =0.04) (*Yoshihiro et al. 2013, Stroke*). A prospective study demonstrated that there appears to be an additive interaction between green tea and coffee on mortality in Japanese patients with type 2 diabetes (*Yuji et al. BMJ Open Diabetes Res Care, 2020*). Epidemiological and clinical studies have shown the benefits of coffee and tea separately in preventing dementia. However, little is known about the association between the combination of coffee and tea and the risk of dementia. We hypothesized that there may be multiplicative or additive interaction between coffee and tea with incident stroke and dementia. Therefore, in the current study, we aimed to explore the association between the combination of coffee and tea, which could be the interaction of multiplication or addition, and the risk of stroke and dementia.

According to the reviewer’s suggestion, we have added further description on the reason that this study undertaking an interaction analysis for coffee and tea in the introduction section as follows. Thanks to the reviewer for the valuable comment.

Pages 8-9, lines 122-142: “Coffee is closely related to tea consumption. A prospective cohort study reported that approximately 70% of participants consumed both coffee and tea [20]. Coffee and tea are distinct beverages with both overlapping, such as caffeine, and different biologically active constituents, including epigallocatechin gallate and chlorogenic acid [21]. These constituents appeared to share common mechanisms ― reactive oxygen species, on the other hand, different constituents also have different target molecules and therefore different biological effects [21]. Furthermore, genetic polymorphisms in enzymes that involved in uptake, metabolism, and excretion of tea and coffee components were also associated with the differential biological activities of the two beverages [22]. Additionally, studies have found the interaction between green tea and coffee on health outcomes in the Japanese population [20, 23]. The Japan public health center-based study cohort reported that there was a multiplicative interaction between green tea and coffee that was associated with a lower risk of intracerebral hemorrhage [23]. A prospective study demonstrated that there appear to be an additive interaction between green tea and coffee on mortality in Japanese patients with type 2 diabetes [20]. Epidemiological and clinical studies have shown the benefits of coffee and tea separately in preventing dementia [13-18]. However, little is known about the association between the combination of coffee and tea and the risk of dementia. Therefore, we aimed to explore the association between the combination of coffee and tea, which could be multiplicative or additive interaction, and the risk of stroke and dementia.”

In the current study, we found that drinking coffee and tea separately or in combination were associated with the incidence of stroke and dementia. Furthermore, the combination of coffee and tea seemed to correlate with a lower risk of stroke and dementia compared to coffee or tea separately, and there were multiplicative interactions between coffee and tea on stroke and dementia.

Furthermore, we evaluate the HRs of those who drinking both coffee and tea compared to those who only drinking either coffee or tea. For stroke, after adjustment for confounders, compared with participants who did not drink coffee and tea, HR for those who only drank either coffee or tea was 0.89 (95% CI, 0.86-0.93; *P* <0.001); for dementia, compared with participants who did not drink coffee and tea, HR for those who only drank either coffee or tea was 0.92 (95% CI, 0.87-0.98; *P* =0.0011). These results also suggested that participants who drank both coffee and tea seemed to correlate with lower risk of stroke and dementia compared to those who only drank either coffee or tea.

2.     Coffee and tea intake have been reported to be inversely associated with each other in the cohort (and by the way tea should be added to the coffee stratification in table 1; and coffee to the tea stratum). Also, the correlation between coffee and tea intake in the cohort should be described in the top paragraph of page 12, along with the other factors.
Response: According to the reviewer’s request, we have added tea to the coffee stratification and coffee to the tea stratum in Table 1.

Furthermore, we conducted the correlation analyses among coffee and tea intake and other covariates, results were shown in Supplementary Table S4. We found that coffee intake (cups/day) was associated with tea intake (r=-0.337, *P* <0.001). In addition, both coffee and tea were associated with sex, age, ethnicity, qualification, income, BMI, physical activity, alcohol status, smoking status, sugar-sweetened beverages, LDL, cancer, diabetes, and CAD, but they were not associated with HDL. We added a description of correlations between coffee and tea intake and other covariates in the results.

Page 18, lines 337-340: “Coffee intake (cups/day) was related to tea intake (r=-0.337, *P* <0.001). Both coffee and tea drinking were related to sex, age, ethnicity, qualification, income, BMI, physical activity, alcohol status, smoking status, sugar-sweetened beverages, LDL, cancer, diabetes, and CAD, but not related to HDL (S4 Table).”

It seems possible that cohort members who drink both coffee and tea may differ from non-drinkers of both beverages, as well as exclusive users of either beverage. A table showing baseline characteristics of cohort members who drink both coffee and tea would be a useful addition.
Response: Following the reviewer’s suggestion, we have added a supplementary table (Table S3) to show the baseline characteristics of cohort members who drink both coffee and tea, drink only one beverage, and are non-drinkers of both beverages. Compared to participants who drank neither coffee nor tea, those who drink both beverages were more likely to be older adults, males, whites, former smokers, current drinkers, have a university degree, and have a high income. We have added a description of the baseline characteristics of participants who drink both coffee and tea vs. non-drinkers of both beverages in the results.

Page 18, lines 334-337: “Furthermore, compared to participants who drank neither coffee nor tea, those who drank both beverages were more likely to be older adults, males, whites, former smokers, current drinkers, have a university degree, and have a high income (S3 Table).”

3.     As described in Supplementary Figure S2, only a very small proportion of the cohort drinks neither coffee or tea (well under 5%). Yet, this is used as the reference group for the joint categories of coffee and tea. How many participants in this group? I think it would be useful for the authors to provide the number of participants and events in the figures so that readers can evaluate the stability of these estimates.  
Response: We thank the reviewer for the comment. There were 6703 participants drinks neither coffee nor tea. We performed several additional analyses to assess the robustness of our study, and found that the results were robust (Supplementary Tables 25-34). Following the reviewer’s suggestion, we have added the number of participants and events within each category in figures.

4.     Coffee intake and to some extent tea intake seems to be associated with smoking and alcohol intake. It looks like the authors used relatively broad three-level categories to adjust for potential confounding by each of these exposures. It would be better for the authors to more comprehensively adjust for these important potential confounders. Additionally, the manuscript would benefit from adding in stratified analysis whereby readers can view the associations by stratum of smoking status and alcohol intake.
Response: Following the reviewer’s suggestion, we have more detailed adjustments for smoking and alcohol statuses. Smoking status was categorized as never smokers, former smokers quitted >5 years, former smokers quitted ≤ 5 years, current smokers <10 cigarettes per day, current smokers 10–20 cigarettes per day, and current smokers 20+ cigarettes per day. Alcohol drinking was categorized as never, former, current drinkers <7g per day, current drinkers 7-16 g per day, and current drinkers >16 g per day. Similar results were obtained when we more comprehensively adjusted for smoking and alcohol status. We have added the results of more comprehensive adjustments for smoking and alcohol statuses in Tables S31-32 and described them in the results.

Page 24, lines 466-468 & 471-473: “The results were not much altered compared with those from initial analyses when we repeated analyses: …4) with additional more detail adjustment for smoking and alcohol statuses (S31-S32 Tables);”

We thank the reviewer for the comment. We have conducted stratified analyses by smoking and alcohol status, and found that there is no difference in the results of stratification (all *P* for interaction >0.05). We added these results in supplementary tables (Tables S15-S18). We have also added a statement for sensitivity analyses in Results.

Page 24, lines 463-465: “Similar results were obtained when we performed stratified analyses by sex (S13-S14 Tables), smoking status (S15-16 Tables), alcohol status (S17-S18 Tables)…”

5.     The authors mention the Qian study (24) on page 16 and mention that coffee was not causally related to stroke. However, this analysis uses a mendelian randomization approach which has some strong assumptions. If mentioned, the authors should provide further information about this study including how mendelian randomization approaches may or may not complement more traditional observational epidemiology.
Response: We appreciate the reviewer’s comment. Qian et al. conducted a meta-analysis and adopted Mendelian randomization (MR) approach to evaluate the association between coffee consumption and the risk of stroke and its subtypes. They found that genetically predicted coffee consumption (high vs infrequent/no) was not associated with the risk of stroke. Similarly, among coffee consumers, MR analysis did not indicate causal associations between coffee consumption (cups/day) and risk of stroke. However, in the subgroup analysis, they found weak suggestive evidence for a potential protective role of coffee consumption on the risk of a small vessels, although the association did not reach statistical significance after correction for multiple comparisons.

Indeed, Mendelian randomization (MR) is an increasingly important tool for appraising causality in observational epidemiology. The technique exploits the principle that genotypes are not generally susceptible to reverse causation bias and confounding, reflecting their fixed nature and Mendel’s first and second laws of inheritance. The approach is, however, subject to important limitations and assumptions that, if unaddressed or compounded by poor study design, can lead to erroneous conclusions (*Philip et al. Am J Clin Nutr, 2016*).

In addition, the author reported that since their study used the summary-level data in our MR analyses, they cannot rule out the possibility of a non-linear causal relationship of coffee consumption with the risk of stroke or its subtypes. Indeed, our findings suggested that the associations of coffee and tea with stroke were non-linear (*P* for non-linear <0.001), which may be one reason why our results differ from Qian’s study.

Given the observational epidemiology nature of the present study and the lack of rationale evidence supporting a potential causal interaction, it is difficult to make causal inferences from an observational study. Therefore, it is not appropriate to compare with Qian’s research, so we have deleted this sentence in the revised manuscript.

6.     The authors mention that they lacked information about how coffee was prepared. However, prior studies in the UK Biobank have indicated that there is some data about preparation, including that most of the consumption was instant coffee. <https://biobank.ndph.ox.ac.uk/showcase/field.cgi?id=1508>

Response: We are very grateful for the editors’ comments. In UK Biobank, types of coffee were assessed at baseline using a touchscreen questionnaire. Coffee drinkers were asked “*what type of coffee do you usually drink?*” and were then instructed to select one of six mutually exclusive responses, as follows: “*Decaffeinated coffee (any type)*”, “*Instant coffee*”, or “*Ground coffee (include espresso and filtered coffee)*.” Among coffee drinkers, 160741 (44.0%), 63363 (17.3%), and 57397 (15.7%) participants reported drinking instant, ground, and decaffeinated coffee, respectively. Following the reviewer’s suggestion, we have deleted the sentence in the limitations paragraph of the discussion, and also added information on coffee types in the methods, as follows:

Page 11, lines 186-190: “In addition, coffee drinkers were also asked “what type of coffee do you usually drink?” and were then instructed to select one of six mutually exclusive responses, as follows: “Decaffeinated coffee (any type)”, “Instant coffee”, or “Ground coffee (include espresso and filtered coffee).” We then analyzed the associations among different coffee types and the risk of incident stroke and dementia.”

7.     Additionally, the authors mention that their study included only European individuals and so lack of generalization to other groups is a limitation. Yet, in table 1, non-white groups are mentioned, although I agree their proportion is low. The text should be clarified. Other studies from Asia have examined associations with tea and coffee and these endpoints, including Kokubo which is referenced by the authors (21).

Response: In the present study, ethnicity is categorized as White, Black or Black British, Asian or Asian British, and others. Almost 96% of participants were Whites (96%). Based on the reviewer’s comment, we have revised our manuscript as below.

Page 29, lines 585-587: “Finally, our findings may only be generalizable to demographically similar cohorts, and this limitation precludes the generalization of these findings to the general population”

We appreciate the efforts of reviewers and the editors for their efforts on this manuscript. We benefited from their comments and suggestions.
