# Supplementary material for: Consumption of coffee and tea and risk of developing stroke, dementia, and poststroke dementia: A cohort study in the UK Biobank
Source: PLoS Med. 2021 Nov 16;18(11):e1003830. doi: 10.1371/journal.pmed.1003830 (PMC8594796; doi:10.1371/journal.pmed.1003830)
Supplement: Attachment — Submitted filename: Response to editors and reviewers-R2.doc [file pmed.1003830.s042.doc]

Re: Manuscript ID: PMEDICINE-D-21-00534R2 “*Association of coffee and tea with the risk of developing stroke, dementia, and post-stroke dementia: A prospective cohort study*”

Dear Editor,

Thank you very much for your email regarding the revision of our manuscript “**Association of coffee and tea with the risk of developing stroke, dementia, and post-stroke dementia: A prospective cohort study**” (Manuscript ID: PMEDICINE-D-21-00534R2).

We greatly thank the editors and the reviewers for their valuable comments. These comments are of great significance to our research. We have revised our manuscript according to the editors’ and the reviewers’ comments, and the amendments are highlighted in red in the revised manuscript. A point-to-point response to the editors’ and reviewers’ comments was attached as a separate file.

We believe that the revised manuscript is very much improved by the revisions. We appreciate you and the reviewers’ great help and efforts on this manuscript. We kindly hope the revised manuscript is acceptable for publication in *PLOS Medicine*.

Yours sincerely

Yaogang Wang, PhD (Corresponding author)

Professor

School of Public Health

Tianjin Medical University

Qixiangtai Road 22, Heping District, 300070, Tianjin, China

Phone: +86 22 8333 6636; Email: YaogangWANG@tmu.edu.cn

**Response to editors’ and reviewers’ comments**

The authors would like to thank the editors and reviewers for their constructive and positive comments on our manuscript entitled “Association of coffee and tea with the risk of developing stroke, dementia, and post-stroke dementia: A prospective cohort study” (Manuscript ID: PMEDICINE-D-21-00534R2). We have made essential revisions based on the editors’ and reviewers’ comments. Point by point responses to the editors’ and reviewers’ comments are listed as below.

**Requests from Editors:**

1. From the academic editor: The comments of the third reviewer do not necessarily need to be addressed, but the comments about the limitations section needs to be expanded in both the discussion and the abstract; the section only includes 3 weaknesses and these should be described more in detail and the conclusions should be tempered by the low absolute risk and the likely residual confounding. Given the low absolute proportions of participants who developed the primary events there is likely to be residual confounding based on the baseline demographics and risk factors, as well as the unmeasured confounding on healthy lifestyle that could more likely occur in some types of tea and coffee drinkers.

Response: We thank the academic editor for the comments. We have expanded the limitations section: 1) Coffee and tea intakes were self-report at baseline, which may not reflect long-term consumption patterns. Potential changes in coffee and tea consumptions after the baseline examination may have influenced our risk estimates. Future research is needed to investigate the impact of changes in coffee and tea intake over time on stroke and dementia risk. 2) Coffee and tea intakes are all self-reported measures, which could lead to inaccurate responses, although most large epidemiological studies rely on self-reported questionnaires. 3) People who volunteer for the UK Biobank cohort tend to be, on average, more health-conscious than nonparticipants, which may lead to underestimation prevalence and incidence of stroke and dementia. Fry et al. reported that UK Biobank participants generally live in less socioeconomically deprived areas; are less likely to be obese, to smoke, and to drink alcohol; and have fewer self-reported health conditions, with evidence of a “healthy volunteer” selection bias (*Fry et al., Am J Epidemiol, 2017*). 4) Similar to most observational studies, the bias that may be caused by unmeasured confounding factors remains (e.g., mental health, sleep pattern, and genetic predisposition), even though multiple sensitivity analyses have been carried out in the current study. Furthermore, given the low absolute proportions of participants who developed the primary events, there is likely to be residual confounding based on the baseline demographics and risk factors, as well as the unmeasured confounding on healthy lifestyle that could more likely occur in some types of tea and coffee drinkers. Thus, the conclusions could be tempered by the low absolute risk and the likely residual confounding. 5) Since most of the UK Biobank participants were of white British (96%), our findings may only be generalizable to demographically similar cohorts, and this limitation precludes the generalization of these findings to the general population. Following the editors’ suggestion, we have expanded the limitations in the abstract and discussion, as follows.

Page 3, lines 54-57: “The main limitations were that coffee and tea intakes were self-report at baseline, which may not reflect long-term consumption patterns, unmeasured confounders in observational studies result in biased effect estimates, and participants were not represent the whole UK population.”

Pages 26-28, lines 534-558: “First, coffee and tea intakes were self-report at baseline, which may not reflect long-term consumption patterns. Potential changes in coffee and tea consumptions after the baseline examination may have influenced our risk estimates. Future research is needed to investigate the impact of changes in coffee and tea intake over time on stroke and dementia risk. Second, coffee and tea intakes are all self-reported measures, which could lead to inaccurate responses, although most large epidemiological studies rely on self-reported questionnaires. Third, people who volunteer for the UK Biobank cohort tend to be, on average, more health-conscious than nonparticipants, which may lead to underestimation prevalence and incidence of stroke and dementia [56]. Fry et al. reported that UK Biobank participants generally live in less socioeconomically deprived areas; are less likely to be obese, to smoke, and to drink alcohol; and have fewer self-reported health conditions, with evidence of a “healthy volunteer” selection bias [56]. Fourth, similar to most observational studies, the bias that may be caused by unmeasured confounding factors remains (e.g., mental disease, sleep pattern, and genetic predisposition), even though multiple sensitivity analyses have been carried out in the current study. Furthermore, given the low absolute proportions of participants who developed the primary events, there is likely to be residual confounding based on the baseline demographics and risk factors, as well as the unmeasured confounding on healthy lifestyle that could more likely occur in some types of tea and coffee drinkers. Thus, the conclusions could be tempered by the low absolute risk and the likely residual confounding. Finally, since most of the UK Biobank participants were of white British (96%), our findings may only be generalizable to demographically similar cohorts, and this limitation precludes the generalization of these findings to the general population.”

2. Title: Please revise the title to: “Consumption of coffee and tea and risk of developing stroke, dementia, and poststroke dementia: A cohort study in the UK Biobank”

Response: Following the editors’ suggestion, we have revised our title, as following:

Title: Consumption of coffee and tea and risk of developing stroke, dementia, and poststroke dementia: A cohort study in the UK Biobank

3. Data availability statement: Please revise slightly to: “Data from the UK Biobank cannot be shared publicly however data are available from the UK Biobank Institutional Data Access / Ethics Committee (contact via http://www.ukbiobank.ac.uk/ or contact by email at access@ukbiobank.ac.uk) for researchers who meet the criteria for access to confidential data.”

Response: According to the editors’ suggestion, we have revised our data availability statement as below:

Data availability statement: “Data from the UK Biobank cannot be shared publicly, however, data are available from the UK Biobank Institutional Data Access / Ethics Committee (contact via http://www.ukbiobank.ac.uk/ or contact by email at access@ukbiobank.ac.uk) for researchers who meet the criteria for access to confidential data.”

4. Abstract: Methods and Findings: Please clarify here to mention you are looking for associations between coffee/tea consumption and stroke “We used Cox proportional hazards models to estimate hazard ratios (HRs) and 95% confidence intervals (CIs)...”

Response: Following the editors’ comments, we have amended the sentence as following:

Page 2, lines 30-32: “We used Cox proportional hazards models to estimate the associations between coffee/tea consumption and incident stroke and dementia …”

5. Abstract: Methods and Findings: “Please change “sugar-sweetened beverages” to “consumption of sugar-sweetened beverages”, if accurate.

Response: We appreciate the editors’ careful review. We have replaced “sugar-sweetened beverages” with “consumption of sugar-sweetened beverages” in the revised manuscript (page 2, line 34).

6. Abstract: Methods and Findings: Please revise to indicate the direction of the association, for example, was the combination of coffee and tea consumption associated with lower risks?: “Moreover, the combination of coffee and tea consumption was associated with the risks of ischemic stroke and vascular dementia. Additionally, the combination of tea and coffee was associated with post-stroke dementia, with the lowest risk of incident post-stroke dementia at a daily consumption level of 3-6 cups of coffee and tea (HR, 0.52, 95% CI, 0.32-0.83; P =0.007).”

Response: Thanks to the editors for the professional comments. We have revised the sentence as follows.

Page 3, lines 49-54: “Moreover, the combination of coffee and tea consumption was associated with lower risk of ischemic stroke and vascular dementia. Additionally, the combination of tea and coffee was associated with a lower risk of post-stroke dementia, with the lowest risk of incident post-stroke dementia at a daily consumption level of 3-6 cups of coffee and tea (HR, 0.52, 95% CI, 0.32-0.83; *P* =0.007).”

Page 3, lines 60-63: “We found that drinking coffee and tea separately or in combination were associated with lower risk of stroke and dementia. Intake of coffee alone or in combination with tea was associated with lower risk of post-stroke dementia.”

7. Abstract: Methods and Findings: In the final limitations sentence, please expand the discussion of the main limitations of the study, as pointed out by the reviewer and academic editor.

Response: We appreciate the editors’ comment. Following the editors’ suggestion, we have expanded the discussion of the main limitations in point #1 above, including: (1) coffee and tea intakes were self-report at baseline, which may not reflect long-term consumption patterns. Potential changes in coffee and tea consumptions after the baseline examination may have influenced our risk estimates. Future research is needed to investigate the impact of changes in coffee and tea intake over time on stroke and dementia risk. (2) Coffee and tea intakes are all self-reported measures, which could lead to inaccurate responses, although most large epidemiological studies rely on self-reported questionnaires. (3) People who volunteer for the UK Biobank cohort tend to be, on average, more health-conscious than nonparticipants, which may lead to underestimation prevalence and incidence of stroke and dementia. Fry et al. reported that UK Biobank participants generally live in less socioeconomically deprived areas; are less likely to be obese, to smoke, and to drink alcohol; and have fewer self-reported health conditions, with evidence of a “healthy volunteer” selection bias (*Fry et al., Am J Epidemiol, 2017*). (4) Similar to most observational studies, the bias that may be caused by unmeasured confounding factors remains (e.g., mental health, sleep pattern, and genetic predisposition), even though multiple sensitivity analyses have been carried out in the current study. Furthermore, given the low absolute proportions of participants who developed the primary events, there is likely to be residual confounding based on the baseline demographics and risk factors, as well as the unmeasured confounding on healthy lifestyle that could more likely occur in some types of tea and coffee drinkers. Thus, the conclusions could be tempered by the low absolute risk and the likely residual confounding. (5) Since most of the UK Biobank participants were of white British (96%), our findings may only be generalizable to demographically similar cohorts, and this limitation precludes the generalization of these findings to the general population. According to the editors’ comments, we have revised the main limitations in the abstract and discussion, as follows.

Abstract:

Page 3, lines 54-57: “The main limitations included coffee and tea intakes were self-report at baseline, which may not reflect long-term consumption patterns, unmeasured confounders in observational studies result in biased effect estimates, and participants were not represent the whole UK population.”

Discussion:

Pages 26-28, lines 534-558: “First, coffee and tea intakes were self-report at baseline, which may not reflect long-term consumption patterns. Potential changes in coffee and tea consumptions after the baseline examination may have influenced our risk estimates. Future research is needed to investigate the impact of changes in coffee and tea intake over time on stroke and dementia risk. Second, coffee and tea intakes are all self-reported measures, which could lead to inaccurate responses, although most large epidemiological studies rely on self-reported questionnaires. Third, people who volunteer for the UK Biobank cohort tend to be, on average, more health-conscious than nonparticipants, which may lead to underestimation prevalence and incidence of stroke and dementia [56]. Fry et al. reported that UK Biobank participants generally live in less socioeconomically deprived areas; are less likely to be obese, to smoke, and to drink alcohol; and have fewer self-reported health conditions, with evidence of a “healthy volunteer” selection bias [56]. Fourth, similar to most observational studies, the bias that may be caused by unmeasured confounding factors remains (e.g., mental disease, sleep pattern, and genetic predisposition), even though multiple sensitivity analyses have been carried out in the current study. Furthermore, given the low absolute proportions of participants who developed the primary events, there is likely to be residual confounding based on the baseline demographics and risk factors, as well as the unmeasured confounding on healthy lifestyle that could more likely occur in some types of tea and coffee drinkers. Thus, the conclusions could be tempered by the low absolute risk and the likely residual confounding. Finally, since most of the UK Biobank participants were of white British (96%), our findings may only be generalizable to demographically similar cohorts, and this limitation precludes the generalization of these findings to the general population.”

8. Abstract: Conclusions: Please indicate the direction of the associations in the conclusions.

Response: Following the editors’ suggestion, we have indicated the direction of the associations in the abstract and throughout the manuscript.

Page 3, lines 60-63: “We found that drinking coffee and tea separately or in combination were associated with lower risk of stroke and dementia. Intake of coffee alone or in combination with tea was associated with lower risk of post-stroke dementia.”

9. Author summary: What did the researchers do and find? We suggest revising the first bullet point to: “This study included 365,682 participants (50–74 years old) from the UK Biobank who reported their coffee and tea consumption.” or similar.

Response: We thank the editors for the comment. We have revised the first bullet point in the revised manuscript, as below:

Page 5, lines 76-77: “This study included 365,682 participants (50–74 years old) from the UK Biobank who reported their coffee and tea consumption.”

We suggest revising the third bullet point to: “Drinking 2–3 cups of coffee with 2–3 cups of tea daily was associated with a 32% lower risk of stroke and a 28% lower risk of dementia”

Response: Following the editors’ comments, we have revised the sentence as below:

Page 5, lines 81-83: “Drinking 2–3 cups of coffee with 2–3 cups of tea daily was associated with a 32% lower risk of stroke and a 28% lower risk of dementia”

10. Author summary: What do these findings mean?: We suggest revising the final bullet point to: “These findings may be of interest to clinicians involved in the prevention and treatment of stroke, dementia, and post-stroke dementia”

Response: Following the editors’ comments, we have revised the sentence as below:

Page 6, lines 91-93: “These findings may be of interest to clinicians involved in the prevention and treatment of stroke, dementia, and post-stroke dementia.”

11. Introduction: Please revise “Coffee is closely related to tea consumption” to read “Coffee consumption is closely related…” if accurate.

Response: We thank the editors for the corrections. We have replaced “Coffee is closely related to tea consumption” with “Coffee consumption is closely related to tea consumption” in the revised manuscript (page 8, line 125).

12. Introduction: Please clarify what is meant, or revise to: “Coffee and tea are distinct beverages with overlapping components, such as caffeine, and different biologically active constituents, including epigallocatechin gallate and chlorogenic acid [21].” or similar.

Response: Following the editors’ suggestion, we have revised the sentence as below:

Page 8, lines 127-129: “Coffee and tea are distinct beverages with overlapping components, such as caffeine, and different biologically active constituents, including epigallocatechin gallate and chlorogenic acid [14].”

13. Methods: Please revise to “The analysis plan was drafted prospectively in February 2020 (S1 Text).” if this is accurate.

Response: We have revised the sentence in methods as below:

Page 10, lines 164-165: “The analysis plan was drafted prospectively in February 2020 (S1 Text).”

14. Methods: Please clarify the six mutually exclusive responses for types of coffee because it is not clear how the responses listed here correspond with six possibilities: (“...instructed to select one of six mutually exclusive responses, as follows: “Decaffeinated coffee (any type)”, “Instant coffee”, or “Ground coffee (include espresso and filtered coffee).” For tea, please note if any information on “type of tea” was collected after the number of cups per day.

Response: We apologize for the mistake. In the UK Biobank, coffee drinkers were also asked “what type of coffee do you usually drink?” and were then instructed to select one of six mutually exclusive responses, as follows: “Decaffeinated coffee (any type)”, “Instant coffee”, “Ground coffee (include espresso and filtered coffee), “other type of coffee”, “Do not know”, or “prefer not to answer”. We have added descriptions on types of coffee in methods as follows:

Page 11, lines 185-189: “In addition, coffee drinkers were also asked “what type of coffee do you usually drink?” and were then instructed to select one of six mutually exclusive responses, as follows: “Decaffeinated coffee (any type)”, “Instant coffee”, “Ground coffee (include espresso and filtered coffee), “other type of coffee”, “Do not know”, or “prefer not to answer”.”

In UK Biobank, tea intake was assessed at baseline using a touchscreen questionnaire. Participants were asked, “how many cups of tea do you drink each day (including black and green tea)?”. However, the information on “type of tea” was not collected after the number of cups per day. Therefore, due to the limitation of the data, we can’t provide information on types of tea.

15. Methods: In the covariates section, please revise to “consumption of sugar-sweetened beverages” if accurate. Please revise diet pattern to read “healthy and unhealthy” if this is intended.

Response: We appreciate the editors’ careful review, and we have replaced “sugar-sweetened beverages” with “consumption of sugar-sweetened beverages” in the revised manuscript (page 12, line 226; page 14, line 253 & line 268). We have also replaced “health and unhealth” with “healthy and unhealthy” in the revised manuscript (page 13, line 228).

16. Results: Please change “sugar-sweetened beverages” to “consumption of sugar-sweetened beverages” in this section, if accurate.

Response: We have changed “sugar-sweetened beverages” to “consumption of sugar-sweetened beverages” in the results (page 17, line 328).

17. Results: When reporting the associations between coffee/tea intake and stroke/dementia, please mention the direction (higher risk or lower risk) where the association is mentioned (e.g. “coffee intake was associated with stroke”).

Response: We appreciate the editors’ comments. Following the editors’ suggestion, we have indicated the direction where the association is mentioned, as below.

Page 18, lines 342-345: “Besides, the combination of tea and coffee was associated with lower risk of post-stroke dementia, with lowest risk of incident post-stroke dementia at a daily consumption level of 3-6 cups of coffee and tea (HR, 0.52, 95% CI, 0.32-0.83; *P* =0.007).”

Page 18, lines 351-353: “In unadjusted Cox models, coffee and tea intakes were associated with lower risk of stroke (S5 Table). After multivariable adjustment, coffee intake was associated with lower risk of stroke.”

Page 18, lines 356-357: “Likewise, after multivariable adjustment for confounding factors, tea intake was associated with lower risk of stroke.”

Page 19, lines 360-361: “In addition, each coffee and tea were associated with lower risk of ischemic stroke, but not with hemorrhagic stroke”

Page 19, lines 365-366: “…the combination of coffee and tea was associated with lower risk of stroke and ischemic stroke.”

Page 19, lines 375-377: “In unadjusted Cox models, intake of coffee, tea, and their combination were associated with lower risk of dementia and vascular dementia, but not with Alzheimer’s disease (S6 Table).”

Pages 19-20, lines 377-382: “After multivariable adjustment for confounding factors, coffee intake was associated with lower risk of dementia and vascular dementia, but not with Alzheimer’s disease. Likewise, after multivariable adjustment, tea intake was associated with lower risk of dementia and vascular dementia, but not with Alzheimer’s disease.”

Page 20, lines 388-390: “Furthermore, the combination of coffee and tea intake was associated with lower risk of vascular dementia, but not with Alzheimer’s disease.”

Page 21, lines 404-406: “In unadjusted Cox models, coffee and the combination of coffee and tea were associated with lower risk of dementia (S8 Table).”

Page 22, lines 436-442: “Compared to decaffeinated coffee, instant coffee was associated with lower risk of dementia (HR, 0.85; 95% CI, 0.79-0.92; *P* <0.001), Alzheimer disease (HR, 0.81; 95% CI, 0.72-0.91; *P* <0.001), and vascular dementia (HR, 0.84; 95% CI, 0.72-0.99; *P* =0.036); ground coffee was associated with lower risk of dementia (HR, 0.74; 95% CI, 0.66-0.82; *P* <0.001), Alzheimer disease (HR, 0.67; 95% CI, 0.57-0.78; *P* <0.001), and vascular dementia (HR, 0.74; 95% CI, 0.59-0.92; *P* =0.008).”

18. Results: “Next, we assessed the combination of coffee and tea intake on dementia and its subtypes among participants with stroke. We found that the lowest risk of incident dementia was at a daily consumption level of 2–3 cups of coffee and 0 cup of tea.” Please clarify here how this differs from the independent coffee/tea analysis indicating that among coffee drinkers, 2-3 cups of coffee consumed daily was associated with lower risk of dementia, but that no association was identified for tea consumed.

Response: We thank the editors for the comments. When we assessed the combined intake of coffee and tea on dementia among participants with stroke, the reference group was neither coffee nor tea drinkers; while when we assessed the association between independent coffee intake and the risk of dementia, the reference group was non-coffee drinkers; Likewise, when we assessed the association between independent tea intake and the risk of dementia, the reference group was non-tea drinkers (Figure S4). Therefore, the different reference groups cause the combination of coffee and tea analysis to differ from the independent coffee/tea analysis. Following to the editors’ suggestion, we have revised these sentences in the results as follows.

Page 21, lines 406-409: “After multivariable adjustment, compared with non-coffee drinkers, participants who had a daily consumption level of 2–3 cups of coffee were associated with a lower (HR, 0.80; 95% CI, 0.64–0.99; *P* =0.044) risk of dementia, but not with Alzheimer’s disease and vascular dementia.”

Page 21, lines 409-411: “In addition, compared to non-tea drinking, tea intake was not associated with dementia and its subtypes among participants with stroke.”

Page 21, lines 411-419: “Next, we assessed the combination of coffee and tea intake on dementia and its subtypes among participants with stroke. We found that the combination of coffee and tea was associated with lower risk of post-stroke dementia. Compared with those who did not drink coffee and tea, HRs of drinking 0.5-1 cups of coffee and 2-3 cup of tea per day were 0.50 (95% CI, 0.31–0.82; *P* =0.006) for post-stroke dementia.”

19. Results: Please clarify the results for “Similar results were obtained when we performed stratified analysis by sex (S13-S14 Tables), smoking status (S15-S16 Tables), alcohol status (S17-S18 Tables), physical activity (S19-S20 Tables), BMI (S21-S22 Tables), and diet pattern (S23-S24 Tables).” It is not clear what the result are being compared to when described as similar.

Response: To show the robustness of the association between coffee/tea intake and incident stroke and dementia, we conduct a series of stratified analyses. There was no significant interaction between coffee/tea intake and sex, smoking status, physical activity, BMI, diet quality, and alcohol intake (all *P* for interaction >0.05). Therefore, when we performed stratified analysis by sex (S13-S14 Tables), smoking status (S15-S16 Tables), alcohol status (S17-S18 Tables), physical activity (S19-S20 Tables), BMI (S21-S22 Tables), and diet pattern (S23-S24 Tables), results were found to be similar to those from the primary analyses.

Page 23, lines 451-454: “Associations for coffee/tea intake with incident stroke and dementia did not meaningfully differ by sex (S13-S14 Tables), smoking status (S15-S16 Tables), alcohol status (S17-S18 Tables), physical activity (S19-S20 Tables), BMI (S21-S22 Tables), and diet pattern (S23-S24 Tables) (all *P* for interaction > 0.05).”

20. Discussion: Please revise to “...the separate and combined intake of tea and coffee…”

Response: Following the editors’ suggestion, we have revised the sentence as below:

Page 23, lines 464-466: “…the separate and combined intake of tea and coffee were associated with lower risk of stroke, ischemic stroke, dementia, and vascular dementia;”

21. Discussion: The example pointed out in the Results (see point #18 above) does not seem to illustrate point 4 from the introductory paragraph (that combination of coffee and tea was associated with post-stroke dementia.) Please clarify this here as well as in the Conclusion paragraph.

Response: We appreciate the editors’ comments. Similar to point #18 above, the different reference groups (neither coffee nor tea drinkers vs. non-coffee drinkers/non-tea drinkers) cause the combination of coffee and tea analysis to differ from the independent coffee/tea analysis. In independent coffee/tea analysis, coffee was associated with lower risk of post-stroke dementia. In the combination of coffee and tea analysis, we found that coffee alone or the combination of coffee and tea was associated with lower risk of post-stroke dementia (Figure 1, C1-C3). Similar, when we investigated the association between the specified combination of coffee and tea intake categories and post-stroke dementia (Figure S4), we also found that drinking coffee alone or in combination with tea was associated with lower risk of post-stroke dementia. Such as, compared with those who did not drink coffee and tea, HRs of drinking 0.5-1 cups of coffee and 2-3 cup of tea per day were 0.50 (95% CI, 0.31–0.82; *P* =0.006) for post-stroke dementia. Based on above discussion, we have amended the related sentences in the revised manuscript as follows.

Discussion:

Page 24, lines 471-472: “Intake of coffee alone or in combination with tea was associated with lower risk of post-stroke dementia.”

Conclusions:

Page 29, lines 583-585: “drinking coffee alone or in combination with tea was associated with lower risk of post-stroke dementia.”

Abstract (conclusions):

Page 3, lines 62-63: “Intake of coffee alone or in combination with tea was associated with lower risk of post-stroke dementia.”

Author Summary:

Page 5, lines 85-86: “Intake of coffee alone or in combination with tea was associated with lower risk of post-stroke dementia.”

22. Discussion: Please revise this sentence (please avoid the word “preventing”) to avoid implying causality: “Our study showed that there was an interaction between coffee and tea in preventing stroke and dementia”

Response: We thank the editors for pointing out this issue. We have revised terms of using causal language in the discussion, as follows.

Page 25, lines 507-508: “Our study showed that there was an interaction between coffee and tea that associated with stroke and dementia.”

23. Discussion: Please revise this sentence to avoid causal implications. “Our findings highlight the potential benefits of moderate coffee and tea consumption in preventing the risk of stroke and dementia” we instead suggest something such as “Our findings support a potentially beneficial association between moderate coffee and tea consumption and risk of stroke and dementia.”

Response: Following the editors’ suggestion, we have revised the sentence as below:

Page 28, lines 568-570: “Our findings support a potentially beneficial association between moderate coffee and tea consumption and risk of stroke and dementia.”

24. Conclusions: Please note the direction of the relationship between coffee/tea consumption and risk of stroke and dementia. Please revise “Our findings highlight the potential benefits of moderate coffee and tea consumption in preventing stroke and dementia.” to avoid causal implications of the study.

Response: We thank the editors for the comments, and we have revised the sentence as below:

Page 29, lines 586-588: “Our findings support a potentially beneficial association between moderate coffee and tea consumption and risk of stroke and dementia.”

25. Funding, Conflict of Interest, Data availability, Contributions: Please remove

these sections from the main text of the manuscript and ensure all relevant

information is included with the manuscript submission system metadata.

Response: Following the editors’ suggestion, we have removed these sections (Funding, Conflict of Interest, Data availability, Contributions) from the main text of the manuscript, and provided all relevant information in the manuscript submission system metadata.

26. Supporting information file: Throughout, please change the red text to black text. Please avoid the use of bold type in the tables or legends unless the reason for this is made clear. Rather than “former smokers quitted” we suggest “former smokers who have quit” or similar.

Response: According to the editors’ suggestion, we have changed the red text to black text, and have also revised the use of bold type in the tables or legends throughout the supporting information file.

Furthermore, we agree with the reviewer’s comment, and have replaced “former smokers quitted” with “former smokers who have quit” in the revised supporting information file (Pages 56, 57, 59, 60).

27. Supporting information file: Table S2: Please change the text color to black. Please clarify if the N represents number of missing responses. Please define the abbreviations used in the legend.

Response: We appreciate the editors’ comments, we have changed the text color to black. In Table S2, N represents the number of missing responses, and we have described more details about this in legend, as below:

Page 2 in the supporting information file: “N represents the number of missing responses.”

Additionally, we also defined the abbreviations used in the legend, as below.

Page 2 in the supporting information file: “BMI, body mass index (calculated

as weight in kilograms divided by height in meters squared); HDL, high density lipoprotein; LDL, low density lipoprotein.”

**Comments from Reviewers:**

Reviewer #2: Many thanks authors for their great effort to improve the manuscript. All my concerns and comments were professionally addressed by the authors. I am satisfied with the response and revision. Only one very minor point, it says in the conclusions of the abstract: "We found that drinking coffee and tea separately or in combination were associated with the risk of stroke and dementia. Additionally, the combination of tea and coffee was associated with the risk of post-stroke dementia". I can appreciate authors have toned down all the findings into associations which is adequate. However, it should be fine and clearer to say 'associated with lower risk of' instead of 'associated with the risk of' so that readers will know clearly the direction of the associations. This also applies to the whole paper. Just need to agree with the editor and no need to come back to me again. Thanks.

Response: We thank the reviewer for pointing out this issue. We have indicated the direction of the associations throughout the manuscript as follows.

Page 3, lines 49-54: “Moreover, the combination of coffee and tea consumption was associated with lower risk of ischemic stroke and vascular dementia. Additionally, the combination of tea and coffee was associated with lower risk of post-stroke dementia, with the lowest risk of incident post-stroke dementia at a daily consumption level of 3-6 cups of coffee and tea (HR, 0.52, 95% CI, 0.32-0.83; *P* =0.007).”

Page 3, lines 60-63: “We found that drinking coffee and tea separately or in combination were associated with lower risk of stroke and dementia. Intake of coffee alone or in combination with tea was associated with lower risk of post-stroke dementia.”

Page 18, lines 342-345: “Besides, the combination of tea and coffee was associated with lower risk of post-stroke dementia, with lowest risk of incident post-stroke dementia at a daily consumption level of 3-6 cups of coffee and tea (HR, 0.52, 95% CI, 0.32-0.83; *P* =0.007).”

Page 18, lines 351-353: “In unadjusted Cox models, coffee and tea intakes were associated with lower risk of stroke (S5 Table). After multivariable adjustment, coffee intake was associated with lower risk of stroke.”

Page 18, lines 356-357: “Likewise, after multivariable adjustment for confounding factors, tea intake was associated with lower risk of stroke.”

Page 19, lines 360-361: “In addition, each coffee and tea were associated with lower risk of ischemic stroke, but not with hemorrhagic stroke”

Page 19, lines 365-366: “…the combination of coffee and tea was associated with lower risk of stroke and ischemic stroke.”

Page 19, lines 375-377: “In unadjusted Cox models, intake of coffee, tea, and their combination were associated with lower risk of dementia and vascular dementia, but not with Alzheimer’s disease (S6 Table).”

Pages 19-20, lines 377-382: “After multivariable adjustment for confounding factors, coffee intake was associated with lower risk of dementia and vascular dementia, but not with Alzheimer’s disease. Likewise, after multivariable adjustment, tea intake was associated with lower risk of dementia and vascular dementia, but not with Alzheimer’s disease.”

Page 20, lines 388-390: “Furthermore, the combination of coffee and tea intake was associated with lower risk of vascular dementia, but not with Alzheimer’s disease.”

Page 21, lines 404-406: “In unadjusted Cox models, coffee and the combination of coffee and tea were associated with lower risk of dementia (S8 Table).”

Page 22, lines 436-442: “Compared to decaffeinated coffee, instant coffee was associated with lower risk of dementia (HR, 0.85; 95% CI, 0.79-0.92; *P* <0.001), Alzheimer disease (HR, 0.81; 95% CI, 0.72-0.91; *P* <0.001), and vascular dementia (HR, 0.84; 95% CI, 0.72-0.99; *P* =0.036); ground coffee was associated with lower risk of dementia (HR, 0.74; 95% CI, 0.66-0.82; *P* <0.001), Alzheimer disease (HR, 0.67; 95% CI, 0.57-0.78; *P* <0.001), and vascular dementia (HR, 0.74; 95% CI, 0.59-0.92; *P* =0.008).”

Page 23, lines 464-466: “…the separate and combined intake of tea and coffee were associated with lower risk of stroke, ischemic stroke, dementia, and vascular dementia”

Page 29, lines 581-582: “In conclusion, we found that drinking coffee and tea separately or in combination were associated with lower risk of stroke and dementia.”

Reviewer #3: The authors have fully addressed my comments.

Response: We greatly appreciate the reviewer’s encouraging comment.

Reviewer #4: The authors have thoroughly responded to the reviewer and editor comments and I believe have improved the manuscript. I have one remaining comment: I appreciate the straightforward conclusion of the abstract, that drinking coffee and tea separately, and in combination, was associated with lower risk of stroke and dementia. However, I am skeptical that some specified combination of tea and coffee intake truly yields the strongest association. And although the authors note a statistical interaction between coffee and tea drinking for both stroke and dementia, there doesn't seem to be any clear pattern across any of the strata.

For this reason, I think that the extensive presentation of coffee and tea interaction categories in the abstract distracts from the paper. I suggest they should be removed. I also think that chance should be mentioned as a possible explanation for the interaction on page 24 of the discussion.

Response: We are grateful to the reviewer for the comment. We completely understand the reviewer’s concern. According to the review’s suggestion, we have removed sentences that the extensive presentation of coffee and tea interaction categories in the abstract distracts from the paper: “We found a significant interaction between coffee and tea in relation to stroke (*P* <0.001) and dementia (*P* =0.0127)”, and “drinking 0.5–1 cups of coffee with ≥4 cups of tea per day was associated with a 34% (HR, 0.66, 95% CI, 0.57-0.76; *P* <0.001) lower risk of stroke and a 30% (HR, 0.70, 95% CI, 0.58-0.86; *P* <0.001) lower risk of dementia.”

Furthermore, we completely agree with the reviewer that a possible explanation for the interaction between coffee and tea on stroke and dementia may have arisen due to chance. Following the review’s comments, we have added this sentence in the discussion, as below.

Page 26, lines 526-527: “Fourth, the interaction between coffee and tea drinking for both stroke and dementia may have arisen due to chance.”

We appreciate the efforts of editors and reviewers for their valuable comments on this manuscript. We benefited from their comments and suggestions.
